# Lipidomic Profiling of Commercial *Hydnocarpus wightianus* Oil by UPLC-QTOF-MS/MS and GC-MS: Degradation-Associated Lipid Classes as Candidate Quality Attributes for a Non-Pharmacopoeial Excipient

**DOI:** 10.3390/molecules31203582

**Published:** 2026-10-09

**Authors:** Radosław Balwierz, Natalia Schäfer, Patryk Kuropka, Jerzy Wiśniewski, Dorota Wieczorek, Paweł Biernat, Jacek Lipok

**Affiliations:** 1 Institute of Chemistry, University of Opole, 48 Oleska St., 45-052 Opole, Poland; natalia.schafer@uni.opole.pl (N.S.); dorwieczorek@uni.opole.pl (D.W.); jalip@uni.opole.pl (J.L.); 2 Faculty of Chemistry, Wrocław University of Science and Technology, 4/6 Norwida St., 50-373 Wrocław, Poland; patryk.kuropka@pwr.edu.pl (P.K.); jerzy.wisniewski@pwr.edu.pl (J.W.); 3 Department of Drug Forms Technology, Faculty of Pharmacy, Wrocław Medical University, 211 Borowska St., 50-556 Wrocław, Poland; pawel.biernat@umw.edu.pl; 4 Biotts S.A., 44c Wrocławska St., 55-040 Bielany Wrocławskie, Poland

**Keywords:** *Hydnocarpus wightianus*, chaulmoogra oil, lipidomics, UPLC-QTOF-MS/MS, cyclopentenyl fatty acids, pharmaceutical excipient, quality attributes, lipid oxidation

## Abstract

Background/Objectives: Chaulmoogra oil (ChO), from *Hydnocarpus wightianus* Blume seeds, is returning to use as the lipid phase of transdermal carriers, yet no pharmacopoeial monograph governs it. This work asked whether lipid classes associated with hydrolysis and oxidation distinguish oils that a fatty acid specification would not separate. Methods: Three commercial products, each analyzed once, were profiled by ultra-performance liquid chromatography with quadrupole time-of-flight tandem mass spectrometry (UPLC-QTOF-MS/MS), and two of them also by gas chromatography-mass spectrometry (GC-MS). Growth inhibition was tested by broth dilution. Results: Cyclopentenyl acids dominated the fatty acid composition. In one product (ChO 2), fatty acyl esters of hydroxy fatty acids (FAHFA) accounted for about 14% of the annotated negative-mode signal, against 2 and 4% in the others, and diacylglycerols for 1.3% of the positive-mode signal, against 0.3 and 0.7%. The acid value (1.9 to 2.8 mg KOH/g) and the share of oxidized triacylglycerols were instead highest in ChO 3. No measurable growth inhibition of *Escherichia coli*, *Bacillus cereus* or *Candida albicans* by the crude oils was detected up to 250 µg/mL. Conclusions: A specification based on fatty acid composition alone would not separate these products, and the FAHFA and diacylglycerol shares are proposed as candidate attributes associated with hydrolysis and oxidation that require validation.

## 1. Introduction

Chaulmoogra oil (ChO), obtained from the seeds of *Hydnocarpus wightianus* Blume (*Achariaceae*), has been valued for centuries in traditional medicine, particularly for the treatment of leprosy. The binomial used throughout this work is the one under which the material is traded, and current floristic databases treat it as a heterotypic synonym of *Hydnocarpus pentandrus* (Buch.-Ham.) Oken. In the twentieth century, it was officially recognized as a drug and included in both the Indian and British Pharmacopoeias. Its therapeutic activity is primarily attributed to cyclopentenyl fatty acids, chiefly hydnocarpic, chaulmoogric, and gorlic acids [1]. More recently, ChO has been repurposed as a promising functional lipid phase in transdermal drug-delivery systems, for instance in formulations containing methotrexate for psoriasis [2] and in topical formulations for leprosy that carry dapsone or rely on the oil itself [3,4,5]. In a more recent application, the oil was incorporated as a lipid excipient into a multifunctional transdermal carrier for the topical co-delivery of curcumin and berberine, a formulation whose wound-healing efficacy was confirmed in a translational porcine model [6].

Despite these pharmaceutical advances, comprehensive characterization of the chemical composition of ChO remains limited, particularly with respect to its glyceride fractions and fatty acid profile. The oil is not listed in the European or national (e.g., Polish) Pharmacopoeias, which leaves a regulatory gap regarding its standardization. This lack of official monographs is critical because variability in chemical composition between different commercial sources may affect its pharmacological potential and formulation stability. Previous observations have indicated differences in physicochemical parameters, such as colour, density, and solidification point, among oils obtained from the same plant species but supplied by different manufacturers. To address these inconsistencies, the lipidomic profile has to be read alongside the macroscopic properties of the material, which is the approach taken here. For fixed oils that do have a monograph, the European Pharmacopoeia general monograph on vegetable fatty oils and its associated general chapters control identity through the fatty acid composition determined by gas chromatography (2.4.22) and through a thin-layer chromatographic fingerprint (2.3.2), and control the degradation state through the acid value (2.5.1) and the peroxide value (2.5.5) [7]. No compendial specification sets these attributes for chaulmoogra oil, and quality standards for plant oils entering pharmaceutical use remain heterogeneous across sources [8]. The thin-layer method of chapter 2.3.2 separates the intact triacylglycerols rather than their constituent acids, so compendial identity testing already rests in part on the glyceride pattern and not on the fatty acid composition alone [7]. That distinction is directly relevant here, because every reported sample of chaulmoogra oil is dominated by the same three cyclopentenyl acids.

Therefore, this study was designed as a proof of concept. Using three commercially obtained products, the present work tested whether lipid classes usually associated with hydrolysis and oxidation differentiate chaulmoogra oils that share an essentially identical fatty acid profile, and whether such classes are therefore plausible candidates for quality attributes in a future monograph. A second question concerned whether the oil inhibits the growth of common microorganisms, since an excipient that inhibits microbial growth would confound the microbiological assessment of a finished preparation. The investigation employed a combined analytical strategy based on ultra-performance liquid chromatography coupled with tandem mass spectrometry (UPLC-MS/MS) and gas chromatography coupled with mass spectrometry (GC-MS). In addition, a methanolic extraction was performed to recover the polar lipid fraction of each oil, and the resulting extracts were characterized by GC-MS. Since variations in lipid composition may modulate biological activity, the antimicrobial properties of both crude oils and their corresponding methanolic extracts were evaluated against *Escherichia coli*, *Bacillus cereus*, and *Candida albicans* by broth dilution, with two qualitative agar formats reported in the Supplementary Materials. The study is framed as an analytical framework and not as a survey of the market, because three products analyzed once each cannot describe the variability of commercially available chaulmoogra oil.

Two findings are reported that the literature on this oil does not contain. Six lipid classes usually associated with hydrolysis and oxidation are described here for *Hydnocarpus*, namely diacylglycerols (DG), lysophosphatidylcholines (LPC), oxidized triacylglycerols (OxTG), triacylglycerol estolides (TG-EST), fatty acyl esters of hydroxy fatty acids (FAHFA) and oxidized free fatty acids (OxFA), none of which appears in the published characterization of this oil, which was confined to the intact triacylglycerols [9].

The second is that, for the three products examined, the attribute a monograph would control first, the fatty acid composition, did not discriminate between them despite their distinct physicochemical characteristics, whereas several of the minor lipid classes did, and none of these classes is currently controlled for any fixed oil by an untargeted method.

## 2. Results and Discussion

### 2.1. Physicochemical Characterization of Chaulmoogra Oils

The three chaulmoogra oil samples (ChO 1, ChO 2, and ChO 3) exhibited distinct physicochemical characteristics (Table 1). ChO 1 displayed a golden colour of medium intensity and was semi-solid at the temperature of observation, whereas ChO 2 had a cream, light-yellow appearance and ChO 3 was very light yellow, both remaining fluid. The density of the samples ranged between 0.92 and 0.94 g/mL, with ChO 1 showing the lowest value and ChO 2 the highest. The solidification points differed among the samples, namely 16 °C for ChO 1, 15 °C for ChO 2, and 10 °C for ChO 3. As the values for ChO 1 and ChO 2 differ by only 1 °C, their consistency near room temperature is similar and sensitive to small changes in ambient temperature, whereas the markedly lower solidification point of ChO 3 corresponds to its distinctly more fluid character. The solidification temperatures of chaulmoogra oils (10–16 °C) are considerably higher than those of highly unsaturated vegetable oils such as sunflower (−16 to −18 °C) or linseed oil (−16 °C), which remain liquid even under refrigeration. Conversely, chaulmoogra oil remains liquid at temperatures at which saturated fats such as coconut and palm oil are already solid, and the melting ranges reported for those fats are 24 to 25 °C and 30 to 35 °C, respectively [10,11]. The comparison is between a melting range and a solidification point and is therefore indicative only. Chaulmoogra oil occupies an intermediate position, closer to semi-solid fats than to highly unsaturated seed oils [10,11].

Values are the mean ± standard deviation of three determinations on a single lot of each material. Density was determined according to European Pharmacopoeia (Ph. Eur.) general method 2.2.5, the acid value according to ISO 660:2020 [12], and the solidification point by the open-tube tilting method of Section 3.2.

The three products, all traded as oil of the same botanical source (*Hydnocarpus wightianus*), therefore differed in their physicochemical properties. Three products cannot show how large such differences are across the market, but they show that the differences occur, which supports a fuller chemical characterization of the oil before pharmaceutical use. The supplier documentation available for these materials illustrates the regulatory gap, and it does so in a way that is worth setting out in full. The safety data sheet accompanying ChO 1 [13] specifies the product only as vegetable oil triglycerides obtained from chaulmoogra seeds, declares its intended use as personal care, and identifies the material by the Chemical Abstracts Service (CAS) registry number 8001-31-8. That number belongs to coconut oil. Chaulmoogra oil is CAS 8001-74-9 [14]. No fatty acid composition, no acid or peroxide value, no batch identifier and no shelf life are stated. Of the two physical descriptors the document does carry, neither is in line with the material as received: a creamy white solid oil of declared melting point 110 °F, that is about 43 °C, against the golden semi-solid of solidification point 16 °C characterized here. Fats melt at a higher temperature than they solidify, and a gap of a few degrees between the two values is expected, but a gap of about 27 °C is far larger than that phenomenon accounts for. None of the three products was supplied with the compositional attributes that the European Pharmacopoeia requires for vegetable fatty oils [7], and in this instance the oil was supplied under the registry identity of a different oil altogether. A purchaser of these materials could therefore not verify their identity or degradation state before use.

The acid values fell in the range of 1.9 to 2.8 mg KOH/g and increased in the order ChO 1, ChO 2, ChO 3 (Table 1). No limit is set for this material. For context only, and without implying that these limits apply to chaulmoogra oil, the European Pharmacopoeia sets a maximum acid value of 2.0 for virgin almond oil and virgin olive oil and of 4.5 for virgin linseed oil [7].

### 2.2. Lipidomic Profiling by UPLC-MS/MS

**Figure 1:**
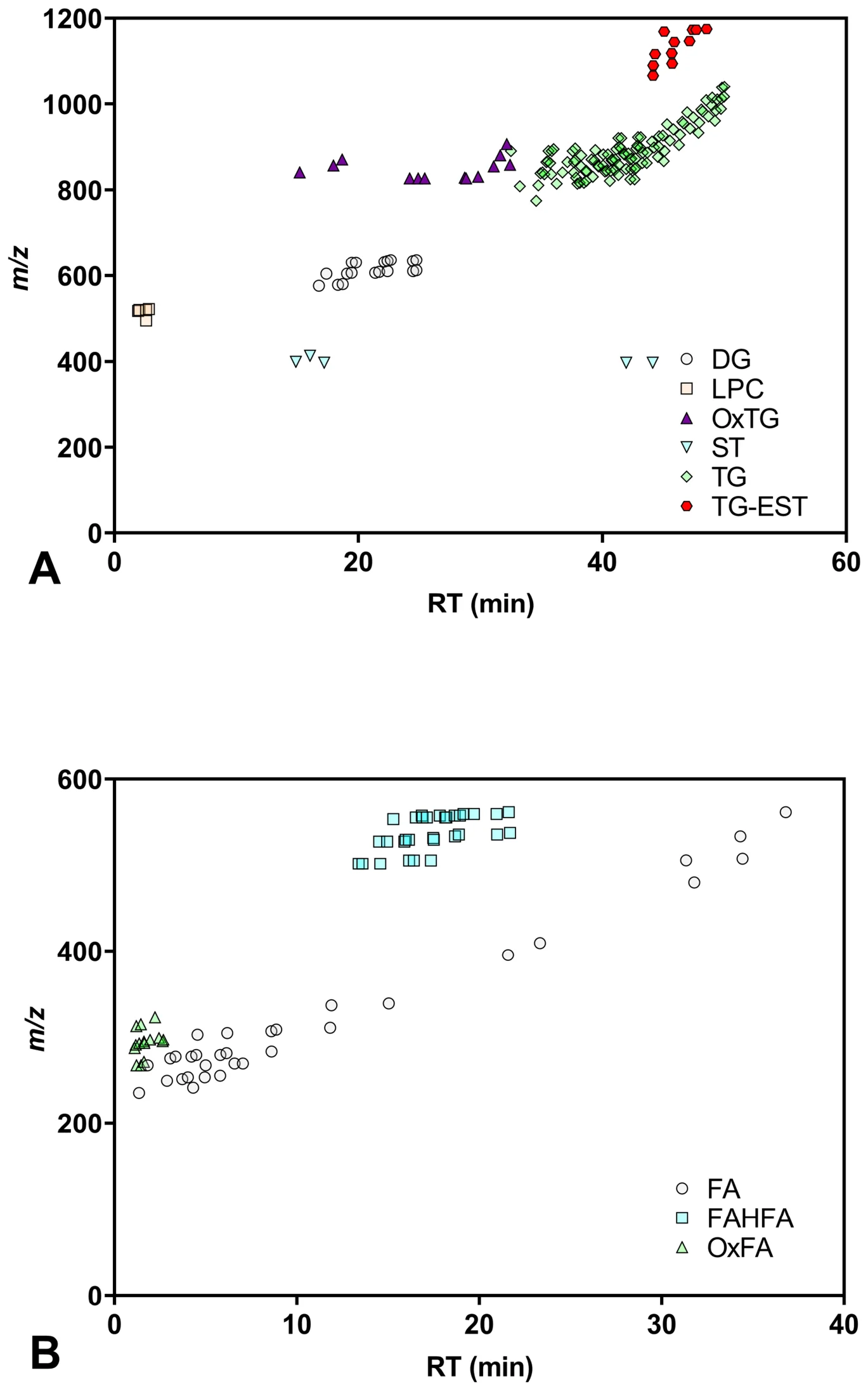
Lipidomic maps displaying the chromatographic distribution of identified molecular features. Data are visualized as mass-to-charge ratio (*m*/*z*) versus retention time (RT) scatter plots, where each point represents one annotated feature (Tables S1 and S2). (**A**) Positive ion mode (POS). Triacylglycerols (TG, green diamonds) elute between 32 and 50 min. Oxidized triacylglycerols (OxTG, purple triangles) elute earlier, whereas triacylglycerol estolides (TG-EST, red hexagons) elute within the triacylglycerol window and are separated from it by mass, above *m*/*z* 1060. Diacylglycerols (DG, open circles), lysophosphatidylcholines (LPC, squares) and sterols (ST, inverted triangles) are also shown. (**B**) Negative ion mode (NEG). Free fatty acids (FA, open circles), fatty acyl esters of hydroxy fatty acids (FAHFA, teal squares), which form a cluster between 13 and 22 min, and oxidized free fatty acids (OxFA, green triangles). Abbreviations: DG, diacylglycerol. FA, fatty acid. FAHFA, fatty acyl ester of hydroxy fatty acid. LPC, lysophosphatidylcholine. NEG, negative ion mode. OxFA, oxidized free fatty acid. OxTG, oxidized triacylglycerol. POS, positive ion mode. RT, retention time. ST, sterol. TG, triacylglycerol. TG-EST, triacylglycerol estolide.

UPLC-MS/MS analyses were conducted in both positive and negative ion modes to achieve comprehensive lipidomic coverage. A total of 162 lipid species were annotated in positive ion mode, including triacylglycerols (TG, 110 analytes), diacylglycerols (DG, 18 analytes), oxidized triacylglycerols (OxTG, 13 analytes), triacylglycerol estolides (TG-EST, 11 analytes), lysophosphatidylcholines (LPC, 5 analytes), and sterols (ST, 5 analytes) (Figure 1A, Table S1). Negative ion mode enabled the annotation of 78 lipids, predominantly free fatty acids (FA, 32 analytes), fatty acyl esters of hydroxy fatty acids (FAHFA, 31 analytes), and oxidized free fatty acids (OxFA, 15 analytes) (Figure 1B, Table S2).

The annotation confidence assigned to each species is reported in the Lipid identification level column of Tables S1 and S2, which also list the measured and the reference *m*/*z* of every feature. Of the 162 positive-mode annotations, 110 were assigned to the higher confidence level, meaning that the acyl chain composition, where present, was resolved by MS/MS at the molecular species level, and 52 to the level that, due to chromatographic overlap and the resulting spectral chimerism, admits an alternative fatty acyl composition, whereas all 78 negative-mode annotations were assigned to the higher level. Across the annotated species, the median absolute mass error was 1.4 ppm in the positive mode and 1.2 ppm in the negative mode. In the positive mode no feature exceeded 6.1 ppm. In the negative mode three features exceeded 10 ppm, with a maximum of 27.1 ppm. These are the three most intense features of that mode, marked in Table S2, which is the behaviour expected when the detector response approaches saturation. Positive-mode annotation relied on the ammonium adduct for 152 of the 162 species, and negative-mode annotation on the deprotonated molecule throughout.

The annotated TG and DG species contained 45 to 64 and 32 to 36 total acyl carbons, respectively, corresponding to the sums of their esterified fatty acyl chains. At the individual-chain level, the fatty acyl residues were predominantly C16–C18 species with the degree of unsaturation value up to four, as supported by the MS/MS fragmentation data shown in Figures S3–S9. The five annotated sterols contained 28 to 29 carbon atoms with up to two double bonds, and this class requires a qualification. Three of the five carry the same annotation, ST 29:1;O, at retention times of 17.20, 41.96 and 44.13 min. A free sterol cannot span 27 min on this gradient. All three features carrying this annotation were assigned from the water-loss ion [M+H−H_2_O]^+^, and the two late ones fall inside the triacylglycerol elution window (32.5 to 50.0 min), so they are more readily explained as in-source fragments or as steryl esters than as free sterols. The sterol class is therefore reported for completeness of the annotation output and is not used for any comparison between samples. For several sum compositions the annotation returned the same acyl assignment at two or more retention times, and the intensities within such a set are strongly asymmetric. TG 54:6, TG 54:7 and TG 54:9 are the clearest cases: the features span about 5 min and, summed over the three oils, the latest carries 15 to 75 times the intensity of the earliest (Table S1). In the TG 54:6 set this holds for the earliest and the latest feature, the intermediate one being annotated differently. These sets are not interpreted here as cyclopentenyl and straight-chain isobar pairs. A separation of 5 min is four to ten times larger than the 0.5 to 1.3 min shift documented earlier for such pairs [9] (Section 3.3.2, Table 3 of that work). The gas chromatographic composition offers no straight-chain counterpart either, since α-linolenic acid was not detected in either oil and linoleic acid did not exceed 1.9% (Table 2). Positional isomers of the ring, sn-regioisomers arising from acyl migration and in-source fragments of the higher-mass estolides all remain possible, and none of them is separable with the present data. The asymmetry is not general either. TG 52:8, the most intense triacylglycerol of the whole dataset, returns three features of identical assignment at 35.41, 37.14 and 37.67 min, of which the middle one dominates.

Lysophosphatidylcholines (1-acyl-sn-glycero-3-phosphocholines) are monoacyl phospholipids commonly formed by hydrolysis of the sn-2 acyl chain of phosphatidylcholines. LPCs were detected in all samples, with the annotated species including LPC 18:3, LPC 18:2, LPC 18:1, and LPC 16:0. Because LPC shorthand notation reports only the total acyl carbon number and degree of unsaturation, the LPC 18:3 annotation does not distinguish between isomeric 18:3 fatty acyl residues. In particular, α-linolenic acid and the cyclopentenyl fatty acid gorlic acid share the same molecular formula, C_18_H_30_O_2_, and therefore cannot be differentiated by accurate mass alone. Given the high abundance of cyclopentenyl acids indicated by GC-MS analysis of these oils, the 18:3 acyl residue in LPC 18:3 likely includes, at least in part, gorlic acid rather than α-linolenic acid. The biological activities of these compounds may differ depending on the structural characteristics of the fatty acid moiety [15,16,17]. The chromatographic evidence is equivocal on this point. A single LPC 18:3 feature was detected at a retention time of 1.94 min, whereas the same separation resolved two LPC 18:2 features at 2.05 min and at 2.60 min (Table S1). Two readings cannot be separated. The two 18:2 features may be acyl-chain isomers, in which case the method resolves them and the single 18:3 feature represents one predominant isomer, or they may be sn-1 and sn-2 regioisomers formed by acyl migration, in which case the separation carries no information about the acyl chain. A retention argument does not resolve the question here, because cyclopentenyl species elute after their straight-chain isobars [9], whereas the single 18:3 feature is the earliest of the five annotated lysophosphatidylcholine features. The assignment therefore rests on the GC-MS data. Gorlic acid accounted for 18.9% of the total chromatographic peak area of ChO 2 and 20.8% of that of ChO 3, whereas no peak was detected at the retention index of methyl linolenate in either oil, so α-linolenic acid remained below the limit of detection (Table 2) and did not exceed 0.9% in the previously reported ChO 1 methanolic extract [18]. In the absence of an authentic gorlic acid standard, the assignment cannot be made definitive, and the species is therefore reported at the sum-composition level.

The triacylglycerol estolides annotated in this study represent an additional class of structurally complex lipids. These species consist of triacylglycerols bearing ester-linked fatty acid substituents attached through hydroxyl groups, resulting in branched acyl structures. Estolides of this type have been described in several plant oils [19,20]. The complete list of annotated estolide species, including their *m*/*z* values and signal intensities, is provided in Table S1. The untargeted annotation of the three oils was complemented by the targeted fragmentation experiment of Section 3.4.2. Twelve triacylglycerol precursors were selected and confirmed, together with nine diacylglycerols, built from five and four different fatty acids, respectively. Every one of the twelve triacylglycerols carried at least one cyclopentenyl acyl, and eleven of them were composed of cyclopentenyl acids alone, with the exception of one palmitic residue. The experiment also returned a species consistent with aleprylic acid, a twelve-carbon cyclopentenyl homologue that the untargeted annotation did not report, and the triacylglycerol composed of three hydnocarpic acyls, which is likewise absent from Table S1. In one instance, the two approaches disagreed: the feature at *m*/*z* 866.72 was annotated as TG 52:7 with the composition 16:1_18:3_18:3, whereas its fragmentation returned 16:2_18:2_18:3 (Figure S4). For the coverage obtained, 110 triacylglycerols were annotated here against 136 reported for this oil by non-aqueous reversed-phase chromatography with high-resolution mass spectrometry, of which 71 carried at least one cyclopentenyl acyl [9]. Selecting precursors individually therefore recovers species that the untargeted workflow did not annotate, and the two approaches are complementary. The assigned diacylglycerols share fragmentation pathways with triacylglycerols, but they can be differentiated by their distinct retention times from the triacylglycerols with which they share acyl chains, and they carry their own adducts, which an in-source fragment would not.

Free fatty acids detected in negative ion mode ranged from C15 to C38 and contained 0–4 double bond equivalents. The annotated FAHFAs consisted of two acyl components, most commonly involving 18:2, 18:3, and 16:2 residues, with individual chains ranging from C16 to C18 and having up to three nominal degrees of unsaturation. Summed over both acyl chains, these FAHFA species corresponded to total carbon numbers of 32 to 36 and to 0 to 6 double bond equivalents (1 to 7 in the species-level names of Table S2, which count the ester carbonyl, Section 3.4.1). Six of them carry the prefix AAHFA assigned by the annotation software, which denotes an acyl alpha-hydroxy fatty acid [21], and they are counted within the FAHFA class.

### 2.3. Fatty Acid Composition by GC-MS

GC-MS analysis of fatty acid methyl esters (FAMEs) confirmed that cyclopentenyl fatty acids were the predominant fatty acyl class in all oil samples. ChO 1 had been characterized previously [18]. In the present study, GC-MS analysis was applied to ChO 2 and ChO 3 and their methanolic extracts. The obtained results are summarized in Table 2. Representative GC-MS chromatograms of oil samples and their methanolic extracts are shown in Figures S10–S13. ChO 1 was characterized in a previous study of this laboratory using the same instrument, the same pair of stationary phases and the same derivatization protocol, which makes the values directly comparable with those obtained here. Peak assignments for Figures S10–S13, listed in order of elution and numbered as in Table 2, are given in Table S3.

Expressed as percentages of the total chromatographic peak area, ChO 2 contained hydnocarpic acid (30.0%), chaulmoogric acid (23.2%), and gorlic acid (18.9%), together 72.1%, which corresponds to 80.9% of the identified fatty acid methyl esters. ChO 3 showed a comparable profile, with hydnocarpic acid (32.1%), chaulmoogric acid (23.8%), and gorlic acid (20.8%), together 76.7%, or 84.5% of the identified fatty acid methyl esters. These results are consistent with previous reports for ChO 1 (79.8%) [18].

Minor constituents included methyl palmitate, methyl oleate, methyl linoleate, and other unsaturated C16–C18 fatty acids. Linoleic acid was not detected in ChO 3, whereas its methanolic extract carried 2.3%, and the reverse pattern was obtained for ChO 2 (1.9% in the oil, not detected in the extract). Both observations sit at the same retention time. Methyl octadec-11-enoate (peak 7) and methyl linoleate (peak 6), with experimental linear retention indices (LRIexp) of 2105 and 2104, co-elute at 22.5 min on the ZB-5HT column and may partially overlap (Table S3), so the assignment between these two peaks is not secure in either direction, and no interpretation of oxidative stability is drawn from it here.

Methanolic extracts of ChO 2 and ChO 3 contained lower proportions of cyclopentenyl fatty acids compared with the crude oils. The ChO 2 extract contained 55.7% and the ChO 3 extract 57.6% cyclopentenyl fatty acids as percentages of the total chromatographic peak area, or 73.6% and 71.5% of the identified fatty acid methyl esters, in contrast to 83.5% reported for the ChO 1 extract [18]. The origin of that difference deserves comment. Acid-catalyzed methanolysis converts triacylglycerols, diacylglycerols and free fatty acids alike into the same fatty acid methyl esters, so partial hydrolysis of the oil cannot by itself alter the FAME distribution. The lower cyclopentenyl proportion in the ChO 2 and ChO 3 extracts is more plausibly attributable to two other contributions: acyl chains modified by oxidation, which no longer elute as the parent methyl esters, and co-extracted material that yields no FAME at all. The unassigned share of the chromatographic peak area is compatible with this reading, amounting to 24.3% in the ChO 2 extract and 19.4% in the ChO 3 extract against 10.9% and 9.2% in the corresponding oils (Table 2).

### 2.4. Comparative Analysis Among Chaulmoogra Oils

Although all three oils shared the same lipid classes and species, their relative proportions differed. A heatmap of 50 annotated species of both ion modes across the three oils is provided in Figure S14.

Across all three oils, the fatty acyls most frequently composing the identified lipids were 18:2, 18:3, and 16:2. This is in close agreement with the GC-MS fatty acid profile, in which the cyclopentenyl acids chaulmoogric (18:2), gorlic (18:3), and hydnocarpic (16:2) predominate. These signals therefore correspond predominantly to the cyclopentenyl fatty acids rather than to their straight-chain isomers. The concordance was evident among the most intense species. The five most intense triacylglycerols were combinations of these acyls (TG 16:2_18:2_18:2, TG 18:2_18:2_18:3, TG 16:2_18:3_18:3, TG 18:2_18:3_18:3, and TG 18:2_18:2_18:2), and the three most intense free fatty acids were 18:3, 18:2, and 16:2. Taken together, this indicates that the major lipids of chaulmoogra oil are built predominantly from cyclopentenyl fatty acids.

**Figure 2:**
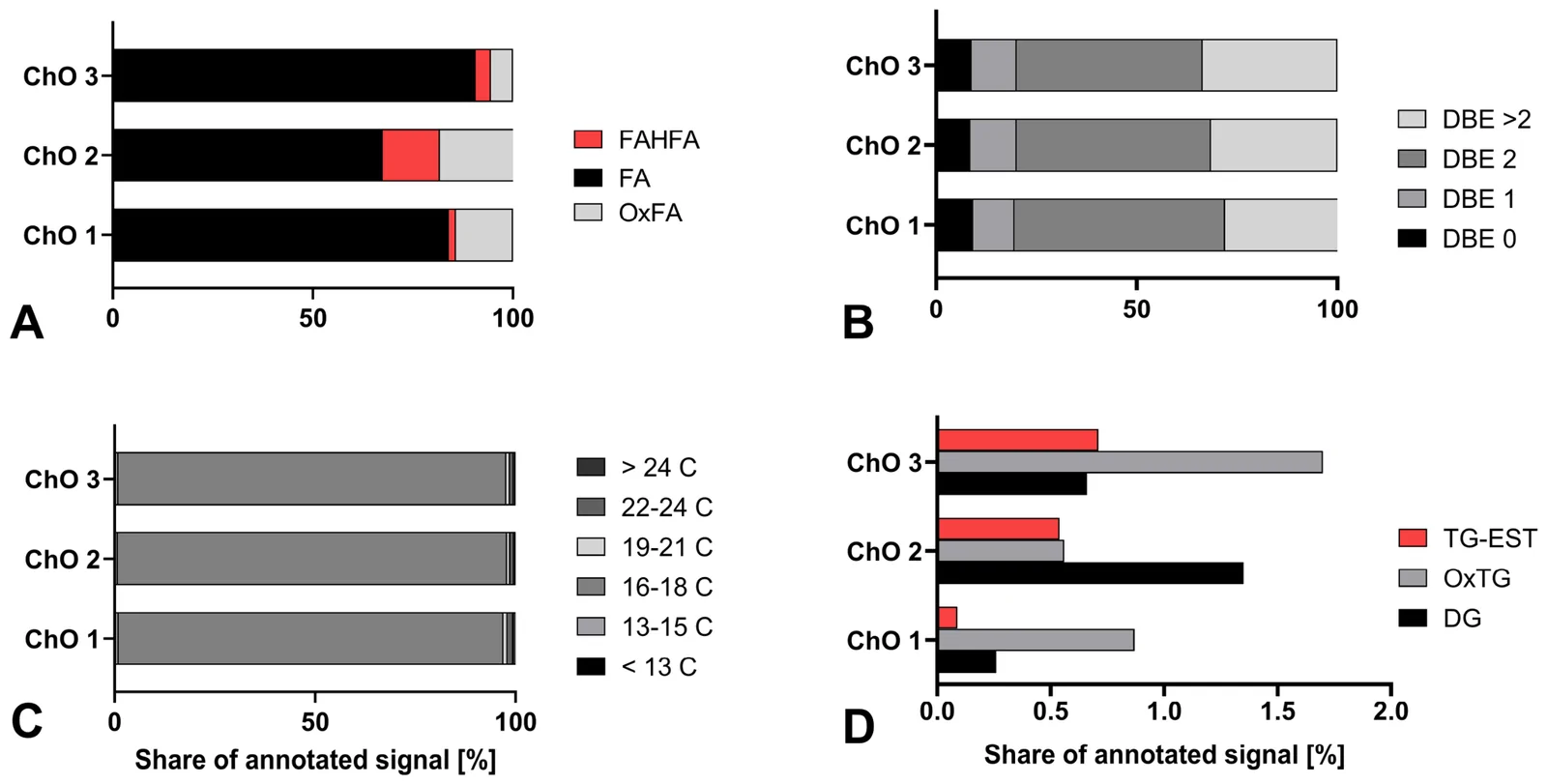
Lipid class and fatty acyl distributions of three commercial chaulmoogra oils, ChO 1, ChO 2 and ChO 3. All values are shares (%) of the annotated, normalized lipidomic signal and not concentrations or mass fractions, and each oil was injected once. (**A**) Free fatty acids (FA), oxidized free fatty acids (OxFA) and fatty acyl esters of hydroxy fatty acids (FAHFA), as shares of the annotated signal of the negative ion mode. (**B**) Fatty acyl chains classified by double bond equivalents (DBE), the sum of the C=C bonds and rings of the acyl chain, so that the cyclopentenyl 16:2 and 18:2 acyls, which carry one C=C bond and one ring, fall into the DBE 2 class. (**C**) Fatty acyl chains classified by carbon number. (**D**) Diacylglycerols (DG), oxidized triacylglycerols (OxTG) and triacylglycerol estolides (TG-EST), as shares of the annotated signal of the positive ion mode, the remainder being triacylglycerols, lysophosphatidylcholines and sterols. Panels B and C were computed at the fatty acyl level from the annotated species of both ion modes, which were pooled without cross-mode calibration (Section 2.6).

The intact triacylglycerol pool, detected in the positive ion mode, was comparable across the three oils (Table S1). The differences between the products lay in the minor classes usually associated with hydrolysis and oxidation, and these classes did not rank the three oils in one order (Figure 2A,D). In the negative ion mode, FAHFA accounted for about 14% of the annotated signal of ChO 2, compared with about 2% in ChO 1 and 4% in ChO 3, and the oxidized free fatty acids accounted for about 18% in ChO 2, 14% in ChO 1 and 5% in ChO 3. In the positive ion mode, diacylglycerols accounted for about 1.3% of the annotated signal of ChO 2, against 0.3% in ChO 1 and 0.7% in ChO 3, whereas the oxidized triacylglycerols (about 1.7%) and the triacylglycerol estolides (about 0.7%) reached their highest shares in ChO 3. The acid value was also highest in ChO 3 (Table 1). Lysophosphatidylcholines, also formed by hydrolysis, reached their highest share in ChO 2, with 0.04% of the positive-mode signal against about 0.001% in the other two. ChO 2 therefore had the highest FAHFA and diacylglycerol shares, and ChO 3 the highest acid value and oxidized triacylglycerol share. No single parameter ranked the three products consistently, so the products differ in which of these classes are elevated and not in a single degree of degradation. The decimals are retained for traceability to Tables S1 and S2 and do not imply a determined precision.

These values are shares of the annotated, normalized signal of one ion mode and not concentrations, and with one injection per sample they carry no determined uncertainty. Excluding from the negative-mode denominator the three most intense features, whose mass error indicates detector saturation (Section 2.2), left the FAHFA shares in the same order (4.6, 26.5 and 11.9% for ChO 1, ChO 2 and ChO 3). The FAHFA share of ChO 2 was about eight times that of ChO 1 and nearly four times that of ChO 3, and its diacylglycerol share about five and two times those of ChO 1 and ChO 3, respectively. In untargeted liquid chromatography coupled with mass spectrometry (LC-MS), a feature is typically retained when its relative standard deviation across repeated injections of a pooled quality control sample is below 20 or 30%, depending on the sample type [22]. The contrasts of ChO 2 with ChO 1 therefore exceed the typical variability of untargeted LC-MS, whereas twofold differences, such as the diacylglycerol share of ChO 2 against ChO 3, the FAHFA share of ChO 2 against ChO 3 without the three most intense features and the oxidized triacylglycerol share of ChO 3 against ChO 1, may lie within it. This comparison does not replace a determination of repeatability [22].

Of the classes in which ChO 2 had the highest share, the diacylglycerols have a precedent as a quality indicator. They accumulate by hydrolysis during the storage of virgin olive oil; the ratio of their 1,3- and 1,2-isomers has been proposed for assessing the genuineness of such oils of low acidity in the early stages of storage [23], and standardized methods exist for their determination [23,24,25]. The present untargeted data give the share of the class and do not resolve its isomers. A diacylglycerol carries no free carboxyl group and does not contribute to the acid value, so this class carries information that the acid value does not capture. The free fatty acid share of the negative-mode signal did not follow the acid value either, and the two are therefore reported as separate parameters. The FAHFA have no comparable precedent. Their higher share in ChO 2 is compatible with oxidative modification, since the hydroxy-acyl moiety of a FAHFA derives from a hydroxylated chain, but it does not demonstrate it in the absence of an independent measure of oxidation. FAHFA also occur natively in plant oils at trace levels, and their levels change with refining [26,27], so an origin in the raw material or in its processing cannot be excluded.

Fatty acyl chains are classified in Figure 2B by double bond equivalents (DBE), the sum of the C=C bonds and rings of the acyl chain, because the cyclopentenyl 16:2 and 18:2 acyls carry one C=C bond and one ring and are not polyunsaturated in the usual sense. In all three oils, chains with two DBE predominated. ChO 1 showed the highest proportion of these, at 52.4% against 48.4% in ChO 2 and 46.3% in ChO 3, whereas the proportion of acyls bearing more than two double bond equivalents ran in the opposite direction, at 28.0% in ChO 1, 31.5% in ChO 2 and 33.6% in ChO 3. Acyls with no and with one DBE varied little between the three, at 8.5 to 9.3% and 10.4 to 11.6%, respectively.

Comparison of fatty acyl chain lengths showed that acyls containing 16 to 18 carbon atoms predominated in all three oils, at 96.0, 97.0 and 96.7% of the total in ChO 1, ChO 2 and ChO 3 respectively (Figure 2C). The chain-length distribution was essentially identical across the three oils, and very long-chain fatty acyls (more than 24 carbons) accounted for less than 0.6% of the acyl-level signal, closely comparable between samples.

The differences in the minor lipid classes are usually associated with hydrolysis and oxidation, although for the FAHFA an origin in the raw material cannot be excluded. The stage at which they arose cannot be identified from the available information, since the suppliers provide no data on sourcing, post-harvest processing or storage, and the acquisition dates of the materials were not recorded (Section 3.1).

The solidification temperatures ranked in the reverse order of the share of acyls with more than two DBE. With three materials analysed once each, no correlation coefficient is estimable, and the concordance of rank order is reported here as an observation consistent with the known dependence of lipid phase behaviour on acyl unsaturation, not as a demonstrated relationship. The phase transition temperature of lipids is primarily determined by fatty acid chain length and degree of unsaturation [10,28]. Because the proportion of saturated acyls was similar across all three oils (about 9%), the differences in solidification temperature are unlikely to be attributable to saturation. They instead tracked the share of acyls with more than two DBE, with the oil with the highest share of these acyls (ChO 3) showing the lowest value and the oil with the lowest share (ChO 1) the highest, as more highly unsaturated acyls disrupt the regular packing of hydrocarbon chains and reduce the crystallization temperature [29]. The order is the same within the positive-mode data alone, which carry the triacylglycerols and therefore the bulk of the oil, at 25.4, 28.3 and 29.3% for ChO 1, ChO 2 and ChO 3, although ChO 2 and ChO 3 differ by only one percentage point.

The solidification points lie between refrigerated and ambient storage temperatures, so all three oils are solid at 4 °C. At 20 °C, the temperature of observation, ChO 2 and ChO 3 were fluid, whereas ChO 1, whose solidification point lies at 16 °C, was semi-solid (Table 1). Between those two temperatures, a range that includes the temperature of an unheated production area, ChO 1 may be at or below its solidification point while ChO 3 remains liquid. The behaviour of lipid phases in colloidal systems and their interaction with stratum corneum lipids has been described elsewhere [30,31], but the present data do not establish which oil is preferable for any given dosage form. Answering that question would require dedicated preformulation studies, including rheological characterization and emulsification testing. The predominance of cyclopentenyl fatty acids raises the question of a permeation-enhancing effect. Chemical penetration enhancers of every structural class, fatty acids among them, act on the stratum corneum by extraction or fluidization of the intercellular lipid lamellae [32,33]. Whether a terminal cyclopentene ring modifies that behaviour relative to a straight-chain unsaturated acyl of the same carbon number has not been examined, here or elsewhere, and the point is left as a hypothesis rather than as an inference from the present data. Three properties relevant to the use of the oil as an excipient may be affected by the differences reported here, and each is a hypothesis for preformulation work rather than a conclusion of this study. The first is the physical state of the lipid phase, discussed above. The second is chemical stability. Hydroxylated and oxidized acyl species lie on the oxidation pathway of unsaturated lipids [34]; oxidizing impurities carried by excipients are a documented concern for oxidation-sensitive active substances [35], and the European Pharmacopoeia controls the peroxide value of fixed oils alongside the acid value [7]. The third is interfacial and crystallization behaviour. Free fatty acids and diacylglycerols are more polar than triacylglycerols, and minor components of this kind modify the crystallization of fats [36], so they may influence emulsification and the physical stability of a lipid-based dosage form. Chaulmoogra oil has been employed as the lipid phase of patented multifunctional transdermal carriers, at approximately 5% of the carrier, in formulations for dapagliflozin [37] and for the local anaesthetics lidocaine and tetracaine [38]. The carriers themselves are described in the corresponding patents [39,40,41]. This is the practical reason why an identity and degradation specification for the oil is needed now rather than after a monograph appears.

### 2.5. Microbiological Activity

Despite the historical use of chaulmoogra oil against *Mycobacterium leprae*, no preparation abolished the growth of any of the three organisms at any concentration applied, and the largest reduction of the optical density at 600 nm (OD600) relative to the vehicle control observed among the test preparations was 24.8%, obtained with a methanolic extract and not with a crude oil.

The conclusion of this section rests on the broth dilution data. That format is quantitative, complete for all three organisms across eight concentrations, and the reference antibiotics reduced the optical density of every organism within it, so its sensitivity to inhibition is demonstrated rather than assumed. The two qualitative agar formats carried no positive control, so neither their sensitivity nor their detection limit is known, and the absence of a visible effect in either cannot be read as the absence of an effect. They are reported in the Supplementary Materials (Section S1, Table S4 and Figure S15) and support no conclusion.

The optical density data are given in Table S5, and their statistical description in Tables S6 and S7. In *C. albicans,* no test preparation differed from the vehicle by more than 0.08 OD600 units in the pooled comparison (Table S6). In *E. coli* and *B. cereus,* the two-way model returned *p*-values below the threshold for the concentration and interaction terms (Table S6), and these results are therefore reported at the level of individual concentrations (Table S7).

No crude oil lowered the OD600 of any organism by more than 6.3%, the largest reduction being that of ChO 2 against *C. albicans* at 62.5 µg/mL (Table S5). Against *E. coli*, the pooled value of the three oils at 250 µg/mL was 1.19 ± 0.06 (*n* = 9) against 1.17 for the vehicle, and at every concentration the pooled value of the oils was within 1% of the vehicle value or above it. The two largest positive deviations of the crude oils against *E. coli* (Table S7) are not interpreted, for the reason given below for *B. cereus*. The three methanolic extracts behaved differently. Each lowered *E. coli* OD600 at the two highest concentrations, by 20.7% at 250 µg/mL and 14.8% at 125 µg/mL for the three extracts pooled, the mean differences from the vehicle at 250 µg/mL being −0.22, −0.22 and −0.29 OD600 units, and in every extract the OD600 tended to decrease with increasing concentration (Spearman rho −0.68 to −0.78, Table S7). As stated in Section 3.7, the replicate readings came from a single culture, so these differences are reported as magnitudes and not as evidence of a reproducible biological effect. Among the crude oils, the OD600 of ChO 2 approached the vehicle value from above as the concentration increased and fell below it only at the highest concentration, by 4.7% (Table S7). The material proposed here as an excipient is the oil itself and not its methanolic extract, so the lower optical density obtained with the polar fraction does not bear on the suitability of the oil as a lipid phase.

In *B. cereus*, the OD600 of cultures exposed to the oils and extracts was higher than that of the vehicle control in 46 of the 48 preparation and concentration combinations, the largest difference being +0.48 OD600 units. The vehicle control itself gave an OD600 of 1.29 against 1.63 for the inoculated medium without vehicle, a difference of 20.4%. Two explanations cannot be separated with the present data: a suppressive effect of the dimethyl sulfoxide (DMSO) and Tween 80 vehicle on the growth of this organism, which the lipid partially offsets, and a contribution of the intrinsic turbidity of the emulsified preparations to the measured optical density. The absorbance of the vehicle itself was subtracted at the measurement stage, so the first of these would be a biological effect and not an optical one, but no blank in this experiment could remove the second. Light scattering by the emulsified preparations would add to the measured optical density and could therefore conceal a reduction of growth, but without an abiotic blank neither the direction nor the size of the optical contribution was measured, and a reduction of the order of the positive deviations recorded here could have been masked. Against this organism, the OD600 tended to increase with concentration for ChO 3 and for the three methanolic extracts (Table S7). A concentration-dependent contribution of the emulsified preparations to the measured turbidity is compatible with that pattern. One point is not. ChO 2 gave 1.778 ± 0.002 at 31.25 µg/mL against 1.304 and 1.319 at the adjacent concentrations, which is the largest positive deviation in the dataset and does not lie on any monotonic trend. It is treated here as an artefact of that plate, and no interpretation rests on it. The *B. cereus* values are therefore not interpreted as evidence of growth promotion.

The reference antibiotics reduced the optical density of every culture to which they were applied, which confirms that the readout responds to inhibition of growth. Relative to the vehicle, clotrimazole reduced the OD600 of *C. albicans* by 1.04 units, and neomycin reduced that of *E. coli* by 0.37 units and that of *B. cereus* by 0.49 units (Table S6). Against *B. cereus*, neomycin reduced the OD600 to 0.055 ± 0.010 at 62.5 µg/mL, a reduction of 96% relative to the vehicle, so growth was abolished for that combination under the definition adopted in Section 3.6.2. For *C. albicans* and *E. coli,* that endpoint was not reached within the ranges applied, with the lowest values recorded being 0.496 and 0.164, corresponding to reductions of 75 and 86% respectively. Two pairs of adjacent points in the neomycin series against *E. coli* are not in monotonic order (Table S5), the larger departure being 0.961 ± 0.073 at 31.25 µg/mL against 0.399 ± 0.003 at half that concentration, which is more readily explained by a handling error than by the dose response. The series is used here only to document that the assay detects inhibition.

Two features of the broth format are stated here because they bear on what its result can be taken to mean. The inoculum was 10^6^ colony-forming units per mL (CFU/mL), against 5 × 10^5^ CFU/mL in the reference broth dilution procedure for bacteria and a density three orders of magnitude lower for yeasts, and the yeast was grown in a rich medium rather than in a buffered synthetic one. Both features depart from the reference procedures in the direction that raises the concentration at which inhibition becomes measurable, and the reference antibiotics behaved accordingly, neomycin abolishing the growth of *B. cereus* at 62.5 µg/mL while clotrimazole and neomycin reduced the optical density of *C. albicans* and *E. coli* by 75 and 86% at the same concentration without reaching that endpoint. The format therefore documents the absence of a measurable reduction under the conditions described, and it is for this reason that no minimum inhibitory concentration is reported for the test preparations. The highest concentration tested, 250 µg/mL or 0.025% (*w*/*v*), is two orders of magnitude below the proportion of the lipid phase in the carriers referred to in Section 2.4, about 5%, so the result does not extend to the concentration at which the oil is used.

Under the broth dilution conditions used, no measurable growth inhibition of the three organisms by the crude oils was detected. The discrepancy between the historical reports and the present findings is consistent with an activity restricted to mycobacteria and mechanistically specific. Hydnocarpic acid inhibits the multiplication of *Mycobacterium intracellulare* in vitro, an activity not shared by several straight-chain fatty acids nor by dihydrochaulmoogric acid, and its inhibition is antagonized by biotin, on which basis the authors proposed that the cyclopentenyl acid acts as a structural analogue of biotin rather than through a general effect on the cell envelope [42]. Neither *M. leprae*, which cannot be cultured in vitro, nor any fungus was examined in that work, so no inference about either follows from it here.

### 2.6. Limitations

Several limitations of this study should be acknowledged. First, the analyses were performed on three commercial products, each analyzed once. This design is sufficient to show that products marketed under the same botanical name can differ in their shares of lipid classes associated with hydrolysis and oxidation, but it cannot describe the variability of the commercially available material, cannot separate between-supplier from between-batch variation, and does not permit statistical modelling of either. A survey covering several batches from a larger number of suppliers would be required for that purpose, and the framework described here is intended to support such a study rather than to replace it. The compositional differences reported here should accordingly be read as differences between commercial products as received by an end user, and not as differences attributable to an identified step of sourcing, processing or storage, for which no information was available. Second, the two qualitative agar formats carried no positive control and are reported in the Supplementary Materials only, so the microbiological result rests on the broth dilution data alone, in which the reference antibiotics reduced the optical density of every organism. The highest concentration tested in that format, 250 µg/mL, is two orders of magnitude below the proportion of the lipid phase in the carriers referred to in Section 2.4. Third, the lipidomic data were acquired as a single injection per sample without pooled quality control samples and without internal standards, so the analytical repeatability of the LC-MS measurement was not determined, no difference between the oils can be tested statistically, and the reported values are shares of normalized signal rather than concentrations. Before the FAHFA and diacylglycerol shares could serve as quality attributes, three steps would be required: targeted quantification with class-specific, stable isotope-labelled internal standards, calibration against the acid value and the peroxide value determined by the compendial methods, and measurement on a defined number of batches of known provenance and storage history. Fourth, the absence of acquisition dates and batch identifiers for the three commercial materials (Section 3.1) means that differences in pre-purchase storage history cannot be excluded. Fifth, ChO 1 was characterized by GC-MS in a previous study rather than concurrently with ChO 2 and ChO 3, and the material was no longer available for re-analysis. Sixth, the antimicrobial testing was restricted to three model organisms, which are surrogates for the compendial strains rather than the compendial strains themselves, which do not include a mould and do not represent the physiological skin microbiome, and the turbidimetric endpoint was applied to emulsified preparations. As stated in Section 3.6.2, no abiotic blank containing the test article was run, so the intrinsic light scattering of the emulsified preparations could not be removed. The vehicle control was determined in triplicate against twenty-four determinations per test preparation, which limits the precision of the reference value, and all replicate readings came from a single culture per preparation and concentration. These limitations bear on the negative result. Light scattering by the emulsified preparations could conceal a reduction of growth, and an imprecise reference value limits the size of a reduction that can be detected. The microbiological conclusion is therefore restricted to the observation that no measurable growth inhibition was detected under the broth dilution conditions used for the organisms tested. To examine the effect of chaulmoogra oil on skin commensals, extended studies using representative strains such as *Staphylococcus epidermidis*, *Cutibacterium acnes*, and *Malassezia furfur* are necessary. Seventh, the peroxide value was not determined on these oils. The acid value was, and is reported in Table 1, but the peroxide value of a fixed oil changes during storage, so a determination made now on material analyzed earlier would not be comparable with the lipidomic data reported here. The material was, in addition, exhausted. The oxidation-associated classes reported here therefore remain without a titrimetric reference. Three materials also do not permit a correlation to be tested, and the acid value and the oxidation-associated classes describe different degradation pathways, hydrolysis and oxidation respectively, so agreement between them was not expected and is not claimed. Eighth, the class-level shares shown in Figure 2 are derived from normalized signal intensities and are not corrected for differences in ionization efficiency between lipid classes, so they support the comparison of a given class between samples rather than the comparison of different classes within one sample. Ninth, the positive-mode and negative-mode data were acquired separately and were combined for the fatty acyl-level distributions of Figure 2B,C without cross-mode calibration, so the relative weight of the two ion modes conditions those distributions. Since the summed annotated signal of the negative mode differed between the samples, the rank order of the acyl classes discussed in Section 2.4 was checked within the positive mode alone and found to be the same. Tenth, the annotation returned no feature for TG 48:6, the triacylglycerol composed of three hydnocarpic acyls. Earlier work on this oil places that species second among 136 annotated triacylglycerols, at 10.17% of the triacylglycerol pool [9]. The targeted experiment of Section 3.4.2 confirmed TG 48:6 together with TG 44:6 and TG 46:6, none of which has an annotated counterpart in Table S1, and the acyl composition returned by the untargeted workflow differed from the fragmentation result in at least one further case (Figure S4). Every one of these species carries a hydnocarpic acyl. Consistent with that gap, the acyl-level distributions derived from the annotated species place 16:2 below 18:2 and 18:3, whereas the GC-MS composition places hydnocarpic acid first, so the distributions of Figure 2B,C under-represent this acyl relative to the mass composition. A second contribution acts in the same direction. Hydnocarpic acyls are also annotated in oxidized and ester-linked forms, namely two oxidized free fatty acids referred to as FA 16:2;O, seven FAHFA species carrying a 16:2;O residue, and twelve oxidized triacylglycerols containing a 16:2 chain, so part of this acyl pool is distributed across those classes rather than counted as the parent species. Finally, no preformulation work was performed, so the relationship between the compositional differences reported here and the performance of a finished dosage form remains to be established. No difference reported for the lipidomic data has a determined uncertainty, and none should be read as a statistical result. All lipidomic comparisons in this work are descriptive.

## 3. Materials and Methods

### 3.1. Reagents and Investigated Oils

Three commercial samples of cold-pressed chaulmoogra oil (*Hydnocarpus wightianus* Blume, CAS 8001-74-9, Chemical Abstracts Service, Columbus, OH, USA), obtained from three different manufacturers, were included in the study. The botanical identity of the three materials rests on the declaration of the supplier, because a commercial oil carries no voucher specimen. Independent support comes from the composition itself: the three cyclopentenyl acids characteristic of the genus were the dominant acyl class in every sample (Section 2.3). The materials were obtained through two independent procurement routes. ChO 1 (Supplier A, India) and ChO 2 (Supplier B, India) were supplied through the procurement channel of the industrial partner, whereas ChO 3 (Supplier C, India) was purchased independently through a public online retail channel. All three materials are marketed as cold-pressed oils and none is supplied against a pharmacopoeial specification. Exact acquisition dates and batch identifiers were not recorded and were not provided by the suppliers. From receipt onwards, all oils were stored under identical conditions in light-protected glass containers at 4 °C to minimize auto-oxidation prior to analysis. Liquid chromatography-mass spectrometry (LC-MS) grade methanol, acetonitrile, 2-propanol, and water, together with LC-MS grade formic acid and ammonium formate, were used for chromatographic separation and for lipidomic sample preparation. Dimethyl sulfoxide (DMSO) and the remaining reagents were of analytical grade. All solvents and reagents were purchased from Sigma-Aldrich (Merck, Darmstadt, Germany).

### 3.2. Physicochemical Characterization

The macroscopic properties (colour, consistency) were assessed visually at 20 ± 1 °C. Density was determined with a calibrated 25 mL pycnometer according to the European Pharmacopoeia general method 2.2.5. The solidification point was determined by an open-tube tilting method. Briefly, oil samples (3 mL) were cooled in a controlled ice-salt bath (−10 °C to −20 °C) with continuous temperature monitoring, and the solidification point was taken as the temperature at which the meniscus remained immobile upon 90° tilting of the tube. The parameter is reported as the solidification point and not as the freezing point of European Pharmacopoeia general chapter 2.2.18, which is determined in a jacketed apparatus and is read from the temperature plateau accompanying crystallization of the supercooled liquid [7]. The two are not interchangeable, and the value given here is method-dependent. The acid value was determined by titration according to ISO 660:2020 [12] against a reagent blank and is expressed in mg KOH per g of oil. All measurements were carried out in triplicate on a single lot of each material, in the same period as the chromatographic acquisitions, so that the values of Table 1 are contemporaneous with the lipidomic and gas chromatographic data.

### 3.3. Sample Preparation Procedures

#### 3.3.1. Preparation of Methanolic Extracts for GC-MS Analysis and Antimicrobial Assays

To evaluate the biological activity of the polar lipid fraction, methanolic extracts were prepared using the liquid–liquid extraction (LLE) protocol previously described by Schäfer et al. [18]. Methanol was selected on the basis of that earlier work, in which the cyclopentenyl fatty acids accounted for 83.5% of the fatty acid methyl ester fraction of the methanolic extract against about 80% in the crude oil, which shows that this solvent recovers the characteristic acids of chaulmoogra oil without fractionating them. The extent to which that holds differed between the materials, as documented in Section 2.3. Briefly, 10.0 g of each oil sample was subjected to sequential extraction with methanol (15 mL per cycle) at 30 °C, the temperature at which the oils were fully liquefied. Each extraction cycle consisted of vigorous shaking for 5 min, followed by a 5-min settling period to ensure complete phase separation. The upper methanolic layer was then carefully decanted using a glass pipette. This procedure was repeated six times to ensure exhaustive extraction of polar constituents, yielding a pooled methanolic extract for each oil sample.

To eliminate any potential solvent toxicity that could interfere with microbiological assays, the combined methanolic extracts were evaporated to dryness under reduced pressure using a rotary evaporator (40 °C). The resulting residues were weighed and reconstituted in dimethyl sulfoxide (DMSO) supplemented with 0.5% (*w*/*v*) Tween 80 to achieve the required stock concentrations for antimicrobial testing. Crude oil samples (without extraction) were directly emulsified in the same DMSO/Tween 80 vehicle to ensure homogeneous dispersion and enable comparative evaluation.

#### 3.3.2. Sample Preparation for UPLC-MS/MS (Lipidomics)

For untargeted lipid profiling, a direct dilution method was employed to preserve the native lipid composition. Prior to sample preparation, the oil samples were removed from refrigerated storage and warmed to 45 °C until complete liquefaction. A 10 µL aliquot of each liquefied oil was diluted with 2.00 mL of an isopropanol, acetonitrile and water mixture (IPA:ACN:H_2_O, 4:3:1, *v*/*v*/*v*), corresponding to an oil-to-diluent ratio of 1:200 (*v*/*v*) and to a final oil concentration of approximately 0.50% (*v*/*v*). The samples were vortex-mixed for 5 min and centrifuged at 16,900× *g* for 5 min. The resulting supernatants were transferred to LC-MS vials, and 2 µL aliquots were injected into the chromatographic system. No internal standards were added to the samples.

#### 3.3.3. Derivatization for GC-MS (Fatty Acid Methyl Esters)

To determine the fatty acid profile, triacylglycerols and free fatty acids were converted into fatty acid methyl esters (FAMEs) by acid-catalyzed methanolysis, following the protocol described previously by Schäfer et al. [18]. Briefly, 0.5 g of oil was dissolved in 12.5 mL of methanol, and a 0.5 mL portion of this solution was transferred into a glass tube fitted with a polytetrafluoroethylene (PTFE) stopper. The methanolic extracts were evaporated to dryness under reduced pressure, and the residues were reconstituted in 12.5 mL of methanol, from which 0.5 mL portions were taken. Each portion was combined with 0.5 mL of methanol and 30 µL of concentrated sulfuric acid (98%) and heated in an oil bath at 100 °C for 1.5 h with regular stirring. After cooling, 1 mL of water, 2 mL of hexane and 1 mL of brine were added and the mixture was shaken manually for 1 min, after which the lower methanol and water layer was discarded. The hexane layer was washed successively with 1 mL of brine, 1 mL of saturated potassium bicarbonate solution and a further 1 mL of brine, the lower layer being discarded after each step. Finally, 1 g of anhydrous magnesium sulfate was added, the sample was left to stand for 5 min and filtered through a hydrophobic PTFE syringe filter (0.22 µm) into a glass vial before GC-MS analysis.

### 3.4. UPLC-QTOF-MS/MS Lipidomic Profiling

Analyses were performed on a Waters ACQUITY UPLC I-Class system coupled to a Waters SYNAPT G2-Si quadrupole time-of-flight (QTOF) mass spectrometer (both Waters Corp., Milford, MA, USA) equipped with an electrospray ionization (ESI) source. Separations employed an ACQUITY UPLC CSH C18 column (2.1 × 100 mm, 1.7 µm) at 45 °C with a flow rate of 0.300 mL/min and an injection volume of 2.0 µL. Mobile phase A consisted of acetonitrile-water (3:2, *v*/*v*) with 5 mM ammonium formate, and mobile phase B consisted of 2-propanol and acetonitrile (9:1, *v*/*v*) with 5 mM ammonium formate. The gradient program is detailed in Table 3. Data were acquired in both positive and negative ESI modes in separate injections. Source parameters are summarized in Table 4. Spectra were acquired over the mass range *m*/*z* 50 to 1400. Data were acquired in data-independent mode (MSE) with a low collision energy of 10 V and a high collision energy of 30 V. Mass accuracy was maintained by LockSpray with leucine enkephalin, monitored at *m*/*z* 556.2771 in positive mode and at *m*/*z* 554.2615 in negative mode. Representative base peak intensity (BPI) chromatograms acquired in the negative ion mode are presented in Figure S1, and total ion current (TIC) chromatograms acquired in the positive ion mode are presented in Figure S2, for all three oil samples.

#### 3.4.1. UPLC-MS/MS Data Processing and Lipid Identification

Raw data acquisition was controlled by MassLynx software (version 4.2, Waters Corp., Milford, MA, USA), while data processing, including peak picking, alignment, and deconvolution, was performed using MS-DIAL (version 4.9.221218). Signal intensities were normalized using the modified total ion current (mTIC) procedure implemented in MS-DIAL [43,44]. The values reported in Tables S1 and S2 are therefore relative normalized signal intensities, used for descriptive comparison between the three oils. No cross-mode calibration was applied, so each mode was normalized within itself. The column sums of Tables S1 and S2 are not equal between samples, so the class-level values reported in Section 2.4 are shares within one ion mode and within one sample and do not support the comparison of absolute amounts between samples. The fatty acyl-level distributions reported in Section 2.4 draw on the annotated species of both modes. Class-level values are therefore shares of the annotated, normalized signal of one ion mode, and neither class-level nor fatty acyl-level values are concentrations or mass fractions. Because the analytical repeatability was not determined, every comparison between the oils is descriptive.

Lipid annotation was based on accurate mass measurements and rule-based interpretation of MS/MS fragmentation patterns, supported by searches against lipidomics databases. Annotation was not filtered against a fixed mass accuracy threshold. Spectral matching was performed against the libraries built into MS-DIAL. Annotations are reported in the shorthand notation of LIPID MAPS [45]. Some species-level names in Tables S1 and S2 keep the format of the annotation software: the number of additional oxygen atoms may precede the letter (;2O for ;O2), and the names of FAHFA count the ester carbonyl as a double bond equivalent and its oxygen as an additional oxygen atom, so that FAHFA 34:1;2O corresponds to FAHFA 34:0;O in this notation. Species assigned to the higher confidence level in Tables S1 and S2 are reported at the molecular species level: the fatty acyl composition is supported by MS/MS fragments, the underscore separator indicates that the sn-positions were not assigned, and neither the double bond positions nor the position of the cyclopentene ring were determined. In FAHFA names, the slash separates the esterified fatty acid from the hydroxy fatty acid. For species assigned to the lower level, the composition given in Table S1 is the most probable of the possible compositions, and only the sum composition is regarded as established. For several oxidized fatty acids the annotation software proposed a hydroxyl position, given as (2OH) in Table S2. That position was not confirmed experimentally, and these species are referred to in the text as FA x:y;O. The annotations used here do not distinguish cyclopentenyl fatty acids characteristic of chaulmoogra oil from straight-chain isomers with the same molecular formula. For example, gorlic acid and α-linolenic acid are both represented as FA 18:3 in this shorthand notation. Therefore, assignment of these signals to cyclopentenyl fatty acid-containing lipids was additionally supported by the GC-MS data rather than by LC-MS alone. Throughout the text, the second number in lipid shorthand annotations denotes the nominal degree of unsaturation inferred from elemental composition. For cyclopentenyl fatty acid-containing species, this value may include contributions from ring structures and should not be interpreted solely as the number of acyclic C=C double bonds. Representative MS/MS fragmentation spectra illustrating the identification strategy for triacylglycerols and diacylglycerols containing cyclopentenyl fatty acid residues are provided in Figures S3–S9. To ensure high-confidence identification, the retention behaviour of detected species was verified against the Equivalent Carbon Number (ECN) model, which confirmed the regularity of elution order relative to the carbon chain length and degree of unsaturation [46,47]. One restriction has to be stated. The retention rules underlying that model were established for straight-chain acyls, and a cyclopentenyl ring is not chromatographically equivalent to the double bond with which it shares a molecular formula. In the most complete intact-triacylglycerol analysis of chaulmoogra oil, triacylglycerols carrying cyclopentenyl acyls eluted after their straight-chain isobars rather than at the same position. The shift is small. Four isobaric pairs of cyclopentenyl and straight-chain triacylglycerols differed by 0.5 to 1.3 min within a triacylglycerol elution window of about 50 min [9] (Section 3.3.2, Table 3 and Figure 3 of that work). The behaviour is attributed to the position of the ring, since a cyclopentene ring sits at the end of the acyl chain and leaves most of that chain free to interact with the stationary phase, whereas a cyclopropene ring in mid-chain disrupts the interaction and reverses the order [9] (Section 3.3.2 of that work). The ECN model was therefore used as a consistency check on elution order and not as a criterion of identity. The identification workflow is illustrated in the Supplementary Materials for two representative species. For a triacylglycerol composed of chaulmoogric, hydnocarpic and gorlic acid residues, Figure S4 shows the precursor ions, Figure S5 the acyl-loss fragments that define the remaining chains, and Figure S6 the acylium fragments together with the ions at *m*/*z* 67 and 81 that are diagnostic of the cyclopentenyl ring. Figures S7–S9 present the same three levels of evidence for a diacylglycerol composed of hydnocarpic and gorlic acid residues. These fragmentation experiments belong to the targeted acquisition described in Section 3.4.2, which was carried out on a methanolic extract and not on the diluted oils of Section 3.3.2. They are presented as structural support for the annotation and as an illustration of its logic, and not as a source of the intensities reported here. The class-level and fatty acyl-level distributions were computed from the normalized intensities using the LipidOne version 2.1 [48,49], which aggregates the annotated species by lipid class and by the composition of their individual acyl chains.

The lipidomic dataset was acquired as a single injection per sample. Pooled quality control samples were not prepared, and no internal standards were used, so neither analytical repeatability nor signal drift across the sequence could be quantified. The controls that were in place were the acquisition of all three oils within one analytical sequence per ion mode under identical instrumental conditions, mTIC normalization of the intensities, reporting of the average signal-to-noise ratio for every annotated species in Tables S1 and S2, confirmation of identity by MS/MS fragmentation, and independent verification of the fatty acyl composition by GC-MS with retention index validation on two stationary phases and a certified 37-component fatty acid methyl ester (FAME) reference material. The LC-MS data are therefore used to compare lipid class profiles and not to support quantitative claims.

#### 3.4.2. Targeted MS/MS Confirmation of Triacylglycerol and Diacylglycerol Structures

The structural assignments illustrated in Figures S3–S9 originate from a separate experiment carried out on the methanolic extract of ChO 2, prepared as described in Section 3.3.1. The profiling of the three oils was untargeted, whereas this experiment selected individual precursor ions for fragmentation, so the two acquisitions are described separately. The chromatographic system, the column and the gradient program of Table 3 were those of Section 3.4, with a column temperature of 45 °C, an autosampler temperature of 22 °C and an injection volume of 1 µL. Mobile phase A was acetonitrile and water (3:2, *v*/*v*) and mobile phase B was 2-propanol and acetonitrile (9:1, *v*/*v*), both containing 10 mM ammonium formate and 0.1% formic acid. Data were acquired in the positive ion mode only, with a capillary voltage of 0.5 kV, a source temperature of 125 °C, a sampling cone of 40 V, a source offset of 40 V, a desolvation temperature of 450 °C, a cone gas flow of 80 L h^−1^, a desolvation gas flow of 950 L h^−1^ and a nebulizer gas pressure of 6.0 bar. Twelve triacylglycerol precursors and nine diacylglycerol precursors were selected as ammonium adducts, each within a defined retention window, and were fragmented in the transfer cell with a collision energy ramp of 15 to 45 eV. For the triacylglycerols, the presence of every species was established from three precursor ions, [M+NH_4_]^+^, [M+H]^+^ and [M−H_2_O+H]^+^, and its acyl composition from the fragment ions [M−RCO_2_]^+^, [RCO+74]^+^, [RCO]^+^ and [RC]^+^, together with the cyclopentenyl ions. For the diacylglycerols the sodium adduct [M+Na]^+^ was recorded alongside those three precursor ions, and the acyl composition was established from the [RCO+74]^+^, [RCO]^+^ and [RC]^+^ ions; the neutral loss of one fatty acid from the protonated precursor yielded in this class the same ions as [RCO+74]^+^. Figure S3 shows the extracted mass chromatograms of the twelve triacylglycerol precursors. Of the nine diacylglycerols, only DG 34:5, composed of hydnocarpic and gorlic acyls, is illustrated in Figures S7–S9. Because this experiment used a different sample, a different mobile phase additive and different source settings, its signal intensities are not comparable with those of Tables S1 and S2 and are not used for any quantitative statement in this work.

### 3.5. GC-MS Analysis and Compound Identification

GC-MS analyses were performed using a Hewlett-Packard 6890 series gas chromatograph equipped with a 5973 mass selective detector (both Hewlett-Packard, now Agilent Technologies, Santa Clara, CA, USA). A dual-column strategy was employed for profiling of fatty acid methyl esters. Two capillary columns were used: a non-polar ZB-5HT (5% diphenyl/95% dimethylpolysiloxane, 60 m × 0.25 mm i.d., 0.25 µm film thickness) and a polar ZB-Wax Plus (30 m × 0.32 mm i.d., 0.25 µm film thickness), both supplied by Phenomenex Inc. (Torrance, CA, USA). Helium was used as the carrier gas at a constant flow rate of 1.0 mL/min (ZB-5HT) and 1.5 mL/min (ZB-Wax Plus). The injection volume was 1 µL with a split ratio of 20:1 at an injector temperature of 250 °C. The GC-MS interface temperature was set to 300 °C (ZB-5HT) or 250 °C (ZB-Wax Plus), and the ion source was maintained at 230 °C. Mass spectra were acquired in scan mode over a range of 30–550 *m*/*z*. The oven temperature program for ZB-5HT was as follows: initial temperature 60 °C, ramped at 20 °C/min to 170 °C, then at 5 °C/min to 250 °C, and finally at 20 °C/min to 300 °C with an isothermal hold for 8 min. For ZB-Wax Plus: initial temperature 50 °C, ramped at 15 °C/min to 200 °C, then at 5 °C/min to 250 °C with an isothermal hold for 7 min.

Chromatographic data were processed using MSD ChemStation software (version E.02.02, Agilent Technologies, Santa Clara, CA, USA) coupled with the MS Search program (version 2.7) of the National Institute of Standards and Technology (NIST) and with the Automated Mass Spectral Deconvolution and Identification System (AMDIS, NIST, Gaithersburg, MD, USA) for spectral deconvolution. Data alignment and retention index verification were performed with the Excel-based tool Retentify [50]. Peak assignments for the resulting chromatograms, listed in order of elution on the ZB-5HT column, are given in Table S3.

Compound identification followed a rigorous multi-criteria validation protocol: 1. Spectral Matching: Experimental mass spectra were compared against NIST 11 and NIST 14 libraries. 2. Retention Index Validation: Linear Retention Indices (LRIs) were calculated relative to a homologous series of *n*-alkanes (Sigma-Aldrich, Merck, Darmstadt, Germany) according to the Van Den Dool and Kratz equation. The experimental linear retention indices (LRIs) were verified against reference data from Babushok [51] and the NIST Chemistry WebBook [52]. Neither source tabulates a reference LRI for the cyclopentenyl fatty acid methyl esters, and the LRIref values reported here for methyl hydnocarpate, methyl gorlate and methyl chaulmoograte therefore originate from Schäfer et al. [18]. 3. Standard Confirmation: Whenever applicable, identifications were unequivocally confirmed using a certified reference material of fatty acid methyl esters (Supelco 37-Component FAME Mix, Sigma-Aldrich, Merck, Darmstadt, Germany).

Quantification: A semi-quantitative approach was applied. The relative abundance of individual analytes was calculated as the ratio of the specific peak area to the total chromatographic peak area.

### 3.6. Antimicrobial Activity Evaluation

#### 3.6.1. Microbial Strains and Culture Conditions

Chaulmoogra oil is evaluated here as a lipid excipient and not as an active ingredient, so the microbiological work tested whether the oil inhibits microbial growth. An excipient intended for dermal application should neither inhibit nor promote microbial growth, because inhibitory activity would confound the interpretation of antimicrobial efficacy testing of the finished product and would interfere with the design of the preservative system, whereas a material that supports growth would burden it. The work reported here addresses the first of these two properties. A turbidimetric endpoint applied to emulsified preparations cannot separate an increase in cell number from an increase in the intrinsic light scattering of the preparation, so the absence of growth promotion is not established by these data and would require enumeration of viable cells. The panel was chosen to span three of the four morphological groups represented among the organisms used in compendial microbiological quality testing of non-sterile pharmaceutical preparations and substances for pharmaceutical use, namely a Gram-negative bacterium, a Gram-positive spore-forming bacterium and a yeast (general chapters 5.1.4, 2.6.12 and 2.6.13) [7]. The antimicrobial potential was evaluated against three reference strains: Gram-negative *Escherichia coli* (CCM 5172) and Gram-positive *Bacillus cereus* (CCM 2010) obtained from the Czech Collection of Microorganisms (Masaryk University, Brno, Czech Republic), and the yeast *C. albicans* (LOCK 001) sourced from the Institute of Fermentation Technology and Microbiology (Lodz University of Technology, Łódź, Poland). These are surrogates for the compendial organisms rather than the compendial organisms themselves. *Bacillus cereus* stands here for the Gram-positive spore-forming group, which those chapters represent by *Bacillus subtilis*, and the *C. albicans* isolate used is not the strain the chapters specify. The fourth group, the moulds, represented compendially by *Aspergillus brasiliensis*, was not examined.

Prior to testing, bacteria were cultured on Nutrient Agar (NA) at 34 °C, while *C. albicans* was cultured on yeast extract peptone dextrose (YPD) medium at 25 °C. For all assays, 24-h cultures were diluted with the appropriate sterile medium to achieve a standardized inoculum density of approximately 10^6^ colony-forming units (CFU)/mL.

#### 3.6.2. Broth Dilution Assay

Quantitative assessment was performed using the serial dilution method in liquid medium. Test articles were prepared in DMSO with 0.5% Tween 80 vehicle as concentrated stocks and two-fold serially diluted. A 20 µL aliquot of each dilution was added to 2.00 mL of the standardized inoculum, giving eight final concentrations of 250, 125, 62.5, 31.25, 15.63, 7.81, 3.91 and 1.95 µg/mL, each determined in triplicate. Because the dilution series was prepared in the vehicle and a constant volume was added at every step, the final concentration of dimethyl sulfoxide was approximately 1% (*v*/*v*) at every concentration of the series, identical to that of the vehicle control.

The inoculum density was 10^6^ CFU/mL. Clotrimazole served as the reference antifungal for *C. albicans* and neomycin as the reference antibacterial for *E. coli* and *B. cereus*, both at six final concentrations of 62.5 to 1.95 µg/mL. Two controls were run in parallel, namely the DMSO with 0.5% Tween 80 vehicle with inoculum and the inoculated medium without vehicle. After 24 h of static incubation, growth was quantified by measuring optical density at 600 nm (OD600) using a RayLeigh UV-2601 spectrophotometer (Beijing Beifen-Ruili Analytical Instrument Co., Ltd., Beijing, China) against a reference of uninoculated medium containing the DMSO and Tween 80 vehicle, so that the absorbance of the medium and of the solvent was subtracted from every reading. Abolition of growth was taken as a reduction of OD600 by more than 95% relative to the vehicle control, which corresponds to the absence of visible turbidity. No minimum inhibitory concentration is reported for the test preparations, for the reason given in Section 2.5. No abiotic blank containing the test article itself was run, so the absorbance of the medium and of the vehicle was removed, but the intrinsic light scattering of the emulsified oil could not be.

Two qualitative agar formats, well diffusion and agar incorporation, were also applied. Neither carried a positive control, and both are described together with their results in the Supplementary Materials (Section S1, Table S4 and Figure S15).

### 3.7. Statistical Analysis

Antimicrobial data are expressed as mean ± standard deviation (SD) of three replicate readings (*n* = 3) of a single culture per preparation and concentration, as stated in the footnote to Table S5. Independent biological cultures were not prepared, so the replicate readings describe the dispersion of the measurement and not biological variability. The *p*-values given in Tables S6 and S7 are reported on that basis, as a description of the dispersion between replicate readings, and are not interpreted as evidence of reproducible biological effects. The microbiological conclusion rests on the absence of a measurable reduction of growth by the crude oils up to 250 µg/mL in the broth dilution format and not on any between-group comparison. For each strain, the data were first analyzed by two-way analysis of variance (ANOVA) with preparation and concentration as fixed factors. Where either the concentration term or the preparation by concentration interaction reached significance, each preparation and concentration combination was compared against the vehicle control separately by Dunnett’s two-sided test, and the monotonicity of the response was assessed by Spearman rank correlation between OD600 and concentration. A pooled one-way analysis across concentrations, followed by Dunnett’s test against the vehicle control, is reported for every strain in Table S6 as a descriptive summary of the overall exposure, irrespective of the outcome of the two-way model. It is not used to exclude effects at individual concentrations, which are reported separately in Table S7. Normality of residuals was assessed by the Shapiro–Wilk test and homogeneity of variance by Levene’s test. Where residuals departed from normality, the parametric analysis was repeated as a Kruskal–Wallis test with Dunn’s post hoc comparison (Holm-adjusted). Effect sizes are given in Table S6 as η^2^ for the omnibus ANOVA and as the mean difference with 95% confidence interval (CI) and Hedges’ g for the antibiotic-versus-vehicle comparisons. A two-sided *p* < 0.05 was used as the threshold in Tables S6 and S7. Table S6 gives exact *p*-values, and Table S7 marks the comparisons below that threshold. Analyses were performed using Statistica 13.3 (TIBCO Software Inc., Palo Alto, CA, USA).

## 4. Conclusions

Three commercial chaulmoogra oils with an essentially identical fatty acid composition, dominated by cyclopentenyl acids, differed in the minor lipid classes usually associated with hydrolysis and oxidation. The differences did not run in one direction. ChO 2 had the highest shares of FAHFA and diacylglycerols, whereas ChO 3 had the highest acid value and the highest share of oxidized triacylglycerols. All class-level lipidomic values are shares of the annotated, normalized signal of one ion mode, obtained from a single injection per sample, and every comparison between the oils is descriptive.

Two conclusions follow for the three products examined. A specification based on the fatty acid composition alone would not separate them, and the acid value, 1.9 to 2.8 mg KOH/g, separated them over a narrow range.

The FAHFA and diacylglycerol shares are therefore proposed as candidate attributes associated with hydrolysis and oxidation, not as markers ready for a specification, and for the FAHFA an origin in the raw material has first to be excluded. Before either could serve as a quality attribute, it would require targeted quantification with class-specific internal standards, calibration against the acid value and the peroxide value determined by the compendial methods, and a survey of several batches from a larger number of suppliers.

Under the broth dilution conditions used, no measurable growth inhibition of *Escherichia coli*, *Bacillus cereus* or *Candida albicans* by the crude oils was detected up to 250 µg/mL. Compatibility with the skin microbiota, permeation behaviour and performance in a finished dosage form were not examined and do not follow from these data.

## Figures and Tables

**Table 1:** Physicochemical parameters of chaulmoogra oils from different suppliers.

Oil Sample	Colour	Consistency	Density (g/mL)	Solidification Point (°C)	Acid Value (mg KOH/g)
ChO 1	Golden (medium yellow)	Semi-solid	0.92 ± 0.002	16 ± 0.3	1.9 ± 0.10
ChO 2	Cream, light yellow	Liquid	0.94 ± 0.002	15 ± 0.2	2.4 ± 0.15
ChO 3	Very light yellow	Liquid	0.93 ± 0.002	10 ± 0.4	2.8 ± 0.20

**Table 2:** Fatty acid methyl ester profiles of ChO 2 and ChO 3 oils and their methanolic extracts determined by GC-MS.

No.	Compound	Shorthand	CAS	RTavg	LRIexp	LRIref	ChO 2 [%]	ChO 3 [%]	ChO 2 MeOH Extract [%]	ChO 3 MeOH Extract [%]
1	methyl hexadec-9-enoate #	16:1	10030-74-7	19.2	1912	1912	4.2	4.2	4.1	5.0
2	methyl palmitate (hexadecanoic acid, methyl ester) #	16:0	112-39-0	19.6	1931	1929	6.6	6.7	7.1	6.7
3	methyl hydnocarpate (methyl 11-(2-cyclopenten-1-yl)undecanoate)	16:2 (cyc)	459-67-6	20.8	1996	1990	30.0	32.1	22.5	24.3
4	methyl 14-methylhexadecanoate (hexadecanoic acid, 14-methyl-, methyl ester) *	17:0 (br)	2490-49-5	20.9	2003	1996	0.3	0.2	-	0.3
5	methyl dihydrohydnocarpate (cyclopentaneundecanoic acid, methyl ester)	16:1 (cyc)	25779-85-5	20.9	2006	nd	-	0.4	-	-
6	methyl linoleate (9,12-octadecadienoic acid, methyl ester) #	18:2	112-63-0	22.5	2104	2103	1.9	-	-	2.3
7	methyl octadec-11-enoate (11-octadecenoic acid, methyl ester) #	18:1	52380-33-3	22.5	2105	nd	-	-	3.0	2.3
8	methyl oleate (9-octadecenoic acid, methyl ester) #	18:1	2462-84-2	22.6	2110	2107	3.3	2.1	4.7	3.2
9	15-octadecenoic acid, methyl ester *	18:1	10411-39-9	22.6	2116	nd	-	-	-	2.5
10	methyl stearate #	18:0	112-61-8	22.9	2133	2130	0.7	0.5	1.1	0.7
11	methyl gorlate (6-tridecenoic acid, 13-(2-cyclopenten-1-yl)-, methyl ester)	18:3 (cyc)	502-31-8	23.5	2182	2177	18.9	20.8	14.1	15.8
12	methyl chaulmoograte (13-(2-cyclopenten-1-yl)tridecanoic acid, methyl ester, (S)-)	18:2 (cyc)	24828-59-9	23.8	2204	2197	23.2	23.8	19.1	17.5

Legend: Values are percentages of the total chromatographic peak area. # identification confirmed by the 37-FAME reference material. * preliminary identification, with no linear retention index (LRI) value to confirm the MS spectrum match, or with LRI validated but spectral similarity below 80%. nd, no data available. The shorthand notation follows the carbon number to double bond convention. cyc denotes a cyclopentenyl fatty acid, and br denotes a branched-chain fatty acid. The cyclopentenyl 16:2, 18:2, and 18:3 acyls are isomeric with the straight-chain hexadecadienoic, linoleic, and α-linolenic acids, respectively and are distinguished here by their retention indices. A hyphen denotes a compound that was sought and not detected in the given sample. Methyl linolenate (α-linolenic acid, 18:3, CAS 301-00-8) was sought in all four samples and was not detected. The compounds listed account for 89.1%, 90.8%, 75.7% and 80.6% of the total chromatographic peak area of ChO 2, ChO 3, the ChO 2 methanolic extract and the ChO 3 methanolic extract, respectively, the balance corresponding to peaks that did not satisfy the identification criteria. Abbreviations: CAS, Chemical Abstracts Service registry number. FAME, fatty acid methyl ester. GC-MS, gas chromatography coupled with mass spectrometry. LRIexp, experimental linear retention index. LRIref, reference linear retention index. MeOH, methanol. RTavg, average retention time.

**Table 3:** Mobile phase gradient program.

Time (min)	Flow (mL min^−1^)	%A	%B	Curve
0.00	0.300	68.0	32.0	Initial
1.00	0.300	68.0	32.0	6
15.00	0.300	45.0	55.0	6
48.00	0.300	15.0	85.0	6
49.00	0.300	0.0	100.0	6
49.50	0.300	68.0	32.0	6
55.00	0.300	68.0	32.0	6

**Table 4:** ESI source parameters for positive and negative ion modes.

Parameter	Setting	Setting
Polarity	ESI (+)	ESI (−)
Capillary	3.00 kV	2.50 kV
Source temperature	140 °C	140 °C
Sampling cone	40 V	39 V
Source offset	30 V	30 V
Cone gas flow	50 L h^−1^	50 L h^−1^
Desolvation gas flow	850 L h^−1^	850 L h^−1^
Desolvation temperature	450 °C	450 °C
Nebulizer gas	3.5 bar	4.0 bar

## Data Availability

All data supporting the reported results are contained within the manuscript and its Supplementary Materials. Other data are available from the corresponding author on request.

## Supplementary Materials

The following supporting information can be downloaded at: https://www.mdpi.com/article/10.3390/molecules31203582/s1, Figure S1: Base peak intensity chromatograms of the three oils, ESI negative mode; Figure S2: Total ion current chromatograms of the three oils, ESI positive mode; Table S1: Lipid species identified by UPLC-QTOF-MS/MS in ESI positive mode; Table S2: Free fatty acids, fatty acyl esters of hydroxy fatty acids and oxidized free fatty acids identified in ESI negative mode; Figure S3: Extracted mass chromatograms of the triacylglycerol precursor ions selected for MS/MS; Figure S4: MS/MS spectrum of the chaulmoogric, hydnocarpic and gorlic triacylglycerol, precursor ions; Figure S5: The same triacylglycerol, acyl-loss fragments; Figure S6: The same triacylglycerol, acylium fragments and the cyclopentenyl markers at *m*/*z* 67 and 81; Figure S7: MS/MS spectrum of the hydnocarpic and gorlic diacylglycerol, precursor ions; Figure S8: The same diacylglycerol, acyl-loss fragments; Figure S9: The same diacylglycerol, acylium fragments; Figure S10: GC-MS chromatogram of ChO 2 on the ZB-5HT column; Figure S11: GC-MS chromatogram of ChO 3 on the ZB-5HT column; Figure S12: GC-MS chromatogram of the ChO 2 methanolic extract on the ZB-5HT column; Figure S13: GC-MS chromatogram of the ChO 3 methanolic extract on the ZB-5HT column; Table S3: Peak assignments for Figures S10–S13; Figure S14: Heatmap of 50 annotated lipid species across the three samples; Section S1: Qualitative agar formats, well diffusion and agar incorporation; Table S4: Representative photographs of the agar well diffusion assay; Table S5: Mean optical density of cultures exposed to the oils, their methanolic extracts and the reference standards; Table S6: Pooled one-way analysis of variance with Dunnett’s test against the vehicle control; Table S7: Concentration-resolved effect on the optical density of *Escherichia coli and Bacillus cereus* cultures; Figure S15: Agar incorporation assay against *Candida albicans*, vehicle control and the three oils.

## Abbreviations

The following abbreviations are used in this manuscript:

AAHFA	Acyl alpha-hydroxy fatty acid
ACN	Acetonitrile
AMDIS	Automated Mass Spectral Deconvolution and Identification System
ANOVA	Analysis of variance
BPI	Base peak intensity
CAS	Chemical Abstracts Service
CCM	Czech Collection of Microorganisms
CFU	Colony-forming unit
ChO	Chaulmoogra oil
CI	Confidence interval
DBE	Double bond equivalent
DG	Diacylglycerol
DMSO	Dimethyl sulfoxide
ECN	Equivalent carbon number
ESI	Electrospray ionization
FA	Fatty acid, used as the lipid class label for free fatty acids in negative ion mode
FAHFA	Fatty acyl ester of hydroxy fatty acid
FAME	Fatty acid methyl ester
GC-MS	Gas chromatography coupled with mass spectrometry
IPA	2-Propanol
LC-MS	Liquid chromatography coupled with mass spectrometry
LLE	Liquid–liquid extraction
LOCK	Pure culture collection of industrial microorganisms (LOCK 105), Lodz University of Technology
LPC	Lysophosphatidylcholine
LRI	Linear retention index
LRIexp, LRIref	Experimental and reference linear retention index
MeOH	Methanol
MS/MS	Tandem mass spectrometry
MSE	Data-independent acquisition with alternating low and elevated collision energy
mTIC	Modified total ion current normalization
NA	Nutrient agar
NEG	Negative ion mode
NIST	National Institute of Standards and Technology
OD600	Optical density at 600 nm
OxFA	Oxidized free fatty acid
OxTG	Oxidized triacylglycerol
Ph. Eur.	European Pharmacopoeia
POS	Positive ion mode
PTFE	Polytetrafluoroethylene
QTOF	Quadrupole time-of-flight
RT	Retention time
RTavg	Average retention time
SD	Standard deviation
ST	Sterol
TG	Triacylglycerol
TG-EST	Triacylglycerol estolide
TIC	Total ion current
UPLC	Ultra-performance liquid chromatography
YPD	Yeast extract peptone dextrose medium
