# Supplementary material for: Lipidomic Profiling of Commercial Hydnocarpus wightianus Oil by UPLC-QTOF-MS/MS and GC-MS: Degradation-Associated Lipid Classes as Candidate Quality Attributes for a Non-Pharmacopoeial Excipient
Source: Molecules. 2026 Oct 9;31(20):3582. doi: 10.3390/molecules31203582 (PMC13707860; doi:10.3390/molecules31203582)
Supplement: Supplementary file 1 [file molecules-31-03582-s001.zip › molecules-4564763-supplementary.pdf]

Supplementary material

Lipidomic Profiling of Commercial Hydnocarpus wightianus Oil by UPLC-QTOF-MS/MS and GC-MS: Degradation-Associated Lipid Classes as Candidate Quality Attributes for a Non-Pharmacopoeial Excipient

Radosław Balwierz[1], Natalia Schäfer[1], Patryk Kuropka[2], Jerzy Wiśniewski[2], Dorota Wieczorek[1], Paweł Biernat[3,4], Jacek Lipok[1]

1- Institute of Chemistry, University of Opole, 48 Oleska St., 45-052 Opole, Poland
2- Faculty of Chemistry, Wrocław University of Science and Technology, 4/6 Norwida St., 50-373 Wrocław, Poland
3- Department of Drug Forms Technology, Faculty of Pharmacy, Wrocław Medical University, 211 Borowska St., 50-556 Wrocław, Poland
4- Biotts S.A., 44c Wrocławska St., 55-040 Bielany Wrocławskie, Poland

Corresponding author
Radosław Balwierz, PhD; email: radoslaw.balwierz@uni.opole.pl

Table of Contents

Figure S1. Base peak intensity (BPI) chromatograms of Hydnocarpus wightianus oil samples acquired by UPLC-QTOF-MS in ESI negative mode. From top to bottom: ChO 1, ChO 2, and ChO 3. Major peaks correspond to free fatty acids identified in Table S2. The label in the upper left corner of each chromatogram is an internal instrument acquisition identifier and has no analytical significance. 6
Figure S2. Total ion current (TIC) chromatograms of Hydnocarpus wightianus oil samples acquired by UPLC-QTOF-MS in ESI positive mode. From top to bottom: ChO 3, ChO 2, and ChO 1. Dominant peaks in the 35 to 50 min range correspond to triacylglycerols (TG) identified in Table S1. The label in the upper left corner of each chromatogram is an internal instrument acquisition identifier and has no analytical significance. 7
Table S1. Lipid species identified in *Hydnocarpus wightianus* oil by UPLC-QTOF-MS/MS in ESI positive mode. Columns include retention time (RT), lipid nomenclature, class, molecular formula, measured and reference m/z, adduct form, identification confidence level, average signal-to-noise ratio, and normalized signal intensities for three commercial oil samples (ChO 1 to ChO 3). Lipid classes: DG, diacylglycerol; LPC, lysophosphatidylcholine; OxTG, oxidized triacylglycerol; ST, sterol; TG, triacylglycerol; TG-EST, triacylglycerol estolide. The Lipid identification level column reports the confidence of the structural assignment. Good confidence, confirmed fatty acyl composition denotes species for which the fatty acyl chains were confirmed by characteristic MS/MS product ions, corresponding to the molecular species level. Medium confidence, possible other fatty acyl composition denotes species for which the total carbon number and the number of double bond equivalents are established but, because of the quality of the MS/MS spectrum or its chimerism, the exact acyl chain combination is not unambiguous, so alternative acyl compositions cannot be excluded, corresponding to the species level. Signal intensities were normalized in MS-DIAL using the mTIC procedure and

are reported as relative normalized values. No internal standards were added to the samples and no absolute quantification was performed. The table lists the annotated subset of the detected features, so the column sums are not equal between samples. The values therefore support the comparison of a given species or class between samples and not the comparison of absolute amounts. Thirty-six sum compositions are annotated at more than one retention time, twenty-six of them within the triacylglycerol class. In three of these the features span about 5 min and, summed over the three oils, the latest carries 15 to 75 times the intensity of the earliest: TG 54:6 (37.77, 41.52 and 42.79 min), TG 54:7 (36.00 and 41.15 min) and TG 54:9 (32.51, 35.59, 35.86 and 37.57 min). Within the TG 54:6 set the earliest and the latest feature carry the same acyl assignment while the intermediate feature at 41.52 min is annotated differently, so only two of the three share an assignment. The pattern is not general. TG 52:8, the most intense triacylglycerol of the dataset, is annotated at 35.41, 37.14 and 37.67 min with the same acyl composition throughout and with the middle feature dominant. A separate behaviour is seen for TG 50:2, which returns three features within 0.70 min that carry three different acyl assignments and differ 32-fold in intensity, and which is therefore more readily explained as three distinct molecular species of the same sum composition than as one species split across retention times. None of these sets is assigned in the main text. 8

Table S2. Free fatty acids (FA), fatty acyl esters of hydroxy fatty acids (FAHFA), and oxidized free fatty acids (OxFA) identified in Hydnocarpus wightianus oil by UPLC-QTOF-MS in ESI negative mode. Column descriptions as in Table S1. Six species within the FAHFA class carry the prefix AAHFA in their shorthand name, as returned by the annotation software. AAHFA denotes an acyl alpha-hydroxy fatty acid, that is, a fatty acid esterified to the hydroxyl group at C-2 of a hydroxy fatty acid (Yasuda et al., iScience 2020, 23, 101841, https://doi.org/10.1016/j.isci.2020.101841). Eleven oxidized fatty acids carry the suffix (2OH), the hydroxyl position proposed by the annotation software. Neither position was confirmed experimentally, and the oxidized fatty acids are referred to in the main text as FA x:y;O. The six AAHFA species are counted within the FAHFA class throughout this work. Signal intensities are relative normalized values obtained by mTIC normalization in MS-DIAL, as for Table S1. Species marked in red with a dagger are the three most intense features of the negative ion mode, for which the mass error exceeds 10 ppm (+27.1, +16.8 and +10.7 ppm), which is the behaviour expected when the detector response approaches saturation. All other features fall within 6.4 ppm........ 17

Figure S3. Extracted mass chromatograms for the triacylglycerol precursor ions selected for the MS/MS experiment, acquired for the methanolic extract. Each trace corresponds to one MS/MS function and is labelled with the m/z of the selected [M+NH4]+ precursor ion. The precursors correspond to the following triacylglycerols, assigned by accurate mass against the annotations of Table S1: m/z 896.766, TG 54:6 (18:2_18:2_18:2); m/z 894.755, TG 54:7 (18:2_18:2_18:3); m/z 892.732, TG 54:8 (18:2_18:3_18:3); m/z 890.725, TG 54:9 (18:3_18:3_18:3); m/z 868.745, TG 52:6 (16:2_18:2_18:2); m/z 866.727, TG 52:7 (16:1_18:3_18:3); m/z 864.711, TG 52:8 (16:2_18:3_18:3); m/z 840.714, TG 50:6 (16:1_16:2_18:3); m/z 838.690, TG 50:7 (16:2_16:2_18:3); m/z 812.681, TG 48:6; m/z 784.647, TG 46:6; and m/z 756.613, TG 44:6. All assignments agree with the reference masses to within 8 mDa. The three lowest-mass precursors are given at the sum-composition level only,

because the corresponding features were not annotated in the oil samples of Table S1, the MS/MS experiment having been performed on the methanolic extract. 24

Figure S4. Fragment of the MS/MS mass spectrum for triacylglycerol consisting of chaulmoogric, hydnocarpic and gorlic acid residues. Visible signals coming from the precursor ions [M+NH4]+, [M+H]+ and [M-H2O+H]+. The precursor ions appear at m/z 866.72 ([M+NH4]+), 849.70 ([M+H]+), and 831.69 ([M-H2O+H]+), calculated for C55H92O6 (TG 52:7). The precursor at m/z 866.72 is annotated in Table S1 as TG 52:7 with the acyl composition 16:1_18:3_18:3, whereas the fragmentation shown in Figures S4 to S6 corresponds to the composition 16:2_18:2_18:3, that is chaulmoogric, hydnocarpic and gorlic acid residues. Both correspond to the same sum composition and the two isomers were not resolved under the conditions used. 25

Figure S5. Fragment of the MS/MS mass spectrum for triacylglycerol consisting of chaulmoogric, hydnocarpic and gorlic acid residues. Signals from [M-RCO2]+ fragment ions are visible. Each [M-RCO2]+ ion corresponds to the neutral loss of one fatty acid from the protonated precursor, with the two remaining acyls retained. Loss of hydnocarpic acid (16:2) yields m/z 597.49, loss of chaulmoogric acid (18:2) yields m/z 569.46, and loss of gorlic acid (18:3) yields m/z 571.47. 26

Figure S6. Fragment of the MS/MS mass spectrum for triacylglycerol consisting of chaulmoogric, hydnocarpic and gorlic acid residues. Visible signals coming from fragment ions [RCO+74]+, [RCO]+, [RC]+. Additionally, characteristic signals of 67 and 81 m/z are visible, indicating the presence of a cyclopentenyl ring in the structure of fatty acid residues. The [RCO]+ acylium ions appear at m/z 235.21, 263.24, and 261.22 for hydnocarpic (16:2), chaulmoogric (18:2), and gorlic (18:3) acids respectively. The corresponding [RCO+74]+ ions appear at m/z 309.24, 337.27, and 335.26, and the [RC]+ ions, formed by loss of water from the corresponding acylium ion, at m/z 217.20, 245.23, and 243.21. The signals at m/z 67 (C5H7+) and 81 (C6H9+) are diagnostic of the cyclopentenyl ring. 27

Figure S7. Fragment of the MS/MS mass spectrum for a diacylglycerol consisting of hydnocarpic and gorlic acid residues. Visible signals coming from precursor ions [M+Na]+, [M+NH4]+, [M+H]+ and [M-H2O+H]+. The precursor ions appear at m/z 609.45 ([M+Na]+), 604.49 ([M+NH4]+), 587.47 ([M+H]+), and 569.46 ([M-H2O+H]+), calculated for C37H62O5 (DG 34:5). 28

Figure S8. Fragment of the MS/MS mass spectrum for a diacylglycerol consisting of hydnocarpic and gorlic acid residues. Signals from fragment ions [RCO+74]+ and low-intensity signals from fragment ions [M-RCO2]+ are visible. For this diacylglycerol, neutral loss of one fatty acid from the protonated precursor yields the monoacylglycerol-type ion of the remaining acyl, so the [RCO+74]+ and [M-RCO2]+ labels describe the same fragments: m/z 309.24, retaining hydnocarpic acid (16:2) and equivalent to the loss of gorlic acid, and m/z 335.26, retaining gorlic acid (18:3) and equivalent to the loss of hydnocarpic acid. 29

Figure S9. Fragment of the MS/MS mass spectrum for a diacylglycerol consisting of hydnocarpic and gorlic acid residues. Visible signals coming from fragment ions [RCO]+, [RC]+. The [RCO]+ acylium ions appear at m/z 235.21 (hydnocarpic, 16:2) and 261.22 (gorlic, 18:3), and the [RC]+ ions, formed by loss of water from the corresponding acylium ion, at m/z 217.20 and 243.21 respectively. 30

Figure S10. GC-MS chromatogram of the ChO 2 oil sample acquired on the ZB-5HT column. Identified fatty acid methyl esters are listed in Table 2 of the main text, and the numbered peak assignments are given in Table S3. 31
Figure S11. GC-MS chromatogram of the ChO 3 oil sample acquired on the ZB-5HT column. Identified fatty acid methyl esters are listed in Table 2 of the main text, and the numbered peak assignments are given in Table S3. 32
Figure S12. GC-MS chromatogram of the ChO 2 methanolic extract sample acquired on the ZB-5HT column. Identified fatty acid methyl esters are listed in Table 2 of the main text, and the numbered peak assignments are given in Table S3. 33
Figure S13. GC-MS chromatogram of the ChO 3 methanolic extract sample acquired on the ZB-5HT column. Identified fatty acid methyl esters are listed in Table 2 of the main text, and the numbered peak assignments are given in Table S3. 34
Table S3. Peak assignments for Figures S10 to S13. Peaks are listed in order of elution on the ZB-5HT column and numbered as in Table 2. Not all compounds are present in every sample, and their percentages of the total peak area are given in Table 2. Where two compounds share the same retention time to one decimal place, the order follows the experimental linear retention index reported in Table 2. The peaks at 22.5 min (numbers 6 and 7) and at 22.6 min (numbers 8 and 9) elute closely and may partially overlap............ 35
Figure S14. Heatmap of 50 annotated lipid species in three Hydnocarpus wightianus oil samples, comprising free fatty acids, oxidized free fatty acids, fatty acyl esters of hydroxy fatty acids and triacylglycerols. The colour scale represents the z-score computed within each sample, that is, the intensity of each species standardized against the 50 species shown for that sample to a mean of zero and a standard deviation of one, ranging from white (low) to black (high). The map therefore shows the standing of each species within the annotated signal of each sample, and it does not compare absolute intensities between samples. Because species of both ion modes enter one standardization, the standing of negative-mode species (FA, FAHFA, OxFA) against positive-mode species (TG) reflects their different ionization and not their relative amounts. No clustering was applied. All rows are labelled with the corresponding lipid species. 37
Section S1. Qualitative agar formats, well diffusion and agar incorporation 38
Table S4. Representative photographs of the agar well diffusion assay. (A) ChO 1 on an Escherichia coli lawn. (B) ChO 3 on a Bacillus cereus lawn. (C) ChO 1 on a Candida albicans lawn. (D) Methanolic extracts on Candida albicans lawns, from left to right ChO 3, ChO 1 and ChO 2. Wells were loaded with 20 μL of the test article at the concentrations given in Section S1.1, the four concentrations being distributed over separate plates carrying one to three wells each. No well-defined zone of inhibition was observed on any plate. The lawn was fully confluent for B. cereus but not for C. albicans, and for E. coli a single plate was retained, so the assay is reported as a qualitative screen. 40
Table S5. Mean optical density (OD600) of cultures exposed to the investigated oils (ChO 1 to ChO 3), their methanolic extracts, and reference standards against the selected strains. 41
Table S6. Effect of *Hydnocarpus wightianus* oils and their methanolic extracts on the optical density (OD_{600}) of *Candida albicans*, *Escherichia coli* and *Bacillus cereus* cultures,

assessed by one-way ANOVA with Dunnett's two-sided multiple-comparison test against the vehicle control... 43
Table S7. Concentration-resolved effect of Hydnocarpus wightianus oils and their methanolic extracts on the optical density (OD600) of Escherichia coli and Bacillus cereus cultures. Values are the percentage change of OD600 relative to the DMSO with 0.5% Tween 80 vehicle control. Each preparation and concentration combination (n = 3) was compared against the vehicle control (n = 3) by Dunnett's two-sided test, and comparisons significant at p < 0.05 are marked with a dagger and shown in bold. Because the replicate readings come from a single culture, the marked comparisons describe the dispersion between readings and are not evidence of reproducible biological effects. Concentrations are final concentrations in the culture, expressed in µg/mL. Spearman rho is the rank correlation between OD600 and concentration over all twenty-four determinations per preparation, and it is therefore not reproducible from the means listed in Table S5. Candida albicans is not included, because for that strain neither the concentration term nor the preparation by concentration interaction reached significance, and its results are given in the pooled analysis of Table S6. Dunnett's test uses the error variance pooled across all preparation and concentration combinations, so a group whose own replicates are widely scattered can still be flagged. This applies to ChO 3 at 125 µg/mL against E. coli, where the three replicates gave 1.425 ± 0.401. Percentage deviations were computed from the unrounded means and may differ by up to 0.1 percentage point from values recalculated using the rounded means listed in Table S5... 45
Figure S15. Agar incorporation assay against Candida albicans. Top left, vehicle control plate carrying the emulsifier without the lipid phase. Top right, ChO 1. Bottom left, ChO 2. Bottom right, ChO 3. All plates were prepared, inoculated and incubated in the same run and carry a single oil loading, the exact value of which was not recorded. The plates bear no visible identification, and the assignment above follows the position recorded at the time of photography. Colony density on the oil-containing plates is visually indistinguishable from the control. The endpoint is a visual comparison, no counts were made, and no positive control was incorporated into this format, so the comparison establishes only that none of the three oils produced near-complete inhibition at this loading.. 47

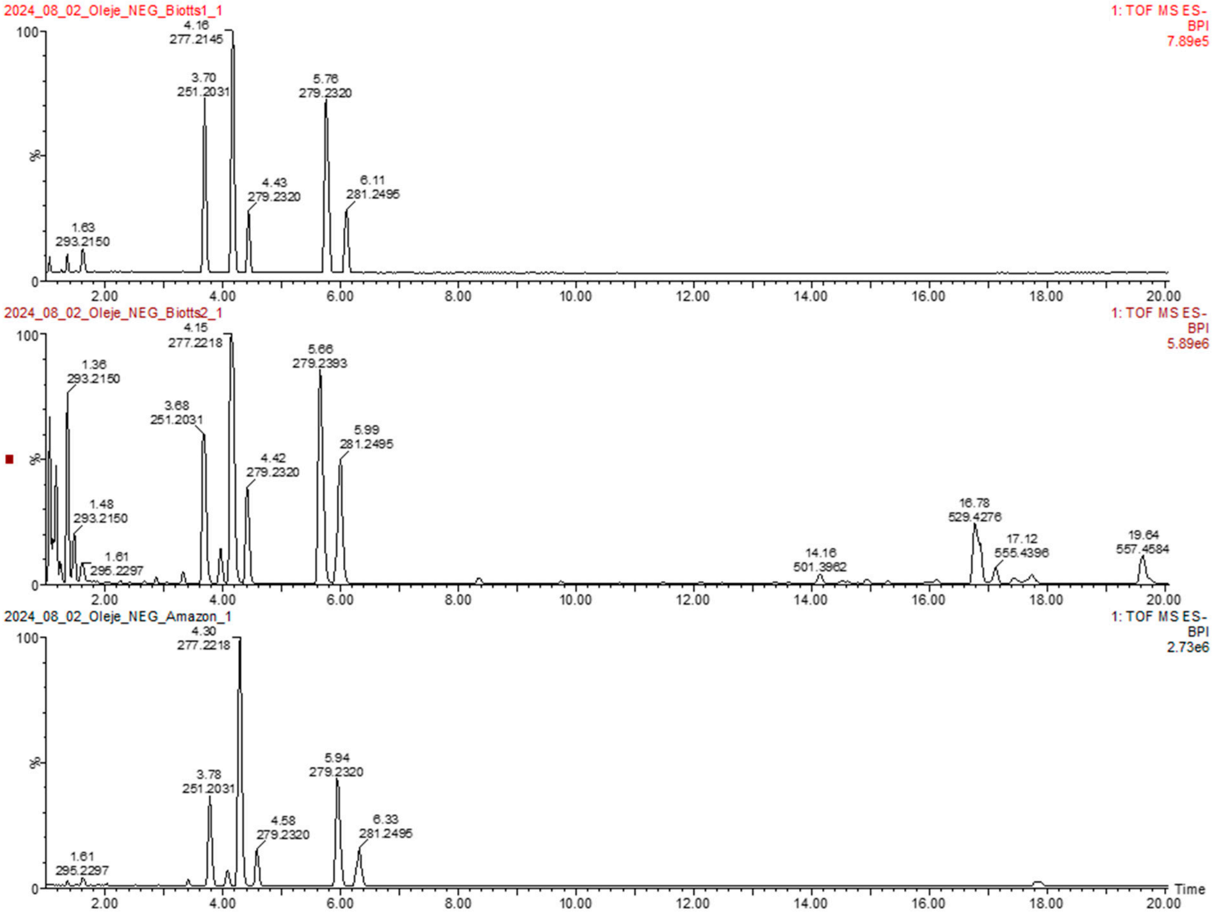

Figure S1. Base peak intensity (BPI) chromatograms of Hydnocarpus wightianus oil samples acquired by UPLC-QTOF-MS in ESI negative mode. From top to bottom: ChO 1, ChO 2, and ChO 3. Major peaks correspond to free fatty acids identified in Table S2. The label in the upper left corner of each chromatogram is an internal instrument acquisition identifier and has no analytical significance.

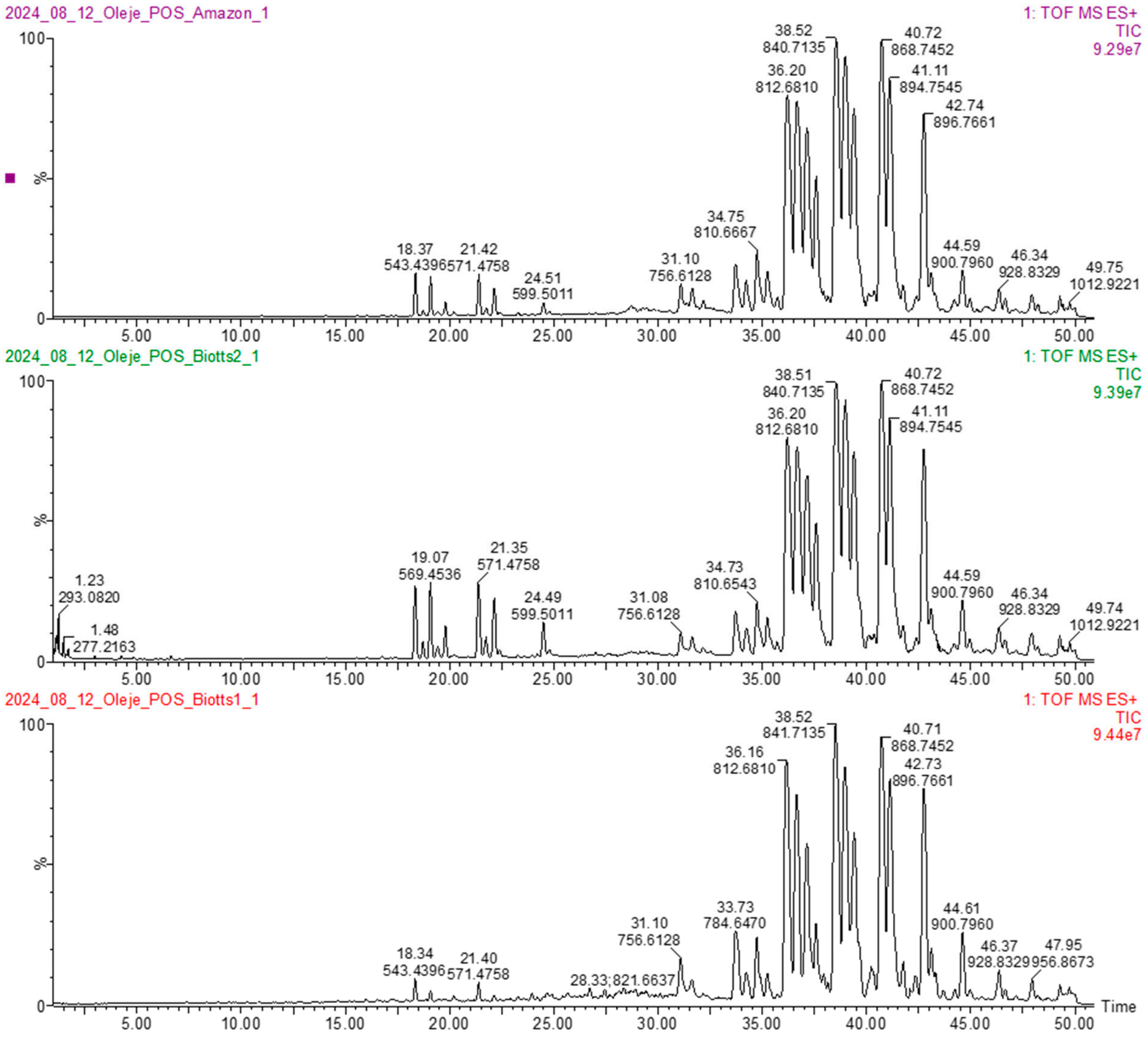

Figure S2. Total ion current (TIC) chromatograms of Hydnocarpus wightianus oil samples acquired by UPLC-QTOF-MS in ESI positive mode. From top to bottom: ChO 3, ChO 2, and ChO 1. Dominant peaks in the 35 to 50 min range correspond to triacylglycerols (TG) identified in Table S1. The label in the upper left corner of each chromatogram is an internal instrument acquisition identifier and has no analytical significance.

Table S1. Lipid species identified in *Hydnocarpus wightianus* oil by UPLC-QTOF-MS/MS in ESI positive mode. Columns include retention time (RT), lipid nomenclature, class, molecular formula, measured and reference m/z, adduct form, identification confidence level, average signal-to-noise ratio, and normalized signal intensities for three commercial oil samples (ChO 1 to ChO 3). Lipid classes: DG, diacylglycerol; LPC, lysophosphatidylcholine; OxTG, oxidized triacylglycerol; ST, sterol; TG, triacylglycerol; TG-EST, triacylglycerol estolide. The Lipid identification level column reports the confidence of the structural assignment. Good confidence, confirmed fatty acyl composition denotes species for which the fatty acyl chains were confirmed by characteristic MS/MS product ions, corresponding to the molecular species level. Medium confidence, possible other fatty acyl composition denotes species for which the total carbon number and the number of double bond equivalents are established but, because of the quality of the MS/MS spectrum or its chimerism, the exact acyl chain combination is not unambiguous, so alternative acyl compositions cannot be excluded, corresponding to the species level. Signal intensities were normalized in MS-DIAL using the mTIC procedure and are reported as relative normalized values. No internal standards were added to the samples and no absolute quantification was performed. The table lists the annotated subset of the detected features, so the column sums are not equal between samples. The values therefore support the comparison of a given species or class between samples and not the comparison of absolute amounts. Thirty-six sum compositions are annotated at more than one retention time, twenty-six of them within the triacylglycerol class. In three of these the features span about 5 min and, summed over the three oils, the latest carries 15 to 75 times the intensity of the earliest: TG 54:6 (37.77, 41.52 and 42.79 min), TG 54:7 (36.00 and 41.15 min) and TG 54:9 (32.51, 35.59, 35.86 and 37.57 min). Within the TG 54:6 set the earliest and the latest feature carry the same acyl assignment while the intermediate feature at 41.52 min is annotated differently, so only two of the three share an assignment. The pattern is not general. TG 52:8, the most intense triacylglycerol of the dataset, is annotated at 35.41, 37.14 and 37.67 min with the same acyl composition throughout and with the middle feature dominant. A separate behaviour is seen for TG 50:2, which returns three features within 0.70 min that carry three different acyl assignments and differ 32-fold in intensity, and which is therefore more readily explained as three distinct molecular species of the same sum composition than as one species split across retention times. None of these sets is assigned in the main text.

RT (min)	Name of Lipid	Lipid class	Formula	Measured m/z	Reference m/z	Adduct form	Lipid identification level	Average signal to noise ratio	Signal intensity CHO 1	Signal intensity CHO 2	Signal intensity CHO 3
16.76	DG 32:5|DG 16:2_16:3	DG	C35H58O5	576.4623	576.4623	[M+NH4]+	Good confidence - confirmed fatty acyls composition	22.64	1.94947E-06	1.04988E-05	4.79489E-06
17.38	DG 34:5|DG 16:2_18:3	DG	C37H62O5	604.4930	604.4936	[M+NH4]+	Good confidence - confirmed fatty acyls composition	71.59	4.28597E-06	1.60396E-05	6.18703E-06
18.33	DG 32:4|DG 16:2_16:2	DG	C35H60O5	578.4798	578.4779	[M+NH4]+	Good confidence - confirmed fatty acyls composition	4037.79	0.000167578	0.000432492	0.000286866
18.71	DG 32:3|DG 16:1_16:2	DG	C35H62O5	580.4938	580.4936	[M+NH4]+	Good confidence - confirmed fatty acyls composition	1659.64	4.80376E-07	0.000106061	3.72605E-05
19.07	DG 34:5|DG 16:2_18:3	DG	C37H62O5	604.4942	604.4936	[M+NH4]+	Good confidence - confirmed fatty acyls composition	2040.5	0.000139272	0.000716351	0.000435403
19.44	DG 34:4|DG 16:1_18:3	DG	C37H64O5	606.5098	606.5092	[M+NH4]+	Good confidence - confirmed fatty acyls composition	2125.35	2.61687E-05	0.000129556	4.33917E-05
19.46	DG 36:6|DG 18:3_18:3	DG	C39H64O5	630.5073	630.5092	[M+NH4]+	Good confidence - confirmed fatty acyls composition	501	4.57347E-06	3.98897E-05	1.45358E-05
19.80	DG 36:6|DG 18:3_18:3	DG	C39H64O5	630.5096	630.5092	[M+NH4]+	Good confidence - confirmed fatty acyls composition	6141.25	2.78919E-05	0.000589436	0.000255456
21.37	DG 34:4|DG 16:2_18:2	DG	C37H64O5	606.5104	606.5092	[M+NH4]+	Good confidence - confirmed fatty acyls composition	6882.97	0.000155693	0.0005433	0.000317443
21.70	DG 34:3|DG 18:1_16:2	DG	C37H66O5	608.5260	608.5249	[M+NH4]+	Good confidence - confirmed fatty acyls composition	2164.43	1.29572E-05	0.000147314	5.22485E-05
22.14	DG 36:5|DG 18:2_18:3	DG	C39H66O5	632.5255	632.5249	[M+NH4]+	Good confidence - confirmed fatty acyls composition	3405.14	9.28521E-05	0.000683871	0.000365322
22.39	DG 34:2|DG 16:0_18:2	DG	C37H68O5	610.5407	610.5405	[M+NH4]+	Medium confidence - possible other fatty acyl composition	516.43	8.40045E-06	2.48888E-05	1.45172E-05
22.39	DG 36:4|DG 18:2_18:2	DG	C39H68O5	634.5406	634.5405	[M+NH4]+	Medium confidence - possible other fatty acyl composition	1570.6	2.4111E-05	9.33282E-05	4.80809E-05
22.66	DG 36:3|DG 18:1_18:2	DG	C39H70O5	636.5543	636.5562	[M+NH4]+	Good confidence - confirmed fatty acyls composition	987.39	3.95437E-05	4.06017E-05	2.0279E-05
24.47	DG 36:4|DG 18:1_18:3	DG	C39H68O5	634.5410	634.5405	[M+NH4]+	Medium confidence - possible other fatty acyl composition	2898.22	3.97391E-05	0.000253756	9.9253E-05
24.48	DG 34:2|DG 16:0_18:2	DG	C37H68O5	610.5400	610.5405	[M+NH4]+	Medium confidence - possible other fatty acyl composition	844.82	2.87172E-06	3.14706E-05	1.34791E-05
24.75	DG 36:3|DG 18:1_18:2	DG	C39H70O5	636.5563	636.5562	[M+NH4]+	Medium confidence - possible other fatty acyl composition	901.93	2.47425E-05	4.21833E-05	1.87799E-05
24.76	DG 34:1|DG 16:0_18:1	DG	C37H70O5	612.5575	612.5562	[M+NH4]+	Medium confidence - possible other fatty acyl composition	381.46	6.53918E-06	1.47669E-05	7.733E-06
1.94	LPC 18:3	LPC	C26H48NO7P	518.3253	518.3241	[M+H]+	Good confidence - confirmed fatty acyls composition	188.81	1.00061E-07	1.23472E-05	4.04799E-08

2.05	LPC 18:2	LPC	C26H50NO7P	520.3405	520.3398	[M+H]+	Good confidence - confirmed fatty acyls composition	212.76	8.2472E-07	2.61259E-05	6.81914E-07
2.60	LPC 16:0	LPC	C24H50NO7P	496.3404	496.3398	[M+H]+	Good confidence - confirmed fatty acyls composition	140.61	2.29425E-07	1.70911E-05	6.24309E-08
2.60	LPC 18:2	LPC	C26H50NO7P	520.3402	520.3398	[M+H]+	Good confidence - confirmed fatty acyls composition	95.96	9.08706E-07	1.34084E-05	6.64277E-07
2.82	LPC 18:1	LPC	C26H52NO7P	522.3565	522.3554	[M+H]+	Good confidence - confirmed fatty acyls composition	412.63	1.43695E-06	4.41834E-05	4.93777E-07
15.18	TG 48:8;O2|TG 16:2_16:3_16:3;O2	OxTG	C51H82O8	840.6334	840.6348	[M+NH4]+	Medium confidence - possible other fatty acyl composition	98.33	0.000112871	2.73761E-06	1.49122E-07
17.95	TG 49:7;O2|TG 16:2_17:2_16:3;O2	OxTG	C52H86O8	856.6661	856.6661	[M+NH4]+	Medium confidence - possible other fatty acyl composition	98.42	2.32934E-05	2.15867E-07	3.74221E-08
18.67	TG 50:7;2O|TG 16:2_16:2_18:3;O2	OxTG	C53H88O8	870.6791	870.6817	[M+NH4]+	Medium confidence - possible other fatty acyl composition	24.07	1.97593E-05	4.05409E-07	7.70747E-08
24.20	TG 48:7;O|TG 16:2_16:2_16:3;O	OxTG	C51H84O7	826.6543	826.6555	[M+NH4]+	Medium confidence - possible other fatty acyl composition	44.9	1.73928E-05	1.46449E-05	5.22941E-06
24.91	TG 48:7;O|TG 16:2_16:2_16:3;O	OxTG	C51H84O7	826.6547	826.6555	[M+NH4]+	Medium confidence - possible other fatty acyl composition	597.89	0.000448557	8.85672E-05	3.54689E-05
25.44	TG 48:7;O|TG 16:2_16:3_16:2;O	OxTG	C51H84O7	826.6536	826.6555	[M+NH4]+	Good confidence - confirmed fatty acyls composition	197.07	6.29783E-05	4.01461E-06	5.62938E-06
28.72	TG 48:6;O|TG 16:2_16:2_16:2;O	OxTG	C51H86O7	828.6701	828.6712	[M+NH4]+	Good confidence - confirmed fatty acyls composition	13224.7	0.001194565	0.000548903	0.001084646
28.83	TG 48:7;O|TG 16:2_16:3_16:2;O	OxTG	C51H84O7	826.6545	826.6555	[M+NH4]+	Medium confidence - possible other fatty acyl composition	244.85	6.12539E-05	9.74982E-05	0.000112504
29.82	TG 48:5;O|TG 16:2_16:2_16:1;O	OxTG	C51H88O7	830.6860	830.6868	[M+NH4]+	Good confidence - confirmed fatty acyls composition	4972.46	6.0545E-06	5.87407E-05	0.000307976
31.10	TG 50:7;O|TG 16:2_16:2_18:3;O	OxTG	C53H88O7	854.6850	854.6868	[M+NH4]+	Medium confidence - possible other fatty acyl composition	3825.45	0.000395786	0.000308828	0.001481016
31.63	TG 52:8;O|TG 16:2_18:3_18:3;O	OxTG	C55H90O7	880.7009	880.7025	[M+NH4]+	Medium confidence - possible other fatty acyl composition	2210.11	0.000200993	0.000288377	0.001238151
32.17	TG 54:9;O|TG 18:3_18:3_18:3;O	OxTG	C57H92O7	906.7170	906.7181	[M+NH4]+	Good confidence - confirmed fatty acyls composition	4164.72	4.42037E-05	0.000130696	0.000579163
32.43	TG 50:5;O|TG 16:2_18:2_16:1;O	OxTG	C53H92O7	858.7169	858.7181	[M+NH4]+	Good confidence - confirmed fatty acyls composition	3738.66	5.96026E-05	9.07943E-05	0.000404393
14.88	ST 28:2;O	ST	C28H46O	399.3618	399.3621	[M+H]+	Good confidence - confirmed fatty acyls composition	695.65	2.18253E-05	5.53914E-05	2.42262E-05
16.04	ST 29:2;O	ST	C29H48O	413.3786	413.3778	[M+H]+	Good confidence - confirmed fatty acyls composition	4927.35	0.000104927	0.000307084	0.000145779
17.20	ST 29:1;O	ST	C29H50O	397.3836	397.3829	[M+H-H2O]+	Good confidence - confirmed fatty acyls composition	128.27	4.48018E-05	1.91551E-05	2.59833E-05
41.96	ST 29:1;O	ST	C29H50O	397.3828	397.3829	[M+H-H2O]+	Good confidence - confirmed fatty acyls composition	307.33	4.04668E-05	6.95897E-05	5.06107E-05

44.13	ST 29:1;O	ST	C29H50O	397.3830	397.3829	[M+H-H2O]+	Good confidence - confirmed fatty acyls composition	158.86	3.10626E-05	5.8447E-05	3.04078E-05
32.51	TG 54:9|TG 18:3_18:3_18:3	TG	C57H92O6	890.7236	890.7232	[M+NH4]+	Good confidence - confirmed fatty acyls composition	925.99	3.37498E-05	0.000516972	1.24787E-05
33.24	TG 48:8|TG 16:2_16:3_16:3	TG	C51H82O6	808.6443	808.6450	[M+NH4]+	Good confidence - confirmed fatty acyls composition	1330.77	0.000104203	9.45135E-05	0.000129037
34.57	TG 45:4|TG 13:0_16:2_16:2	TG	C48H84O6	774.6602	774.6606	[M+NH4]+	Medium confidence - possible other fatty acyl composition	8119.45	0.000348058	0.000104491	0.000108511
34.75	TG 48:7|TG 16:2_16:2_16:3	TG	C51H84O6	810.6616	810.6606	[M+NH4]+	Good confidence - confirmed fatty acyls composition	183851.38	0.01002703	0.007365092	0.008940502
34.93	TG 50:7|TG 16:2_16:2_18:3	TG	C53H88O6	838.6901	838.6919	[M+NH4]+	Good confidence - confirmed fatty acyls composition	11992.18	0.0027652	0.001985463	0.00186725
35.09	TG 50:6|TG 16:1_16:2_18:3	TG	C53H90O6	840.7052	840.7076	[M+NH4]+	Medium confidence - possible other fatty acyl composition	5188.46	0.000468518	0.000820677	0.000857947
35.24	TG 50:8|TG 16:2_16:3_18:3	TG	C53H86O6	836.6755	836.6763	[M+NH4]+	Good confidence - confirmed fatty acyls composition	98748.93	0.00450801	0.005144253	0.006170956
35.41	TG 52:8|TG 16:2_18:3_18:3	TG	C55H90O6	864.7063	864.7076	[M+NH4]+	Good confidence - confirmed fatty acyls composition	11928.03	0.001100149	0.001459254	0.001377194
35.53	TG 52:7|TG 16:1_18:3_18:3	TG	C55H92O6	866.7216	866.7232	[M+NH4]+	Medium confidence - possible other fatty acyl composition	3176.32	0.00026792	0.000667739	0.000670635
35.59	TG 54:9|TG 18:3_18:3_18:3	TG	C57H92O6	890.7219	890.7232	[M+NH4]+	Good confidence - confirmed fatty acyls composition	697.41	6.11739E-06	6.63188E-05	6.04819E-05
35.72	TG 52:9|TG 16:3_18:3_18:3	TG	C55H88O6	862.6915	862.6919	[M+NH4]+	Good confidence - confirmed fatty acyls composition	28244.35	0.000694017	0.00158677	0.002086842
35.81	TG 50:8|TG 18:2_16:3_16:3	TG	C53H86O6	836.6742	836.6763	[M+NH4]+	Good confidence - confirmed fatty acyls composition	680.01	9.27086E-05	5.64973E-05	7.53981E-05
35.86	TG 54:9|TG 18:3_18:3_18:3	TG	C57H92O6	890.7221	890.7232	[M+NH4]+	Good confidence - confirmed fatty acyls composition	1291.71	8.15292E-05	0.000271059	0.000163491
36.00	TG 54:7|TG 18:2_18:2_18:3	TG	C57H96O6	894.7528	894.7545	[M+NH4]+	Good confidence - confirmed fatty acyls composition	1374.2	0.000219017	0.000543827	3.6073E-05
36.26	TG 48:5|TG 16:1_16:2_16:2	TG	C51H88O6	814.6911	814.6919	[M+NH4]+	Good confidence - confirmed fatty acyls composition	174021.19	0.009405904	0.01316835	0.01418109
36.74	TG 50:6|TG 16:1_16:2_18:3	TG	C53H90O6	840.7059	840.7076	[M+NH4]+	Medium confidence - possible other fatty acyl composition	39031.02	0.009608357	0.0125937	0.01343749
37.14	TG 52:8|TG 16:2_18:3_18:3	TG	C55H90O6	864.7121	864.7076	[M+NH4]+	Good confidence - confirmed fatty acyls composition	85837.88	0.01977731	0.01821883	0.01977399
37.57	TG 54:9|TG 18:3_18:3_18:3	TG	C57H92O6	890.7243	890.7232	[M+NH4]+	Good confidence - confirmed fatty acyls composition	109937.97	0.01107522	0.01477106	0.01626823
37.67	TG 50:6|TG 16:0_17:3_17:3	TG	C53H90O6	840.7049	840.7076	[M+NH4]+	Medium confidence - possible other fatty acyl composition	2254.67	0.000247037	0.000521692	0.000726692
37.67	TG 52:8|TG 16:2_18:3_18:3	TG	C55H90O6	864.7058	864.7076	[M+NH4]+	Medium confidence - possible other fatty acyl composition	18694.74	0.002448479	0.002756622	0.003483848

37.76	TG 52:6|TG 18:1_16:2_18:3	TG	C55H94O6	868.7360	868.7389	[M+NH4]+	Good confidence - confirmed fatty acyls composition	1644.73	0.000346118	0.000555026	0.000778962
37.77	TG 54:6|TG 18:2_18:2_18:2	TG	C57H98O6	896.7702	896.7702	[M+NH4]+	Good confidence - confirmed fatty acyls composition	13622.46	0.000565965	0.002257724	0.000192178
37.85	TG 49:5|TG 16:1_16:2_17:2	TG	C52H90O6	828.7051	828.7076	[M+NH4]+	Medium confidence - possible other fatty acyl composition	4045.73	0.000412153	0.000310155	0.000366336
37.96	TG 48:5|TG 16:1_16:2_16:2	TG	C51H88O6	814.6904	814.6919	[M+NH4]+	Good confidence - confirmed fatty acyls composition	28731.09	0.003294058	0.001289869	0.001679305
38.15	TG 48:4|TG 16:0_16:2_16:2	TG	C51H90O6	816.7062	816.7076	[M+NH4]+	Good confidence - confirmed fatty acyls composition	36959.91	0.002758469	0.00172806	0.002104512
38.27	TG 51:6|TG 15:0_18:3_18:3	TG	C54H92O6	854.7210	854.7232	[M+NH4]+	Medium confidence - possible other fatty acyl composition	385.93	8.57809E-05	0.000110095	0.000118843
38.27	TG 53:7|TG 17:1_18:3_18:3	TG	C56H94O6	880.7374	880.7389	[M+NH4]+	Good confidence - confirmed fatty acyls composition	2223.94	7.48033E-05	0.000170086	0.000159132
38.49	TG 48:4|TG 16:1_16:1_16:2	TG	C51H90O6	816.7086	816.7076	[M+NH4]+	Good confidence - confirmed fatty acyls composition	96217.75	0.007528451	0.008841107	0.009825475
38.63	TG 50:5|TG 18:1_16:2_16:2	TG	C53H92O6	842.7225	842.7232	[M+NH4]+	Medium confidence - possible other fatty acyl composition	40459.24	0.01107132	0.01289977	0.01364109
38.69	TG 48:2|TG 16:0_16:1_16:1	TG	C51H94O6	820.7362	820.7389	[M+NH4]+	Good confidence - confirmed fatty acyls composition	27717.49	3.43459E-05	0.001433221	0.001809906
38.69	TG 50:4|TG 16:0_16:2_18:2	TG	C53H94O6	844.7354	844.7389	[M+NH4]+	Medium confidence - possible other fatty acyl composition	17508.65	0.001259756	0.006277061	0.006629226
39.11	TG 52:5|TG 16:0_18:2_18:3	TG	C55H96O6	870.7493	870.7545	[M+NH4]+	Good confidence - confirmed fatty acyls composition	65735.88	0.001694939	0.004347121	0.004478745
39.22	TG 49:4|TG 17:0_16:2_16:2	TG	C52H92O6	830.7216	830.7232	[M+NH4]+	Good confidence - confirmed fatty acyls composition	50782.01	0.007607355	0.003061581	0.002998797
39.36	TG 52:6|TG 16:0_18:3_18:3	TG	C55H94O6	868.7387	868.7389	[M+NH4]+	Good confidence - confirmed fatty acyls composition	29473	0.002901633	0.004209118	0.00510844
39.42	TG 54:8|TG 18:2_18:3_18:3	TG	C57H94O6	892.7400	892.7389	[M+NH4]+	Good confidence - confirmed fatty acyls composition	260402.92	0.01944913	0.01768904	0.01931417
39.67	TG 51:5|TG 17:0_16:2_18:3	TG	C54H94O6	856.7385	856.7389	[M+NH4]+	Medium confidence - possible other fatty acyl composition	111375.73	0.003753968	0.002833572	0.002911304
39.68	TG 51:6|TG 17:1_18:2_16:3	TG	C54H92O6	854.7206	854.7232	[M+NH4]+	Medium confidence - possible other fatty acyl composition	428.55	0.000343639	0.000141915	0.000128235
40.07	TG 51:5|TG 17:0_16:2_18:3	TG	C54H94O6	856.7371	856.7389	[M+NH4]+	Good confidence - confirmed fatty acyls composition	3544.79	0.001131888	0.000485486	0.000500512
40.08	TG 53:6|TG 17:1_18:2_18:3	TG	C56H96O6	882.7541	882.7545	[M+NH4]+	Good confidence - confirmed fatty acyls composition	29255.98	0.00089254	0.001111486	0.001114344
40.21	TG 50:5|TG 16:1_16:2_18:2	TG	C53H92O6	842.7233	842.7232	[M+NH4]+	Good confidence - confirmed fatty acyls composition	8123.33	0.004419879	0.001330558	0.001500523
40.35	TG 50:4|TG 16:0_16:1_18:3	TG	C53H94O6	844.7374	844.7389	[M+NH4]+	Medium confidence - possible other fatty acyl composition	10141.71	0.002955758	0.001796182	0.002002255

40.46	TG 53:6|TG 17:0_18:3_18:3	TG	C56H96O6	882.7538	882.7545	[M+NH4]+	Good confidence - confirmed fatty acyls composition	2482.94	0.000153782	6.93626E-05	0.000076505
40.62	TG 48:2|TG 16:0_16:0_16:2	TG	C51H94O6	820.7387	820.7389	[M+NH4]+	Good confidence - confirmed fatty acyls composition	36175.87	0.001244478	0.002784051	0.003073281
40.69	TG 50:4|TG 14:0_18:2_18:2	TG	C53H94O6	844.7394	844.7389	[M+NH4]+	Medium confidence - possible other fatty acyl composition	24644.28	0.009996246	0.01014226	0.0108851
40.77	TG 52:6|TG 16:2_18:2_18:2	TG	C55H94O6	868.7442	868.7389	[M+NH4]+	Good confidence - confirmed fatty acyls composition	88794.91	0.02269904	0.01629796	0.01716686
40.80	TG 52:5|TG 18:1_16:2_18:2	TG	C55H96O6	870.7520	870.7545	[M+NH4]+	Medium confidence - possible other fatty acyl composition	157863.83	0.011762	0.01007972	0.01047958
41.02	TG 50:3|TG 16:0_16:0_18:3	TG	C53H96O6	846.7542	846.7545	[M+NH4]+	Medium confidence - possible other fatty acyl composition	23869.82	0.000579863	0.001220638	0.001432237
41.07	TG 52:5|TG 16:0_18:2_18:3	TG	C55H96O6	870.7547	870.7545	[M+NH4]+	Good confidence - confirmed fatty acyls composition	115107.31	0.007143639	0.007705398	0.008776086
41.15	TG 54:7|TG 18:2_18:2_18:3	TG	C57H96O6	894.7584	894.7545	[M+NH4]+	Good confidence - confirmed fatty acyls composition	187419.47	0.02162184	0.01686702	0.01822185
41.31	TG 56:8|TG 20:2_18:3_18:3	TG	C59H98O6	920.7703	920.7702	[M+NH4]+	Medium confidence - possible other fatty acyl composition	22205.04	0.000821834	0.001305043	0.001347109
41.32	TG 49:2|TG 16:0_17:0_16:2	TG	C52H96O6	834.7542	834.7545	[M+NH4]+	Good confidence - confirmed fatty acyls composition	16441.6	0.000882771	0.000755738	0.000776342
41.36	TG 51:4|TG 17:0_16:2_18:2	TG	C54H96O6	858.7543	858.7545	[M+NH4]+	Good confidence - confirmed fatty acyls composition	99197.27	0.005846262	0.002675636	0.00248512
41.41	TG 54:4|TG 18:1_18:1_18:2	TG	C57H102O6	900.7979	900.8015	[M+NH4]+	Good confidence - confirmed fatty acyls composition	684.82	0.000289608	0.000815229	0.000138848
41.52	TG 54:6|TG 18:0_18:3_18:3	TG	C57H98O6	896.7690	896.7702	[M+NH4]+	Good confidence - confirmed fatty acyls composition	8195.19	0.000605563	0.000788034	0.000912645
41.56	TG 56:8|TG 20:2_18:3_18:3	TG	C59H98O6	920.7688	920.7702	[M+NH4]+	Good confidence - confirmed fatty acyls composition	4954.62	0.000516948	0.000433714	0.000471681
41.76	TG 53:5|TG 17:0_18:2_18:3	TG	C56H98O6	884.7703	884.7702	[M+NH4]+	Good confidence - confirmed fatty acyls composition	43645.66	0.002480663	0.00164658	0.001601921
41.79	TG 53:6|TG 17:2_17:2_19:2	TG	C56H96O6	882.7540	882.7545	[M+NH4]+	Good confidence - confirmed fatty acyls composition	3149.1	0.000215554	9.46052E-05	8.27289E-05
41.95	TG 50:2|TG 15:0_17:0_18:2	TG	C53H98O6	848.7694	848.7702	[M+NH4]+	Medium confidence - possible other fatty acyl composition	495.75	0.000117289	4.72814E-05	4.08844E-05
42.13	TG 53:5|TG 17:0_18:2_18:3	TG	C56H98O6	884.7692	884.7702	[M+NH4]+	Good confidence - confirmed fatty acyls composition	2101.52	0.000834716	0.000208131	0.000211324
42.30	TG 48:0|TG 16:0_16:0_16:0	TG	C51H98O6	824.7670	824.7702	[M+NH4]+	Good confidence - confirmed fatty acyls composition	732.76	2.39488E-05	4.15204E-05	5.27981E-05
42.30	TG 52:5|TG 18:1_16:2_18:2	TG	C55H96O6	870.7545	870.7545	[M+NH4]+	Good confidence - confirmed fatty acyls composition	12045.67	0.002354877	0.000547634	0.000596517
42.35	TG 50:2|TG 16:0_18:0_16:2	TG	C53H98O6	848.7681	848.7702	[M+NH4]+	Medium confidence - possible other fatty acyl composition	3605.57	0.000298166	0.000310268	0.000347186

42.39	TG 52:4|TG 18:0_16:2_18:2	TG	C55H98O6	872.7668	872.7702	[M+NH4]+	Good confidence - confirmed fatty acyls composition	12643.33	0.001644715	0.000862603	0.000874672
42.64	TG 48:0|TG 16:0_16:0_16:0	TG	C51H98O6	824.7704	824.7702	[M+NH4]+	Good confidence - confirmed fatty acyls composition	5499.98	0.000174541	0.000570954	0.000603795
42.65	TG 50:2|TG 14:0_18:0_18:2	TG	C53H98O6	848.7713	848.7702	[M+NH4]+	Medium confidence - possible other fatty acyl composition	68407.95	0.001365441	0.002555703	0.002681506
42.71	TG 52:4|TG 18:0_16:1_18:3	TG	C55H98O6	872.7706	872.7702	[M+NH4]+	Medium confidence - possible other fatty acyl composition	78616.39	0.007551924	0.007203699	0.007678206
42.76	TG 50:1|TG 16:0_16:0_18:1	TG	C53H100O6	850.7828	850.7858	[M+NH4]+	Good confidence - confirmed fatty acyls composition	1362.09	0.001009235	0.002373653	0.002734241
42.79	TG 54:6|TG 18:2_18:2_18:2	TG	C57H98O6	896.7717	896.7702	[M+NH4]+	Good confidence - confirmed fatty acyls composition	191871.19	0.01755026	0.01300368	0.01395672
42.87	TG 54:3|TG 18:1_18:1_18:1	TG	C57H104O6	902.8153	902.8171	[M+NH4]+	Good confidence - confirmed fatty acyls composition	8562.54	0.001226572	0.001714595	0.00035103
42.92	TG 56:7|TG 18:2_18:2_20:3	TG	C59H100O6	922.7850	922.7858	[M+NH4]+	Good confidence - confirmed fatty acyls composition	7520.41	0.001378386	0.001109723	0.001082178
43.11	TG 54:5|TG 20:0_16:1_18:4	TG	C57H100O6	898.7842	898.7858	[M+NH4]+	Good confidence - confirmed fatty acyls composition	54254.44	0.003123999	0.002232575	0.002296237
43.15	TG 56:7|TG 18:2_20:2_18:3	TG	C59H100O6	922.7836	922.7858	[M+NH4]+	Good confidence - confirmed fatty acyls composition	27940.19	0.002417679	0.00101166	0.001045882
43.28	TG 51:2|TG 18:0_16:1_17:1	TG	C54H100O6	862.7840	862.7858	[M+NH4]+	Medium confidence - possible other fatty acyl composition	6545.22	0.000585485	0.000461291	0.000407779
43.32	TG 53:4|TG 17:0_18:2_18:2	TG	C56H100O6	886.7844	886.7858	[M+NH4]+	Good confidence - confirmed fatty acyls composition	22205.45	0.001975126	0.000999602	0.000870469
43.64	TG 51:2|TG 16:0_17:0_18:2	TG	C54H100O6	862.7840	862.7858	[M+NH4]+	Medium confidence - possible other fatty acyl composition	972.55	0.000101728	6.77189E-05	5.93608E-05
43.69	TG 53:4|TG 17:0_18:2_18:2	TG	C56H100O6	886.7842	886.7858	[M+NH4]+	Good confidence - confirmed fatty acyls composition	2066.28	0.000656592	0.000165542	0.000153111
44.07	TG 55:5|TG 21:0_16:2_18:3	TG	C58H102O6	912.7985	912.8015	[M+NH4]+	Medium confidence - possible other fatty acyl composition	297.22	8.84738E-05	3.79537E-05	3.43455E-05
44.23	TG 54:5|TG 18:1_18:1_18:3	TG	C57H100O6	898.7831	898.7858	[M+NH4]+	Good confidence - confirmed fatty acyls composition	537.01	0.000529464	0.000101407	0.000101308
44.56	TG 52:2|TG 18:0_16:1_18:1	TG	C55H102O6	876.8007	876.8015	[M+NH4]+	Good confidence - confirmed fatty acyls composition	13903.35	0.000736596	0.000822859	0.00069187
44.63	TG 54:4|TG 22:0_16:2_16:2	TG	C57H102O6	900.8001	900.8015	[M+NH4]+	Good confidence - confirmed fatty acyls composition	42635.74	0.004293758	0.002548193	0.002359479
44.65	TG 56:6|TG 18:2_18:2_20:2	TG	C59H102O6	924.7996	924.8015	[M+NH4]+	Good confidence - confirmed fatty acyls composition	5970.75	0.00164824	0.000671664	0.000642924
44.97	TG 56:5|TG 22:0_16:2_18:3	TG	C59H104O6	926.8158	926.8171	[M+NH4]+	Good confidence - confirmed fatty acyls composition	9442.34	0.001354436	0.001286885	0.001294793
45.03	TG 51:0|TG 16:0_17:0_18:0	TG	C54H104O6	866.8146	866.8171	[M+NH4]+	Good confidence - confirmed fatty acyls composition	107.91	2.87423E-05	1.48248E-05	1.00963E-05

45.09	TG 53:2|TG 18:0_17:1_18:1	TG	C56H104O6	890.8154	890.8171	[M+NH4]+	Medium confidence - possible other fatty acyl composition	426.43	0.000115164	5.02076E-05	3.76812E-05
45.30	TG 58:6|TG 22:0_18:3_18:3	TG	C61H106O6	952.8315	952.8328	[M+NH4]+	Good confidence - confirmed fatty acyls composition	5199.71	0.000126984	0.000319121	0.000365344
45.55	TG 55:4|TG 23:0_16:2_16:2	TG	C58H104O6	914.8162	914.8171	[M+NH4]+	Good confidence - confirmed fatty acyls composition	1117.4	0.000374416	0.000136189	0.00011707
45.83	TG 57:5|TG 23:0_16:2_18:3	TG	C60H106O6	940.8314	940.8328	[M+NH4]+	Good confidence - confirmed fatty acyls composition	1787.18	0.000155632	9.06463E-05	8.23016E-05
46.29	TG 54:2|TG 22:0_16:1_16:1	TG	C57H106O6	904.8317	904.8328	[M+NH4]+	Medium confidence - possible other fatty acyl composition	8689.38	0.000423651	0.000420181	0.000345625
46.37	TG 56:4|TG 24:0_16:2_16:2	TG	C59H106O6	928.8297	928.8328	[M+NH4]+	Good confidence - confirmed fatty acyls composition	96312.2	0.004069515	0.002248377	0.002119565
46.56	TG 58:3|TG 22:0_18:1_18:2	TG	C61H112O6	958.8785	958.8797	[M+NH4]+	Medium confidence - possible other fatty acyl composition	7622.93	8.15056E-05	0.000407576	0.000129824
46.66	TG 58:5|TG 22:0_18:2_18:3	TG	C61H108O6	954.8478	954.8484	[M+NH4]+	Good confidence - confirmed fatty acyls composition	62993.73	0.001464578	0.001576184	0.001692057
46.96	TG 60:6|TG 24:0_18:3_18:3	TG	C63H110O6	980.8621	980.8641	[M+NH4]+	Good confidence - confirmed fatty acyls composition	6760.56	9.86221E-05	0.000291121	0.000330203
47.20	TG 57:4|TG 25:0_16:2_16:2	TG	C60H108O6	942.8467	942.8484	[M+NH4]+	Good confidence - confirmed fatty acyls composition	2762.84	0.000565856	0.000247864	0.0002026
47.46	TG 59:5|TG 23:0_18:2_18:3	TG	C62H110O6	968.8633	968.8641	[M+NH4]+	Good confidence - confirmed fatty acyls composition	3302.42	0.000183515	0.000161783	0.000136284
47.88	TG 56:2|TG 16:0_16:1_24:1	TG	C59H110O6	932.8615	932.8641	[M+NH4]+	Good confidence - confirmed fatty acyls composition	8864.98	0.000342993	0.000432246	0.000397827
47.95	TG 58:4|TG 24:0_16:2_18:2	TG	C61H110O6	956.8626	956.8641	[M+NH4]+	Good confidence - confirmed fatty acyls composition	84516.73	0.002694511	0.001833984	0.001754683
48.12	TG 60:3|TG 24:0_18:1_18:2	TG	C63H116O6	986.9079	986.9110	[M+NH4]+	Medium confidence - possible other fatty acyl composition	3653.12	5.24607E-05	0.000165389	7.72759E-05
48.22	TG 60:5|TG 24:0_18:2_18:3	TG	C63H112O6	982.8785	982.8797	[M+NH4]+	Good confidence - confirmed fatty acyls composition	31131.59	0.0006883	0.001030059	0.001060179
48.52	TG 62:6|TG 26:0_18:3_18:3	TG	C65H114O6	1008.8937	1008.8954	[M+NH4]+	Good confidence - confirmed fatty acyls composition	2391.92	2.13615E-05	9.5621E-05	9.71413E-05
48.70	TG 59:4|TG 27:0_16:2_16:2	TG	C62H112O6	970.8760	970.8797	[M+NH4]+	Good confidence - confirmed fatty acyls composition	3935.12	0.000320361	0.000196126	0.000154153
48.97	TG 61:5|TG 25:0_18:2_18:3	TG	C64H114O6	996.8947	996.8954	[M+NH4]+	Good confidence - confirmed fatty acyls composition	2160.31	8.2692E-05	0.000103782	8.84775E-05
49.01	TG 62:3|TG 18:1_22:1_22:1	TG	C65H120O6	1014.9421	1014.9423	[M+NH4]+	Good confidence - confirmed fatty acyls composition	5365.95	1.06107E-07	4.34316E-05	0.000290795
49.24	TG 58:2|TG 18:0_24:0_16:2	TG	C61H114O6	960.8944	960.8954	[M+NH4]+	Medium confidence - possible other fatty acyl composition	6785.58	0.000234616	0.00035094	0.00035095
49.31	TG 60:4|TG 26:0_16:2_18:2	TG	C63H114O6	984.8952	984.8954	[M+NH4]+	Good confidence - confirmed fatty acyls composition	43804.43	0.001247814	0.001202834	0.001168717

49.42	TG 62:5|TG 26:0_18:2_18:3	TG	C65H116O6	1010.9122	1010.9110	[M+NH4]+	Good confidence - confirmed fatty acyls composition	20831.2	0.000237769	0.000559436	0.000657903
49.73	TG 60:2|TG 18:0_18:1_24:1	TG	C63H118O6	988.9241	988.9267	[M+NH4]+	Medium confidence - possible other fatty acyl composition	4475.19	7.69088E-05	0.000126242	0.000140377
49.75	TG 62:4|TG 26:0_18:2_18:2	TG	C65H118O6	1012.9241	1012.9267	[M+NH4]+	Good confidence - confirmed fatty acyls composition	12060.05	0.00024821	0.000357481	0.00042551
49.81	TG 64:5|TG 28:0_18:2_18:3	TG	C67H120O6	1038.9406	1038.9423	[M+NH4]+	Good confidence - confirmed fatty acyls composition	2806.58	2.92872E-05	7.80431E-05	9.878E-05
49.98	TG 62:2|TG 16:0_28:0_18:2	TG	C65H122O6	1016.9562	1016.9580	[M+NH4]+	Good confidence - confirmed fatty acyls composition	835.73	1.48762E-05	2.35945E-05	2.46655E-05
50.00	TG 64:4|TG 28:0_18:2_18:2	TG	C67H122O6	1040.9552	1040.9580	[M+NH4]+	Good confidence - confirmed fatty acyls composition	1692.1	2.45582E-05	4.50039E-05	5.60485E-05
44.16	TG 64:7;2O|TG 16:2_16:2_18:2;O(FA 14:0)	TG-EST	C67H116O8	1066.8991	1066.9008	[M+NH4]+	Medium confidence - possible other fatty acyl composition	4512.46	8.78965E-06	8.59492E-05	0.000125715
44.17	TG 66:9;2O|TG 16:2_18:3_16:1;O(FA 16:2)	TG-EST	C69H116O8	1090.8993	1090.9008	[M+NH4]+	Medium confidence - possible other fatty acyl composition	13582.45	8.40222E-05	0.00039175	0.000596635
44.33	TG 68:10;O2|TG 16:2_18:3_16:2;O(FA 18:2)	TG-EST	C71H118O8	1116.9159	1116.9165	[M+NH4]+	Good confidence - confirmed fatty acyls composition	6896.58	3.9685E-05	0.000258663	0.000392702
45.06	TG 72:12;2O|TG 18:3_18:3_18:3;O(FA 18:2)	TG-EST	C75H122O8	1168.9476	1168.9478	[M+NH4]+	Medium confidence - possible other fatty acyl composition	1497.38	2.12798E-06	3.69547E-05	5.51072E-05
45.69	TG 68:9;2O|TG 18:2_18:2_14:1;O(FA 18:3)	TG-EST	C71H120O8	1118.9329	1118.9321	[M+NH4]+	Medium confidence - possible other fatty acyl composition	9007.27	5.61031E-05	0.000248161	0.00034575
45.72	TG 66:7;2O|TG 16:0_16:2_16:2;O(FA 18:2)	TG-EST	C69H120O8	1094.9312	1094.9321	[M+NH4]+	Medium confidence - possible other fatty acyl composition	3890.95	1.00335E-05	9.1754E-05	0.000126144
45.90	TG 70:10;O2|TG 16:2_18:3_18:1;O(FA 18:3)	TG-EST	C73H122O8	1144.9468	1144.9478	[M+NH4]+	Good confidence - confirmed fatty acyls composition	2449.17	2.6449E-05	0.000205174	0.000273748
47.15	TG 70:9;O2|TG 16:2_18:2_18:1;O(FA 18:3)	TG-EST	C73H124O8	1146.9624	1146.9634	[M+NH4]+	Good confidence - confirmed fatty acyls composition	2932.15	2.48007E-05	0.000134031	0.000165144
47.40	TG 72:10;2O|TG 18:2_18:2_18:2;O(FA 18:3)	TG-EST	C75H126O8	1172.9784	1172.9791	[M+NH4]+	Medium confidence - possible other fatty acyl composition	2048.29	8.23447E-06	5.88619E-05	6.5122E-05
47.70	TG 72:10;2O|TG 18:2_18:3_18:2;O(FA 18:2)	TG-EST	C75H126O8	1172.9771	1172.9791	[M+NH4]+	Medium confidence - possible other fatty acyl composition	1190.76	3.63303E-06	2.44086E-05	2.79294E-05
48.55	TG 72:9;2O|TG 18:2_18:3_18:2;O(FA 18:1)	TG-EST	C75H128O8	1174.9935	1174.9948	[M+NH4]+	Medium confidence - possible other fatty acyl composition	1184.49	4.88667E-06	2.86287E-05	3.06716E-05

Table S2. Free fatty acids (FA), fatty acyl esters of hydroxy fatty acids (FAHFA), and oxidized free fatty acids (OxFA) identified in Hydnocarpus wightianus oil by UPLC-QTOF-MS in ESI negative mode. Column descriptions as in Table S1. Six species within the FAHFA class carry the prefix AAHFA in their shorthand name, as returned by the annotation software. AAHFA denotes an acyl alpha-hydroxy fatty acid, that is, a fatty acid esterified to the hydroxyl group at C-2 of a hydroxy fatty acid (Yasuda et al., iScience 2020, 23, 101841, https://doi.org/10.1016/j.isci.2020.101841). Eleven oxidized fatty acids carry the suffix (2OH), the hydroxyl position proposed by the annotation software. Neither position was confirmed experimentally, and the oxidized fatty acids are referred to in the main text as FA x:y;O. The six AAHFA species are counted within the FAHFA class throughout this work. Signal intensities are relative normalized values obtained by mTIC normalization in MS-DIAL, as for Table S1. Species marked in red with a dagger are the three most intense features of the negative ion mode, for which the mass error exceeds 10 ppm (+27.1, +16.8 and +10.7 ppm), which is the behaviour expected when the detector response approaches saturation. All other features fall within 6.4 ppm.

RT (min)	Name of Lipid	Lipid class	Formula	Measured m/z	Reference m/z	Adduct form	Lipid identification level	Average signal to noise ratio	Signal intensity CHO 1	Signal intensity CHO 2	Signal intensity CHO 3
1.35	FA 15:3	FA	C15H24O2	235.1695	235.1704	[M-H]-	Good confidence - confirmed fatty acyls composition	59.24	9.87E-05	0.000102597	0.000185878
1.82	FA 17:1	FA	C17H32O2	267.2325	267.2330	[M-H]-	Good confidence - confirmed fatty acyls composition	111.63	1.76E-05	8.76E-05	3.05E-06
2.88	FA 16:3	FA	C16H26O2	249.1863	249.1860	[M-H]-	Good confidence - confirmed fatty acyls composition	496.27	0.000860893	0.002889411	0.002629048
3.06	FA 18:4	FA	C18H28O2	275.2010	275.2017	[M-H]-	Good confidence - confirmed fatty acyls composition	122.32	6.82E-06	0.000125287	3.08E-05
3.35	FA 18:3	FA	C18H30O2	277.2178	277.2173	[M-H]-	Good confidence - confirmed fatty acyls composition	880.06	0.003330845	0.005371484	0.008057994
3.72	FA 16:2†	FA	C16H28O2	251.2044	251.2017	[M-H]-	Good confidence - confirmed fatty acyls composition	9171.23	0.05346981	0.06962893	0.07067513
4.02	FA 16:1	FA	C16H30O2	253.2175	253.2173	[M-H]-	Good confidence - confirmed fatty acyls composition	6139.85	0.000371629	0.01545359	0.01848048
4.22	FA 18:3†	FA	C18H30O2	277.2248	277.2173	[M-H]-	Good confidence - confirmed fatty acyls composition	27365.57	0.0765856	0.1168213	0.1936324

4.31	FA 15:0	FA	C15H30 O2	241.2173	241.2173	[M-H]-	Good confidence - confirmed fatty acyls composition	102.89	4.30E-05	0.000173275	0.000136145
4.48	FA 18:2	FA	C18H32 O2	279.2345	279.2330	[M-H]-	Good confidence - confirmed fatty acyls composition	5779.97	0.02094379	0.03566906	0.03308792
4.55	FA 20:4	FA	C20H32 O2	303.2322	303.2330	[M-H]-	Good confidence - confirmed fatty acyls composition	132.51	7.89E-05	0.000119872	0.000257253
4.95	FA 16:1	FA	C16H30 O2	253.2163	253.2173	[M-H]-	Good confidence - confirmed fatty acyls composition	63.19	7.39E-05	0.000156363	0.000121334
5.00	FA 17:1	FA	C17H32 O2	267.2331	267.2330	[M-H]-	Good confidence - confirmed fatty acyls composition	127.03	0.000182163	0.000321634	0.000313447
5.80	FA 16:0	FA	C16H32 O2	255.2336	255.2330	[M-H]-	Good confidence - confirmed fatty acyls composition	1590.52	0.005659691	0.01135534	0.0135884
5.81	FA 18:2†	FA	C18H32 O2	279.2377	279.2330	[M-H]-	Good confidence - confirmed fatty acyls composition	21325.67	0.05612426	0.09352104	0.09811206
6.14	FA 18:1	FA	C18H34 O2	281.2499	281.2486	[M-H]-	Good confidence - confirmed fatty acyls composition	10473.9	0.0256245	0.04251622	0.0340162
6.18	FA 20:3	FA	C20H34 O2	305.2483	305.2486	[M-H]-	Good confidence - confirmed fatty acyls composition	1863.8	0.001542084	0.003307838	0.003970572
6.58	FA 17:0	FA	C17H34 O2	269.2485	269.2486	[M-H]-	Good confidence - confirmed fatty acyls composition	275.43	0.000304653	0.00062016	0.000327474
7.04	FA 17:0	FA	C17H34 O2	269.2476	269.2486	[M-H]-	Good confidence - confirmed fatty acyls composition	39.11	0.000218237	0.000107764	0.00013676
8.60	FA 20:2	FA	C20H36 O2	307.2640	307.2643	[M-H]-	Good confidence - confirmed fatty acyls composition	445.96	0.000500421	0.000933635	0.000645867
8.61	FA 18:0	FA	C18H36 O2	283.2645	283.2643	[M-H]-	Good confidence - confirmed fatty acyls composition	434.93	0.001746638	0.003273879	0.001441266
8.87	FA 20:1	FA	C20H38 O2	309.2796	309.2799	[M-H]-	Good confidence - confirmed fatty acyls composition	119.47	0.000224894	0.000623202	0.000367232
11.83	FA 20:0	FA	C20H40 O2	311.2953	311.2956	[M-H]-	Good confidence - confirmed fatty acyls composition	82.67	0.000262169	0.000776471	0.00033419
11.90	FA 22:1	FA	C22H42 O2	337.3111	337.3112	[M-H]-	Good confidence - confirmed fatty acyls composition	227.1	1.25E-05	0.00013991	0.001295227
15.04	FA 22:0	FA	C22H44 O2	339.3273	339.3269	[M-H]-	Good confidence - confirmed fatty acyls composition	723.66	0.000803385	0.00176246	0.000827521
21.58	FA 26:0	FA	C26H52 O2	395.3886	395.3895	[M-H]-	Good confidence - confirmed fatty acyls composition	336.26	0.000550962	0.000576407	0.000680019
23.33	FA 27:0	FA	C27H54 O2	409.4050	409.4051	[M-H]-	Good confidence - confirmed fatty acyls composition	512.15	0.000202729	0.000715188	0.00096875
31.34	FA 34:1	FA	C34H66 O2	505.4985	505.4990	[M-H]-	Good confidence - confirmed fatty acyls composition	229.98	0.000436672	0.00028203	0.000483232
31.79	FA 32:0	FA	C32H64 O2	479.4839	479.4834	[M-H]-	Good confidence - confirmed fatty acyls composition	204.76	0.000482654	0.000129463	0.000180106

34.31	FA 36:1	FA	C36H70 O2	533.5296	533.5303	[M-H]-	Good confidence - confirmed fatty acyls composition	222.35	0.000601675	1.95E-05	3.49E-05
34.43	FA 34:0	FA	C34H68 O2	507.5151	507.5147	[M-H]-	Good confidence - confirmed fatty acyls composition	251.86	0.000733682	0.000157106	0.000207935
36.80	FA 38:1	FA	C38H74 O2	561.5606	561.5616	[M-H]-	Good confidence - confirmed fatty acyls composition	157.98	0.000404883	3.81E-06	1.31E-05
13.39	FAHFA 32:5;2O|FAHFA 16:2/16:2;O	FAHFA	C32H54 O4	501.3943	501.3949	[M-H]-	Good confidence - confirmed fatty acyls composition	1668.51	1.12E-05	0.001423228	1.59E-05
13.60	FAHFA 32:5;2O|FAHFA 16:2/16:2;O	FAHFA	C32H54 O4	501.3943	501.3949	[M-H]-	Good confidence - confirmed fatty acyls composition	1376.8	0.000204921	0.001164283	1.93E-05
14.51	FAHFA 34:6;2O|FAHFA 18:3/16:2;O	FAHFA	C34H56 O4	527.4105	527.4106	[M-H]-	Good confidence - confirmed fatty acyls composition	1654.08	0.000179582	0.00221887	6.12E-05
14.57	AAHFA 32:5;2O|AAHFA 16:2/16:2;O	FAHFA	C32H54 O4	501.3951	501.3949	[M-H]-	Good confidence - confirmed fatty acyls composition	2272.54	0.000617319	0.005126228	0.001550046
14.95	FAHFA 34:6;2O|FAHFA 16:2/18:3;O	FAHFA	C34H56 O4	527.4111	527.4106	[M-H]-	Good confidence - confirmed fatty acyls composition	2341.5	0.000244681	0.002990004	0.000187136
15.29	FAHFA 36:7;2O|FAHFA 18:3/18:3;O	FAHFA	C36H58 O4	553.4264	553.4262	[M-H]-	Good confidence - confirmed fatty acyls composition	3388.85	0	0.002202349	0
15.90	FAHFA 34:6;2O|FAHFA 16:2/18:3;O	FAHFA	C34H56 O4	527.4108	527.4106	[M-H]-	Good confidence - confirmed fatty acyls composition	249.63	2.56E-05	0.000147031	0.001579602
15.97	FAHFA 34:5;2O|FAHFA 16:2/18:2;O	FAHFA	C34H58 O4	529.4260	529.4262	[M-H]-	Good confidence - confirmed fatty acyls composition	1603.68	8.99E-05	0.001829953	0.000202714
16.14	FAHFA 34:5;2O|FAHFA 16:2/18:2;O	FAHFA	C34H58 O4	529.4258	529.4262	[M-H]-	Good confidence - confirmed fatty acyls composition	2031.92	5.24E-05	0.00275722	0.00017148
16.15	FAHFA 32:3;2O|FAHFA 16:0/16:2;O	FAHFA	C32H58 O4	505.4247	505.4262	[M-H]-	Good confidence - confirmed fatty acyls composition	298.28	8.49E-06	0.000491145	3.09E-05
16.41	FAHFA 32:3;2O|FAHFA 16:0/16:2;O	FAHFA	C32H58 O4	505.4246	505.4262	[M-H]-	Good confidence - confirmed fatty acyls composition	265.82	0	0.000328676	0
16.52	FAHFA 36:6;2O|FAHFA 18:3/18:2;O	FAHFA	C36H60 O4	555.4403	555.4419	[M-H]-	Good confidence - confirmed fatty acyls composition	783.27	3.10E-05	0.000742619	1.04E-05

16.85	AAHFA 36:5;2O|AAHFA 18:3/18:1;O	FAHFA	C36H62 O4	557.4566	557.4575	[M-H]-	Good confidence - confirmed fatty acyls composition	1573.24	2.72E-05	0.001057543	7.37E-05
16.89	FAHFA 36:6;2O|FAHFA 18:2/18:3;O	FAHFA	C36H60 O4	555.4418	555.4419	[M-H]-	Good confidence - confirmed fatty acyls composition	2833.02	0.000170516	0.002132928	0.000270175
17.13	FAHFA 36:6;2O|FAHFA 18:3/18:2;O	FAHFA	C36H60 O4	555.4424	555.4419	[M-H]-	Good confidence - confirmed fatty acyls composition	12558.46	5.61E-05	0.008656598	5.96E-05
17.35	AAHFA 32:3;2O|AAHFA 16:0/16:2;O	FAHFA	C32H58 O4	505.4268	505.4262	[M-H]-	Good confidence - confirmed fatty acyls composition	1895.56	8.99E-05	0.003951048	0.001570083
17.48	AAHFA 34:4;2O|AAHFA 16:2/18:1;O	FAHFA	C34H60 O4	531.4412	531.4419	[M-H]-	Good confidence - confirmed fatty acyls composition	6568.28	0	0.003842397	0
17.51	FAHFA 34:5;2O|FAHFA 16:2/18:2;O	FAHFA	C34H58 O4	529.4258	529.4262	[M-H]-	Good confidence - confirmed fatty acyls composition	1555.94	0.002759095	0.02972165	0.008554959
17.84	FAHFA 36:5;2O|FAHFA 18:1/18:3;O	FAHFA	C36H62 O4	557.4570	557.4575	[M-H]-	Good confidence - confirmed fatty acyls composition	1596.24	0	0.002047374	0.000691101
18.13	FAHFA 36:6;2O|FAHFA 18:3/18:2;O	FAHFA	C36H60 O4	555.4414	555.4419	[M-H]-	Good confidence - confirmed fatty acyls composition	580.94	0.000254908	0.000172062	0.002170041
18.19	FAHFA 36:6;2O|FAHFA 18:2/18:3;O	FAHFA	C36H60 O4	555.4419	555.4419	[M-H]-	Good confidence - confirmed fatty acyls composition	2647.11	0.000254908	0.003286746	0.00100879
18.65	FAHFA 36:5;2O|FAHFA 18:2/18:2;O	FAHFA	C36H62 O4	557.4565	557.4575	[M-H]-	Good confidence - confirmed fatty acyls composition	827.56	7.85E-05	0.000981632	0.000595791
18.67	FAHFA 34:3;2O|FAHFA 16:0/18:2;O	FAHFA	C34H62 O4	533.4576	533.4575	[M-H]-	Good confidence - confirmed fatty acyls composition	602.37	2.46E-05	0.000696963	0.000400224
18.88	FAHFA 34:2;2O|FAHFA 18:1/16:0;O	FAHFA	C34H64 O4	535.4698	535.4732	[M-H]-	Good confidence - confirmed fatty acyls composition	276.21	0	0.000188988	0
18.94	FAHFA 36:5;2O|FAHFA 18:2/18:2;O	FAHFA	C36H62 O4	557.4567	557.4575	[M-H]-	Good confidence - confirmed fatty acyls composition	852.97	0	0.000961774	0
19.14	FAHFA 36:4;2O|FAHFA 18:2/18:1;O	FAHFA	C36H64 O4	559.4720	559.4732	[M-H]-	Good confidence - confirmed fatty acyls composition	320.73	0	0.000364425	0.000326362

19.71	FAHFA 36:4;2O|FAHFA 18:1/18:2;O	FAHFA	C36H64 O4	559.4724	559.4732	[M-H]-	Good confidence - confirmed fatty acyls composition	6677.76	0	0.003839909	0
20.95	AAHFA 36:4;2O|AAHFA 18:2/18:1;O	FAHFA	C36H64 O4	559.4727	559.4732	[M-H]-	Good confidence - confirmed fatty acyls composition	1118.62	3.50E-05	0.00142637	0.000343799
20.98	AAHFA 34:2;2O|AAHFA 16:0/18:1;O	FAHFA	C34H64 O4	535.4728	535.4732	[M-H]-	Good confidence - confirmed fatty acyls composition	656.9	4.96E-05	0.000739985	0.000372302
21.62	FAHFA 36:3;2O|FAHFA 18:2/18:0;O	FAHFA	C36H66 O4	561.4883	561.4888	[M-H]-	Good confidence - confirmed fatty acyls composition	152.68	2.22E-06	0.000197641	0.000323912
21.69	FAHFA 34:1;2O|FAHFA 16:0/18:0;O	FAHFA	C34H66 O4	537.4888	537.4888	[M-H]-	Good confidence - confirmed fatty acyls composition	210.7	7.83E-06	0.000155287	3.65E-06
1.11	FA 16:0;O2	OxFA	C16H32 O4	287.2226	287.2228	[M-H]-	Good confidence - confirmed fatty acyls composition	1299.7	3.23E-05	0.000965617	0.000334246
1.17	FA 18:4;(2OH)	OxFA	C18H28 O3	291.1967	291.1966	[M-H]-	Good confidence - confirmed fatty acyls composition	7750.03	0.001849903	0.03098294	0.001454052
1.20	FA 18:1;O2	OxFA	C18H34 O4	313.2376	313.2384	[M-H]-	Good confidence - confirmed fatty acyls composition	294.87	0.001561496	0.001218371	0.000712214
1.21	FA 16:2;O	OxFA	C16H28 O3	267.1962	267.1966	[M-H]-	Good confidence - confirmed fatty acyls composition	438.02	0.002172396	0.00195249	0.001057759
1.36	FA 18:3;(2OH)	OxFA	C18H30 O3	293.2137	293.2122	[M-H]-	Good confidence - confirmed fatty acyls composition	11347.97	0.009636711	0.05336811	0.005508142
1.45	FA 18:0;O2	OxFA	C18H36 O4	315.2545	315.2541	[M-H]-	Good confidence - confirmed fatty acyls composition	536.76	0.000453734	0.000879114	0.000489072
1.46	FA 16:2;(2OH)	OxFA	C16H28 O3	267.1968	267.1966	[M-H]-	Good confidence - confirmed fatty acyls composition	524.2	0.002442246	0.001602215	0.001874421
1.62	FA 16:0;(2OH)	OxFA	C16H32 O3	271.2281	271.2279	[M-H]-	Good confidence - confirmed fatty acyls composition	560.04	0.000114682	0.001045175	0.000265569
1.62	FA 18:2;(2OH)	OxFA	C18H32 O3	295.2278	295.2279	[M-H]-	Good confidence - confirmed fatty acyls composition	2888.23	0.007907624	0.009495113	0.009617638
1.64	FA 18:3;(2OH)	OxFA	C18H30 O3	293.2116	293.2122	[M-H]-	Good confidence - confirmed fatty acyls composition	1357.92	0.01387112	0.004816862	0.004874975
1.96	FA 18:1;(2OH)	OxFA	C18H34 O3	297.2440	297.2435	[M-H]-	Good confidence - confirmed fatty acyls composition	223.13	0.000692422	0.001438889	0.000946384
2.22	FA 20:2;(2OH)	OxFA	C20H36 O3	323.2582	323.2592	[M-H]-	Good confidence - confirmed fatty acyls composition	77.05	1.48E-05	9.45E-05	1.29E-05
2.44	FA 18:0;(2OH)	OxFA	C18H36 O3	299.2586	299.2592	[M-H]-	Good confidence - confirmed fatty acyls composition	274.41	0.000743831	0.001105415	0.000365951

2.64	FA 18:2;(2OH)	OxFA	C18H32 O3	295.2278	295.2279	[M-H]-	Good confidence - confirmed fatty acyls composition	187.23	0.000647958	0.000708791	0.000548849
2.68	FA 18:1;(2OH)	OxFA	C18H34 O3	297.2426	297.2435	[M-H]-	Good confidence - confirmed fatty acyls composition	72.89	0.00069544	0.000494739	0.00125135

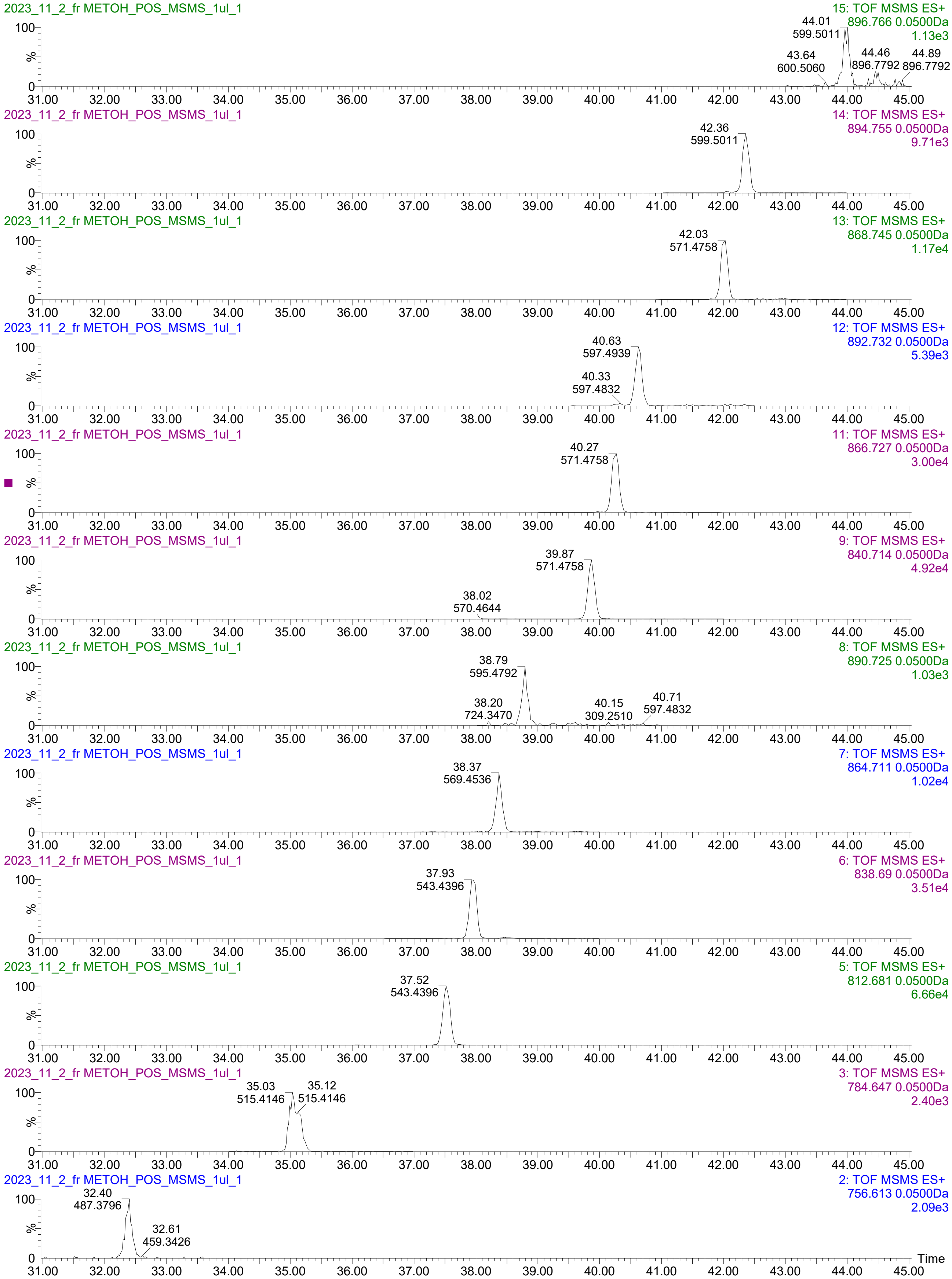

2023_11_2_fr METOH_POS_MSMS_1ul_1
15: TOF MSMS ES+
896.766 0.0500Da
1.13e3
44.01
599.5011
43.64
600.5060
44.46
896.7792
44.89
896.7792
2023_11_2_fr METOH_POS_MSMS_1ul_1
14: TOF MSMS ES+
894.755 0.0500Da
9.71e3
42.36
599.5011
2023_11_2_fr METOH_POS_MSMS_1ul_1
13: TOF MSMS ES+
868.745 0.0500Da
1.17e4
42.03
571.4758
2023_11_2_fr METOH_POS_MSMS_1ul_1
12: TOF MSMS ES+
892.732 0.0500Da
5.39e3
40.63
597.4939
40.33
597.4832
2023_11_2_fr METOH_POS_MSMS_1ul_1
11: TOF MSMS ES+
866.727 0.0500Da
3.00e4
40.27
571.4758
2023_11_2_fr METOH_POS_MSMS_1ul_1
9: TOF MSMS ES+
840.714 0.0500Da
4.92e4
39.87
571.4758
38.02
570.4644
2023_11_2_fr METOH_POS_MSMS_1ul_1
8: TOF MSMS ES+
890.725 0.0500Da
1.03e3
38.79
595.4792
38.20
724.3470
40.15
309.2510
40.71
597.4832
2023_11_2_fr METOH_POS_MSMS_1ul_1
7: TOF MSMS ES+
864.711 0.0500Da
1.02e4
38.37
569.4536
2023_11_2_fr METOH_POS_MSMS_1ul_1
6: TOF MSMS ES+
838.69 0.0500Da
3.51e4
37.93
543.4396
2023_11_2_fr METOH_POS_MSMS_1ul_1
5: TOF MSMS ES+
812.681 0.0500Da
6.66e4
37.52
543.4396
2023_11_2_fr METOH_POS_MSMS_1ul_1
3: TOF MSMS ES+
784.647 0.0500Da
2.40e3
35.03
515.4146
35.12
515.4146
2023_11_2_fr METOH_POS_MSMS_1ul_1
2: TOF MSMS ES+
756.613 0.0500Da
2.09e3
32.40
487.3796
32.61
459.3426
100
%
0
31.00 32.00 33.00 34.00 35.00 36.00 37.00 38.00 39.00 40.00 41.00 42.00 43.00 44.00 45.00
Time

Figure S3. Extracted mass chromatograms for the triacylglycerol precursor ions selected for the MS/MS experiment, acquired for the methanolic extract. Each trace corresponds to one MS/MS function and is labelled with the m/z of the selected [M+NH4]+ precursor ion. The precursors correspond to the following triacylglycerols, assigned by accurate mass against the annotations of Table S1: m/z 896.766, TG 54:6 (18:2_18:2_18:2); m/z 894.755, TG 54:7 (18:2_18:2_18:3); m/z 892.732, TG 54:8 (18:2_18:3_18:3); m/z 890.725, TG 54:9 (18:3_18:3_18:3); m/z 868.745, TG 52:6 (16:2_18:2_18:2); m/z 866.727, TG 52:7 (16:1_18:3_18:3); m/z 864.711, TG 52:8 (16:2_18:3_18:3); m/z 840.714, TG 50:6 (16:1_16:2_18:3); m/z 838.690, TG 50:7 (16:2_16:2_18:3); m/z 812.681, TG 48:6; m/z 784.647, TG 46:6; and m/z 756.613, TG 44:6. All assignments agree with the reference masses to within 8 mDa. The three lowest-mass precursors are given at the sum-composition level only, because the corresponding features were not annotated in the oil samples of Table S1, the MS/MS experiment having been performed on the methanolic extract.

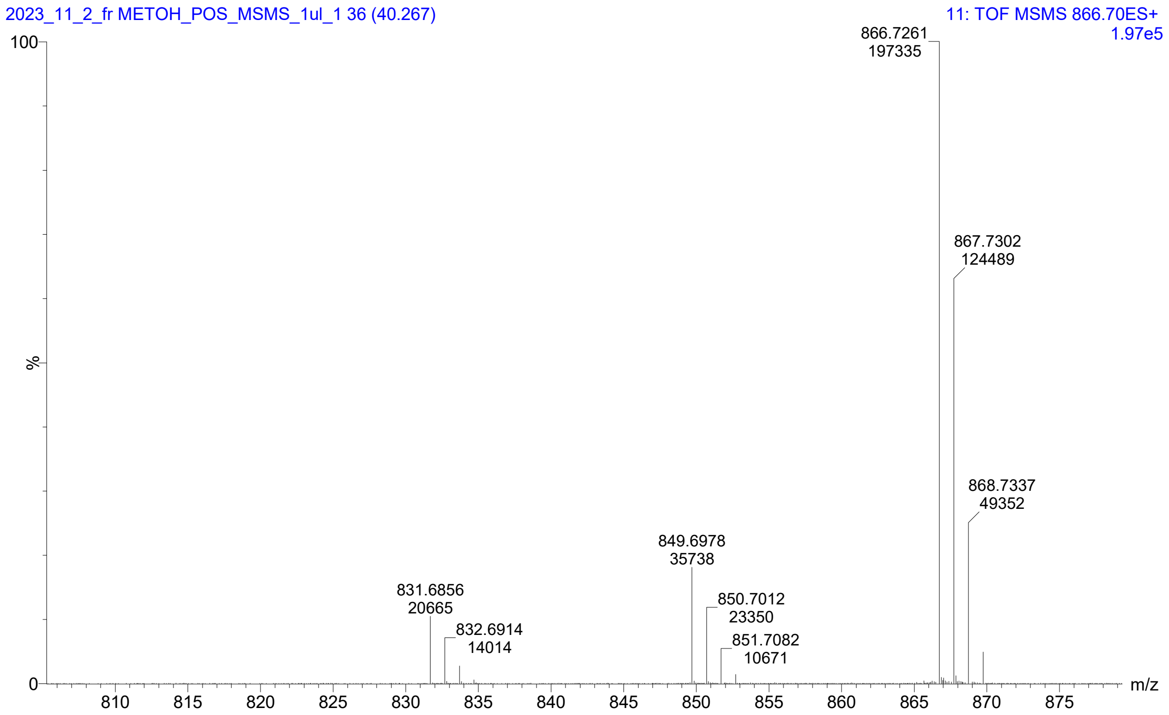

Figure S4. Fragment of the MS/MS mass spectrum for triacylglycerol consisting of chaulmoogric, hydnocarpic and gorlic acid residues. Visible signals coming from the precursor ions [M+NH4]+, [M+H]+ and [M-H2O+H]+. The precursor ions appear at m/z 866.72 ([M+NH4]+), 849.70 ([M+H]+), and 831.69 ([M-H2O+H]+), calculated for C55H92O6 (TG 52:7). The precursor at m/z 866.72 is annotated in Table S1 as TG 52:7 with the acyl composition 16:1_18:3_18:3, whereas the fragmentation shown in Figures S4 to S6 corresponds to the composition 16:2_18:2_18:3, that is chaulmoogric, hydnocarpic and gorlic acid residues. Both correspond to the same sum composition and the two isomers were not resolved under the conditions used.

Fragment ion nomenclature used in Figures S4 to S9. [M+H]+, [M+NH4]+, and [M+Na]+ denote the protonated, ammoniated, and sodiated precursor ions, and [M-H2O+H]+ the protonated precursor after loss of water. [M-RCO2]+ denotes the fragment formed by neutral loss of one fatty acid from the precursor, yielding a diacylglycerol-type ion from a triacylglycerol or a monoacylglycerol-type ion from a diacylglycerol, which identifies the remaining acyl chains. [RCO+74]+ denotes the protonated monoacylglycerol-type fragment of a single fatty acyl, that is, the acylium plus the C3H6O2 glycerol residue. [RCO]+ denotes the acylium ion of the fatty acid, and [RC]+ the hydrocarbon fragment formed by subsequent loss of water. *The signals at m/z 67 and 81 correspond to the cyclopentenyl (C5H7+) and methylcyclopentenyl (C6H9+) cations and are diagnostic of the cyclopentenyl ring of chaulmoogra fatty acids.*

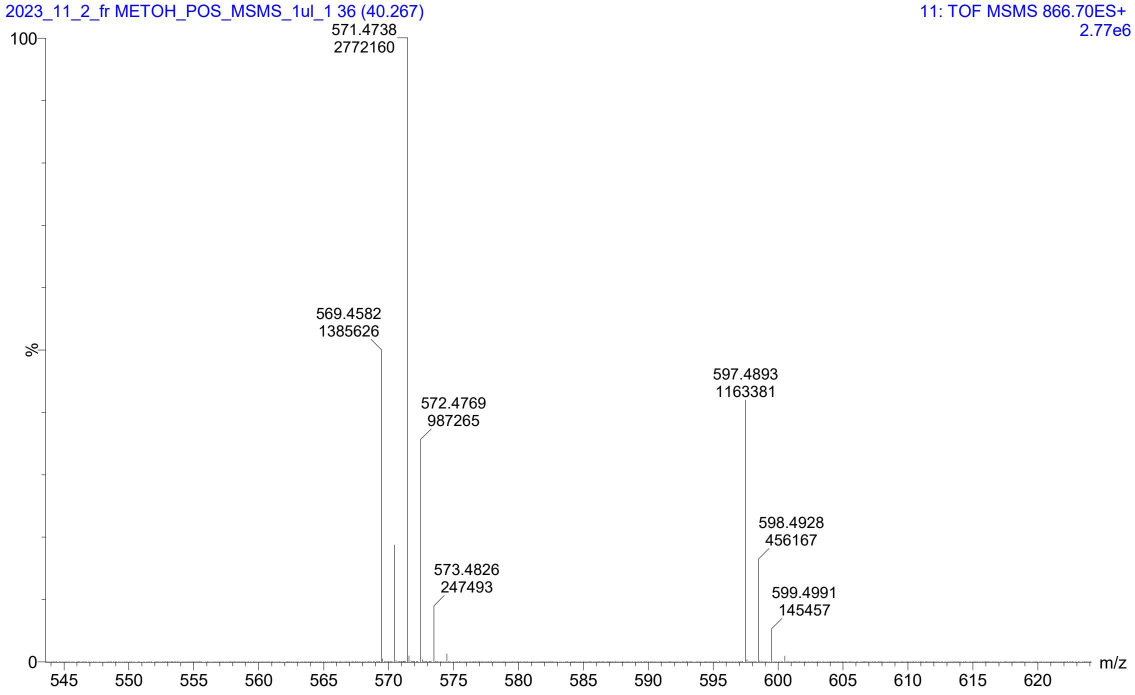

Figure S5. Fragment of the MS/MS mass spectrum for triacylglycerol consisting of chaulmoogric, hydnocarpic and gorlic acid residues. Signals from [M-RCO2]+ fragment ions are visible. Each [M-RCO2]+ ion corresponds to the neutral loss of one fatty acid from the protonated precursor, with the two remaining acyls retained. Loss of hydnocarpic acid (16:2) yields m/z 597.49, loss of chaulmoogric acid (18:2) yields m/z 569.46, and loss of gorlic acid (18:3) yields m/z 571.47.

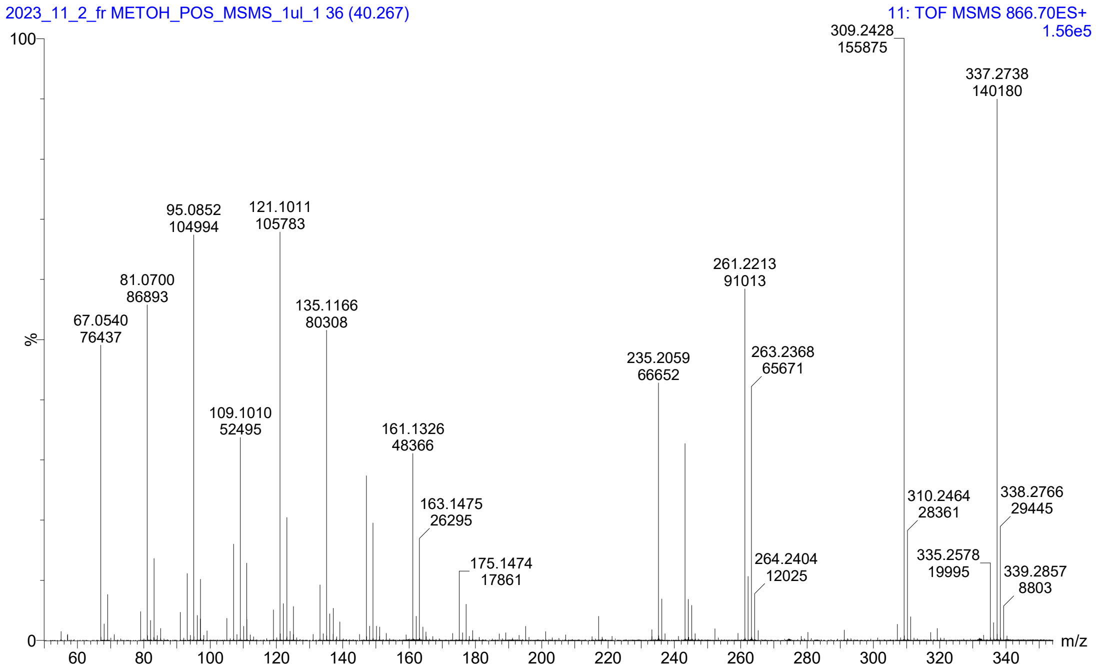

Figure S6. Fragment of the MS/MS mass spectrum for triacylglycerol consisting of chaulmoogric, hydnocarpic and gorlic acid residues. Visible signals coming from fragment ions [RCO+74]+, [RCO]+, [RC]+. Additionally, characteristic signals of 67 and 81 m/z are visible, indicating the presence of a cyclopentenyl ring in the structure of fatty acid residues. The [RCO]+ acylium ions appear at m/z 235.21, 263.24, and 261.22 for hydnocarpic (16:2), chaulmoogric (18:2), and gorlic (18:3) acids respectively. The corresponding [RCO+74]+ ions appear at m/z 309.24, 337.27, and 335.26, and the [RC]+ ions, formed by loss of water from the corresponding acylium ion, at m/z 217.20, 245.23, and 243.21. The signals at m/z 67 ($C_5H_7^+$) and 81 ($C_6H_9^+$) are diagnostic of the cyclopentenyl ring.

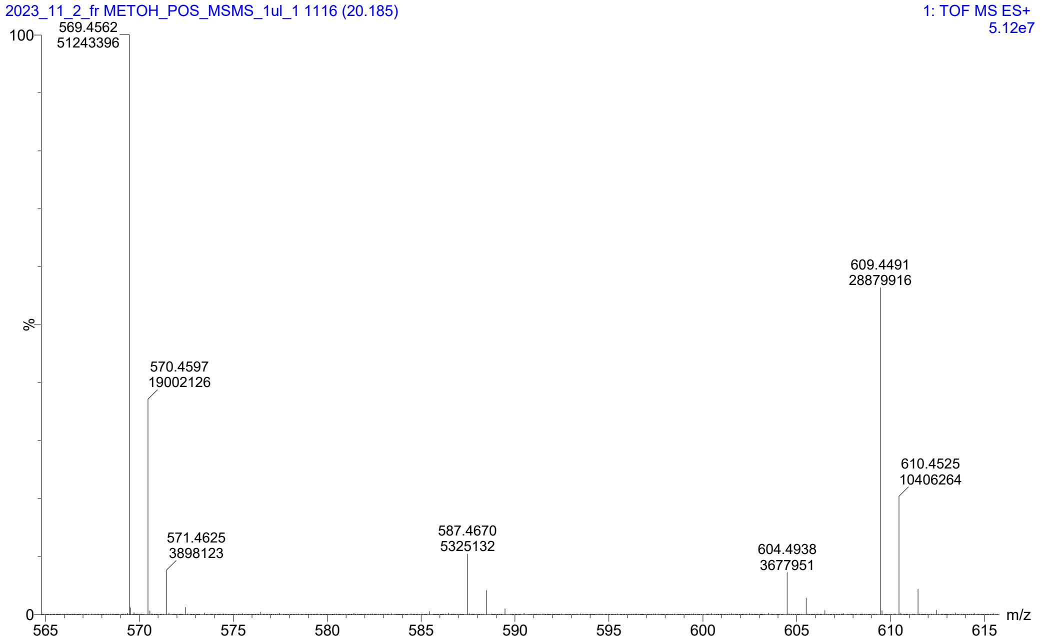

Figure S7. Fragment of the MS/MS mass spectrum for a diacylglycerol consisting of hydnocarpic and gorlic acid residues. Visible signals coming from precursor ions [M+Na]+, [M+NH4]+, [M+H]+ and [M-H2O+H]+. The precursor ions appear at m/z 609.45 ([M+Na]+), 604.49 ([M+NH4]+), 587.47 ([M+H]+), and 569.46 ([M-H2O+H]+), calculated for C37H62O5 (DG 34:5).

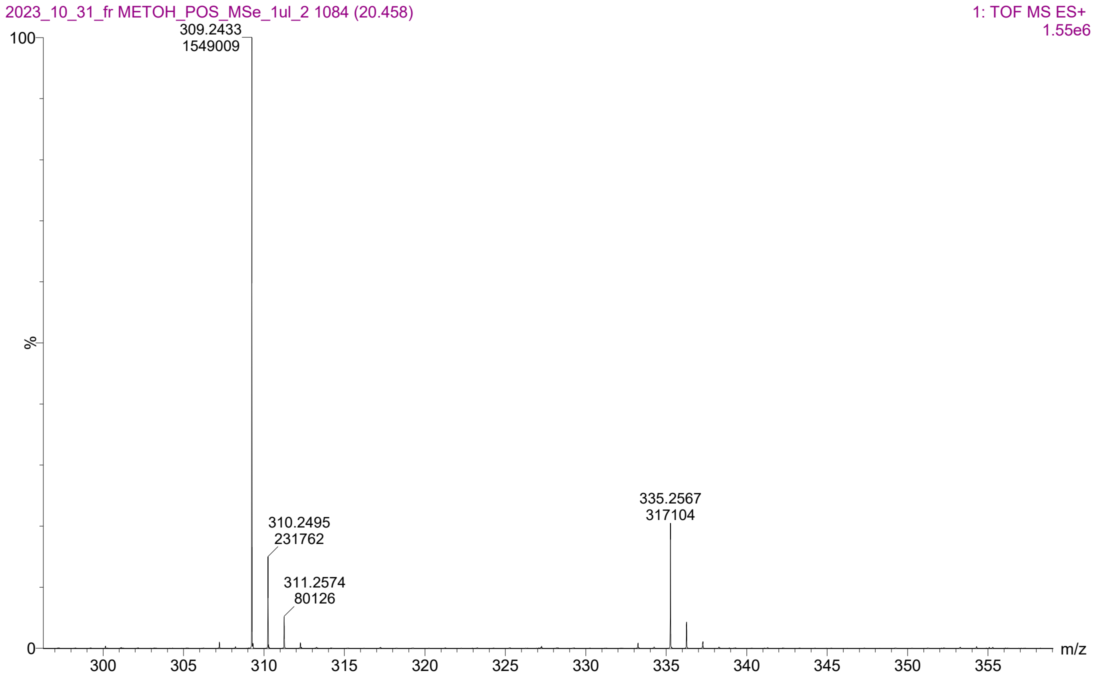

Figure S8. Fragment of the MS/MS mass spectrum for a diacylglycerol consisting of hydnocarpic and gorlic acid residues. Signals from fragment ions [RCO+74]+ and low-intensity signals from fragment ions [M-RCO2]+ are visible. For this diacylglycerol, neutral loss of one fatty acid from the protonated precursor yields the monoacylglycerol-type ion of the remaining acyl, so the [RCO+74]+ and [M-RCO2]+ labels describe the same fragments: m/z 309.24, retaining hydnocarpic acid (16:2) and equivalent to the loss of gorlic acid, and m/z 335.26, retaining gorlic acid (18:3) and equivalent to the loss of hydnocarpic acid.

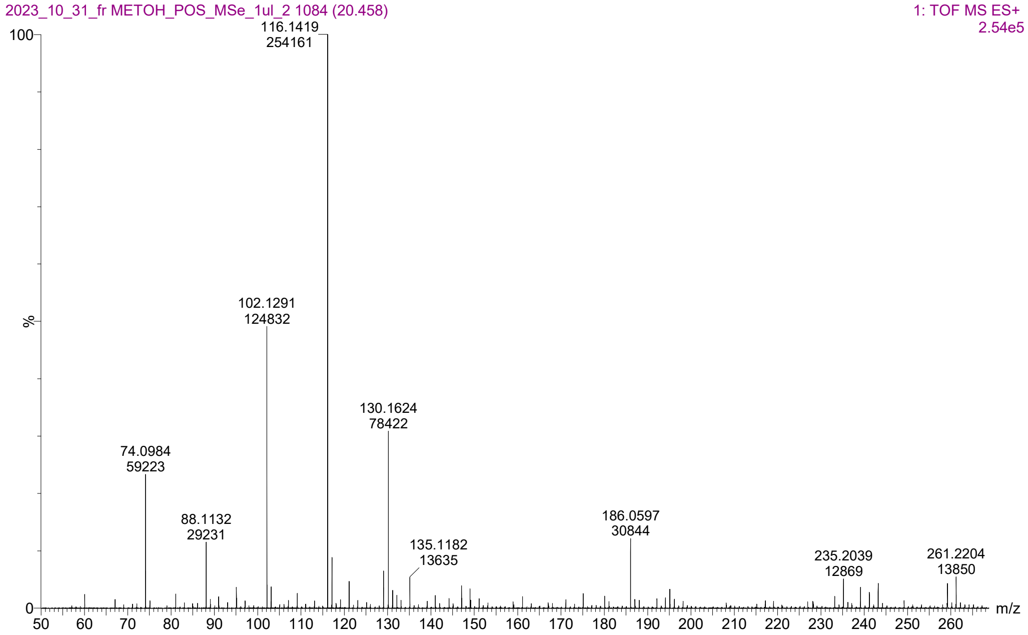

Figure S9. Fragment of the MS/MS mass spectrum for a diacylglycerol consisting of hydnocarpic and gorlic acid residues. Visible signals coming from fragment ions [RCO]+, [RC]+. The [RCO]+ acylium ions appear at m/z 235.21 (hydnocarpic, 16:2) and 261.22 (gorlic, 18:3), and the [RC]+ ions, formed by loss of water from the corresponding acylium ion, at m/z 217.20 and 243.21 respectively.

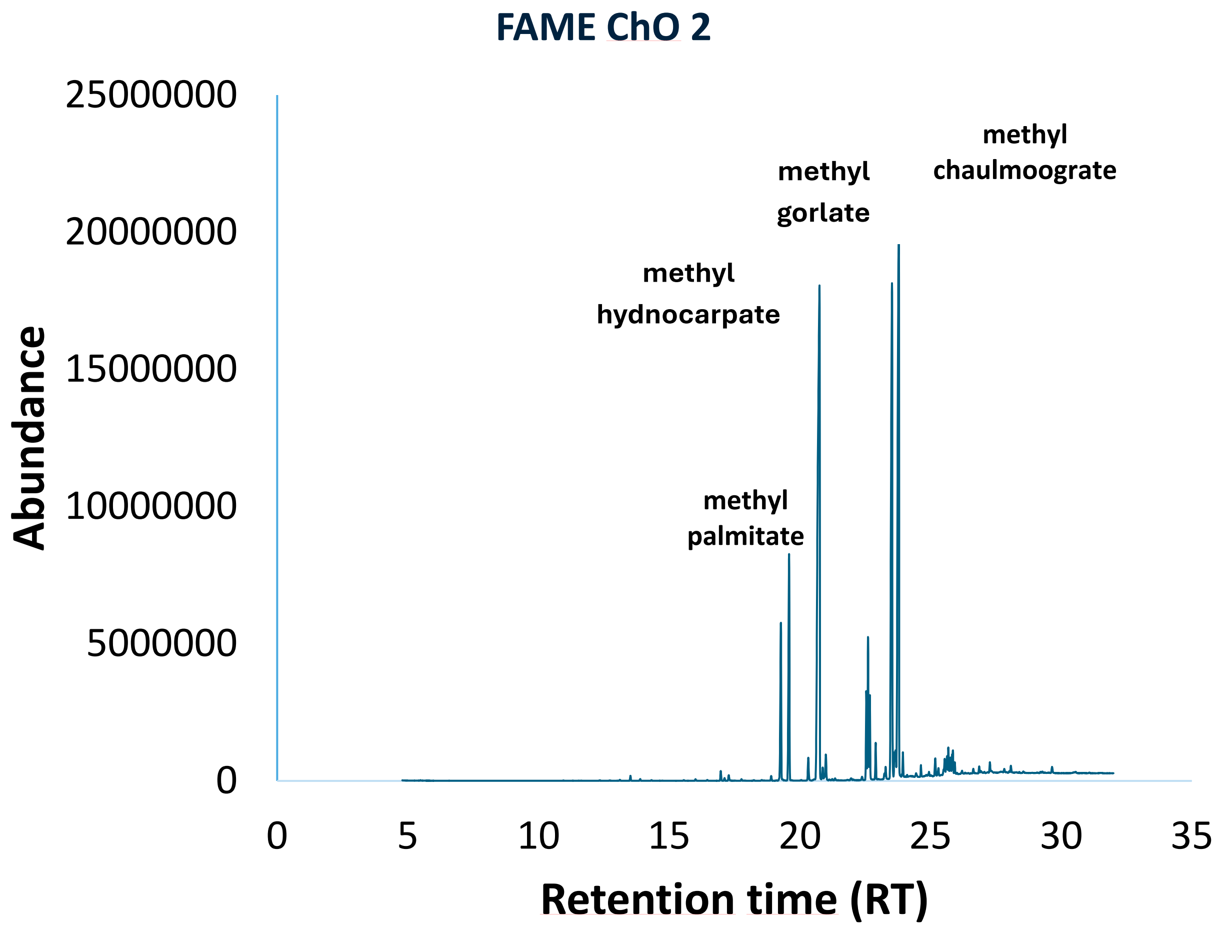

Figure S10. GC-MS chromatogram of the ChO 2 oil sample acquired on the ZB-5HT column. Identified fatty acid methyl esters are listed in Table 2 of the main text, and the numbered peak assignments are given in Table S3.

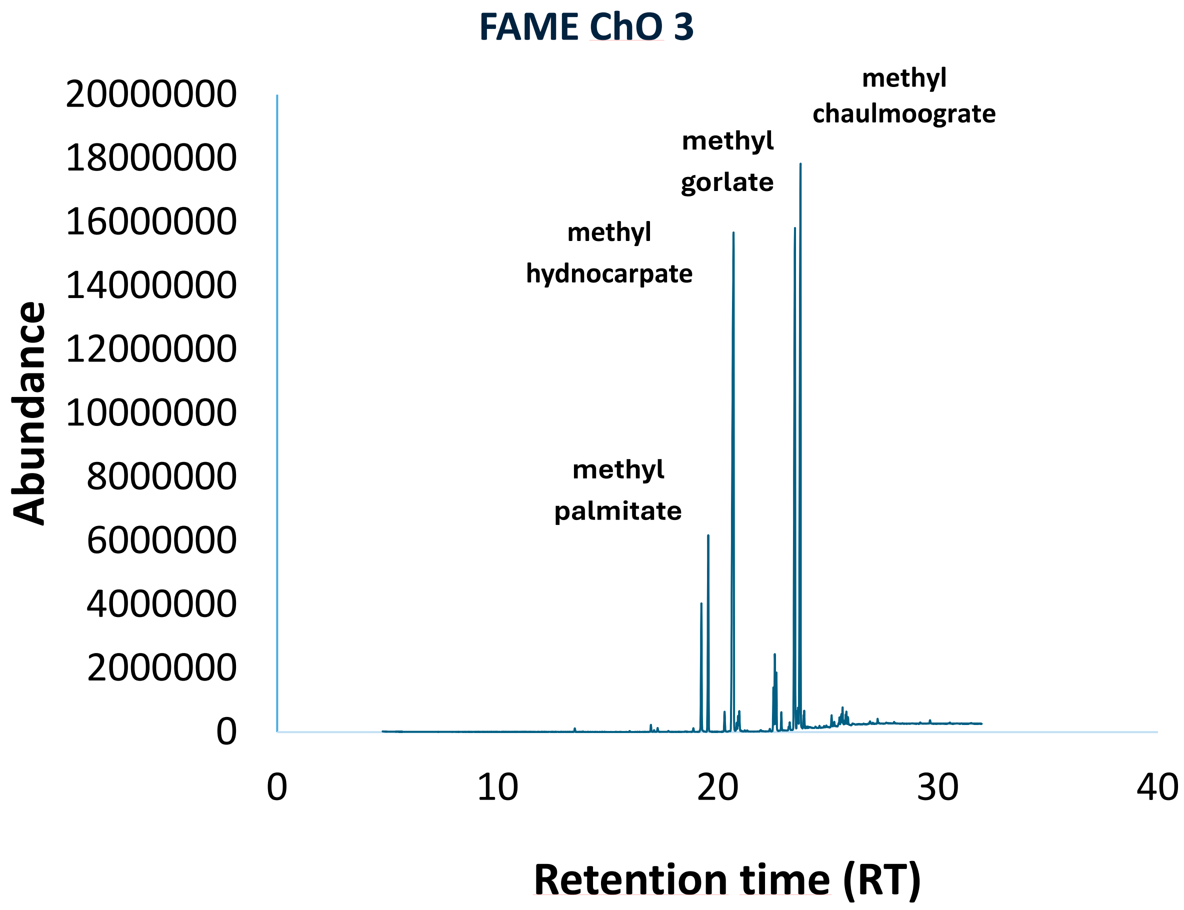

Figure S11. GC-MS chromatogram of the ChO 3 oil sample acquired on the ZB-5HT column. Identified fatty acid methyl esters are listed in Table 2 of the main text, and the numbered peak assignments are given in Table S3.

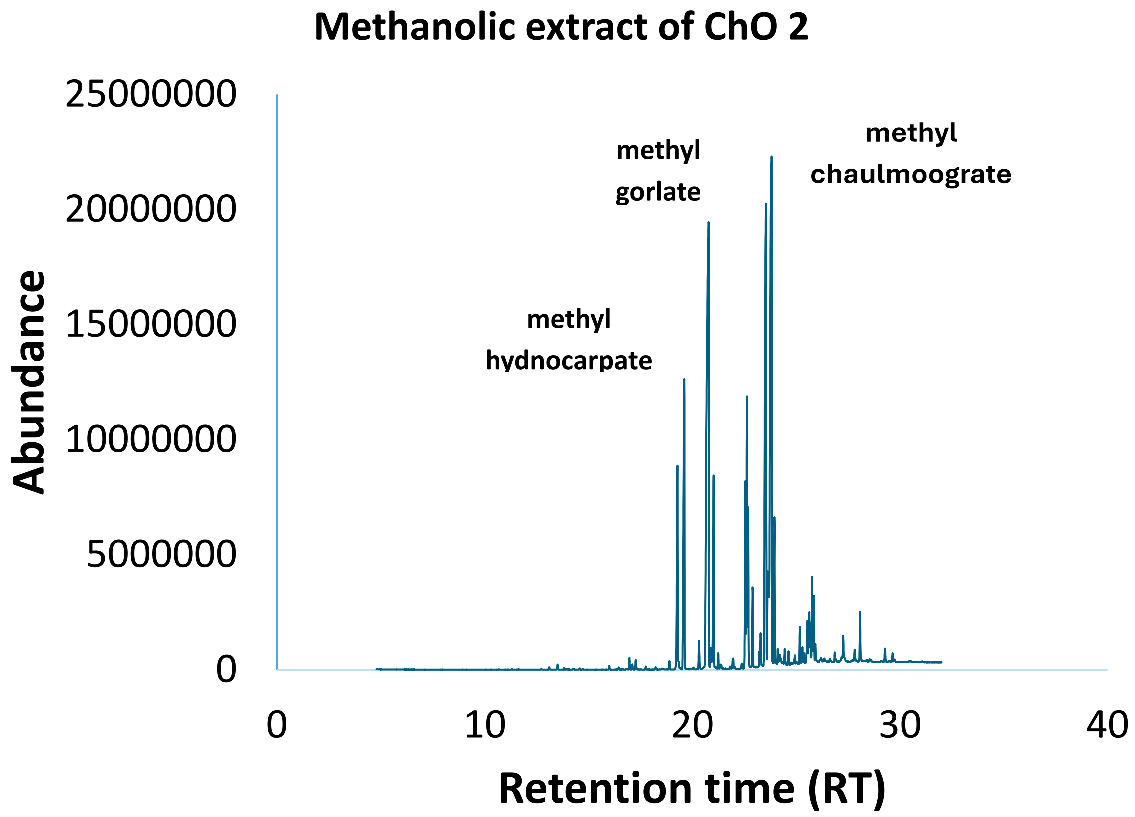

Figure S12. GC-MS chromatogram of the ChO 2 methanolic extract sample acquired on the ZB-5HT column. Identified fatty acid methyl esters are listed in Table 2 of the main text, and the numbered peak assignments are given in Table S3.

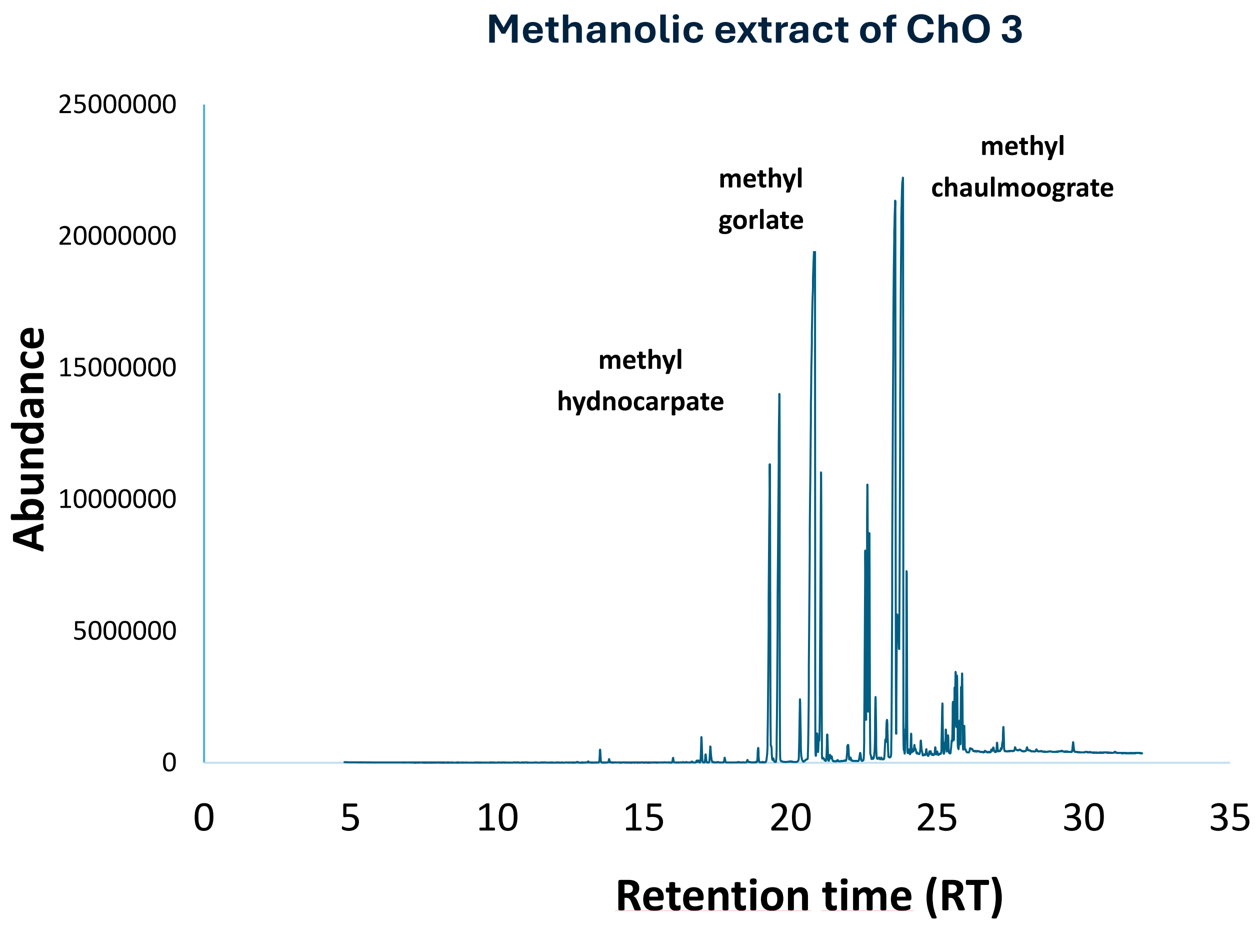

Figure S13. GC-MS chromatogram of the ChO 3 methanolic extract sample acquired on the ZB-5HT column. Identified fatty acid methyl esters are listed in Table 2 of the main text, and the numbered peak assignments are given in Table S3.

Table S3. Peak assignments for Figures S10 to S13. Peaks are listed in order of elution on the ZB-5HT column and numbered as in Table 2. Not all compounds are present in every sample, and their percentages of the total peak area are given in Table 2. Where two compounds share the same retention time to one decimal place, the order follows the experimental linear retention index reported in Table 2. The peaks at 22.5 min (numbers 6 and 7) and at 22.6 min (numbers 8 and 9) elute closely and may partially overlap.

RT (min)	Compound	Shorthand	Table 2 No.
19.2	methyl hexadec-9-enoate	16:1	1
19.6	methyl palmitate	16:0	2
20.8	methyl hydnocarpate	16:2 (cyc)	3
20.9	methyl 14-methylhexadecanoate	17:0 (br)	4
20.9	methyl dihydrohydnocarpate	16:1 (cyc)	5
22.5	methyl linoleate	18:2	6
22.5	methyl octadec-11-enoate	18:1	7
22.6	methyl oleate	18:1	8
22.6	15-octadecenoic acid, methyl ester	18:1	9
22.9	methyl stearate	18:0	10
23.5	methyl gorlate	18:3 (cyc)	11
23.8	methyl chaulmoograte	18:2 (cyc)	12

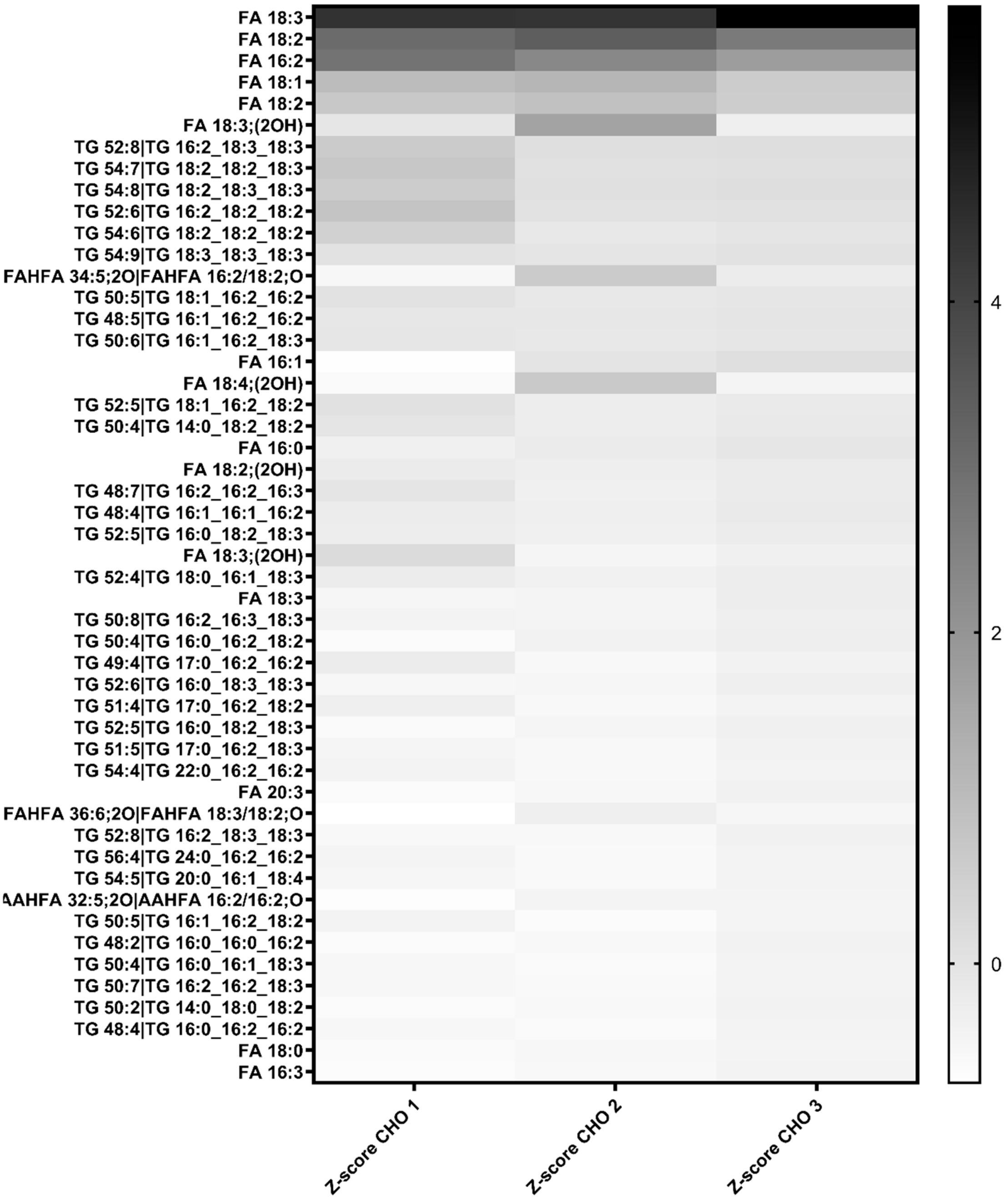
FA 18:3
FA 18:2
FA 16:2
FA 18:1
FA 18:2
FA 18:3;(2OH)
TG 52:8|TG 16:2_18:3_18:3
TG 54:7|TG 18:2_18:2_18:3
TG 54:8|TG 18:2_18:3_18:3
TG 52:6|TG 16:2_18:2_18:2
TG 54:6|TG 18:2_18:2_18:2
TG 54:9|TG 18:3_18:3_18:3
FAHFA 34:5;2O|FAHFA 16:2/18:2;O
TG 50:5|TG 18:1_16:2_16:2
TG 48:5|TG 16:1_16:2_16:2
TG 50:6|TG 16:1_16:2_18:3
FA 16:1
FA 18:4;(2OH)
TG 52:5|TG 18:1_16:2_18:2
TG 50:4|TG 14:0_18:2_18:2
FA 16:0
FA 18:2;(2OH)
TG 48:7|TG 16:2_16:2_16:3
TG 48:4|TG 16:1_16:1_16:2
TG 52:5|TG 16:0_18:2_18:3
FA 18:3;(2OH)
TG 52:4|TG 18:0_16:1_18:3
FA 18:3
TG 50:8|TG 16:2_16:3_18:3
TG 50:4|TG 16:0_16:2_18:2
TG 49:4|TG 17:0_16:2_16:2
TG 52:6|TG 16:0_18:3_18:3
TG 51:4|TG 17:0_16:2_18:2
TG 52:5|TG 16:0_18:2_18:3
TG 51:5|TG 17:0_16:2_18:3
TG 54:4|TG 22:0_16:2_16:2
FA 20:3
FAHFA 36:6;2O|FAHFA 18:3/18:2;O
TG 52:8|TG 16:2_18:3_18:3
TG 56:4|TG 24:0_16:2_16:2
TG 54:5|TG 20:0_16:1_18:4
AAHFA 32:5;2O|AAHFA 16:2/16:2;O
TG 50:5|TG 16:1_16:2_18:2
TG 48:2|TG 16:0_16:0_16:2
TG 50:4|TG 16:0_16:1_18:3
TG 50:7|TG 16:2_16:2_18:3
TG 50:2|TG 14:0_18:0_18:2
TG 48:4|TG 16:0_16:2_16:2
FA 18:0
FA 16:3
Z-score CHO 1
Z-score CHO 2
Z-score CHO 3
4
2
0

Figure S14. Heatmap of 50 annotated lipid species in three Hydnocarpus wightianus oil samples, comprising free fatty acids, oxidized free fatty acids, fatty acyl esters of hydroxy fatty acids and triacylglycerols. The colour scale represents the z-score computed within each sample, that is, the intensity of each species standardized against the 50 species shown for that sample to a mean of zero and a standard deviation of one, ranging from white (low) to black (high). The map therefore shows the standing of each species within the annotated signal of each sample, and it does not compare absolute intensities between samples. Because species of both ion modes enter one standardization, the standing of negative-mode species (FA, FAHFA, OxFA) against positive-mode species (TG) reflects their different ionization and not their relative amounts. No clustering was applied. All rows are labelled with the corresponding lipid species.

Section S1. Qualitative agar formats, well diffusion and agar incorporation

Two qualitative agar formats were applied in addition to the broth dilution assay of Section 3.6.2 of the main text. Neither carried a positive control, so their sensitivity is not known, and both are reported here as bench observations that support no conclusion.

S1.1. Well diffusion assay

A well diffusion method was employed for the initial screening of antimicrobial activity. The solvent-free extracts (prepared as described in Section 3.3.1 of the main text) and the crude oils were dissolved in dimethyl sulfoxide (DMSO) supplemented with 0.5% (w/v) Tween 80 to ensure homogeneous dispersion. The microbial suspension (0.5 mL) was spread evenly onto the surface of the solidified agar medium. Wells (5 mm diameter) were punched and filled with 20 µL of the test solutions at four concentrations: 1 mg/mL, 100 µg/mL, 50 µg/mL, and 25 µg/mL. DMSO containing 0.5% Tween 80 served as the negative control. No positive control was included in this format. The layout is reconstructed from the photographic record of the assay, which is the primary record retained for this experiment. Representative photographs of the plates are provided in Table S4. Following a 24 h incubation at the species-specific optimal temperatures, plates were inspected for zones of inhibition, which were measured with a vernier caliper where present.

S1.2. Agar incorporation assay

Because a fixed oil diffuses poorly through agar, a format was also used in which the lipid was incorporated into the medium rather than applied to its surface. Each oil was emulsified by magnetic stirring into sterile molten agar containing 0.5% (w/v) Tween 80 held at 60 °C, at a loading between 60 and 240 mg per plate. The emulsion (10 mL) was poured into Petri dishes and, after solidification, the surface was inoculated with 100 µL of the standardized microbial suspension. Control plates carried the emulsifier vehicle without the lipid phase and were prepared, inoculated and incubated in the same run. No positive control was incorporated into this format, so its sensitivity was not established and the absence of a visible difference cannot be read as the absence of an effect. The format was applied to the three crude oils and not to the methanolic extracts. It was set up for all three organisms, but the complete series, that is the vehicle control together with all three oils, was evaluable for *Candida albicans* only. In the *Escherichia coli* series the vehicle control plate showed no growth, so that series was not evaluable, and in the *Bacillus cereus* series the set was incomplete. Only the *C. albicans* series is therefore reported. The plates carry a single loading within the stated range, the exact value of which was not recorded. After 24 h of incubation at the species-specific optimal temperature the plates were compared visually with the control. The inoculum, 100 µL of the standardized suspension, corresponds to approximately 10^5 colony-forming units per plate, at which density the growth is confluent rather than resolved into discrete colonies. The endpoint is therefore a visual comparison of the density of confluent growth, no counts were made, and a comparison of this kind resolves only near-complete inhibition.

S1.3. Observations

In the well diffusion screening, no measurable zones of inhibition were detected, with the exception of faint and equivocal clearing at the highest load tested (1 mg/mL) on *C. albicans*, which did not form a well-defined zone (Table S4). The lawn was fully confluent for *B. cereus* but not for *C. albicans*, and on a lawn that is not confluent a faint clearing cannot be distinguished from local variation in the density of the inoculum. No interpretation is placed on it. For *E. coli* a single plate was retained, and its lawn density cannot be established from that record. The faint clearing seen on *C. albicans* was not reproduced in the broth dilution assay. The mass applied per well, at most 20 μg, was twenty-five times lower than the 500 μg present in each tube of the broth dilution assay.

The agar incorporation format avoids the diffusion limitation. Its loading, 60 to 240 mg per 10 mL plate, corresponds to 0.6 to 2.4% (w/v), against 0.025% (w/v) for the highest concentration applied in the broth dilution assay and approximately 5% for the lipid phase of the patented carriers referred to in Section 2.4 of the main text. At that loading the colony density on plates containing any of the three oils was indistinguishable from that on the vehicle control plate prepared in the same run (Figure S15). The comparison is confined to *C. albicans*, the one organism for which the complete series was obtained, and, in the absence of a positive control, it establishes only that none of the three oils produced near-complete inhibition at this loading.

Table S4. Representative photographs of the agar well diffusion assay. (A) ChO 1 on an Escherichia coli lawn. (B) ChO 3 on a Bacillus cereus lawn. (C) ChO 1 on a Candida albicans lawn. (D) Methanolic extracts on Candida albicans lawns, from left to right ChO 3, ChO 1 and ChO 2. Wells were loaded with 20 µL of the test article at the concentrations given in Section S1.1, the four concentrations being distributed over separate plates carrying one to three wells each. No well-defined zone of inhibition was observed on any plate. The lawn was fully confluent for B. cereus but not for C. albicans, and for E. coli a single plate was retained, so the assay is reported as a qualitative screen.

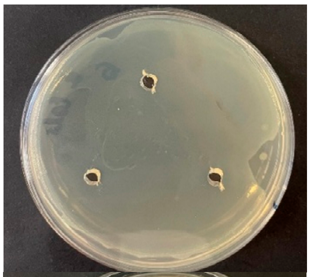

(A) ChO 1 on an Escherichia coli lawn. No zone of inhibition.

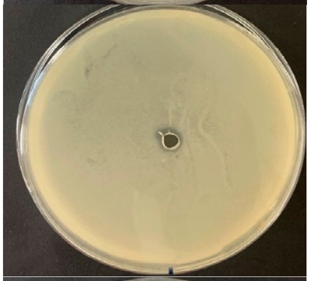

(B) ChO 3 on a Bacillus cereus lawn. No zone of inhibition.

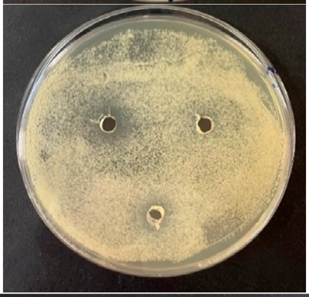

(C) ChO 1 on a Candida albicans lawn. No well-defined zone of inhibition.

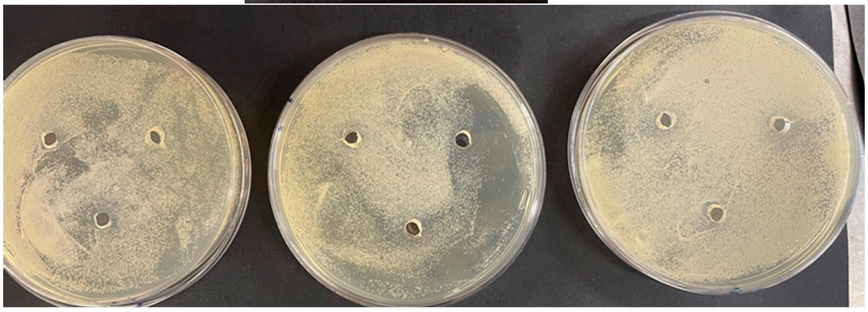

(D) Methanolic extracts of ChO 3, ChO 1 and ChO 2, from left to right, on Candida albicans lawns. No well-defined zone of inhibition.

Table S5. Mean optical density (OD600) of cultures exposed to the investigated oils (ChO 1 to ChO 3), their methanolic extracts, and reference standards against the selected strains.

Sample	Concentration [µg/mL]	*C. albicans* (OD_{600})	*E. coli* (OD_{600})	*B. cereus* (OD_{600})
**ChO 1 (Supplier A)**	250	2.011 ± 0.006	1.257 ± 0.001	1.466 ± 0.004
	125	2.001 ± 0.016	1.173 ± 0.002	1.462 ± 0.124
	62.5	2.018 ± 0.016	1.163 ± 0.003	1.304 ± 0.007
	31.25	1.996 ± 0.037	1.229 ± 0.002	1.414 ± 0.003
	15.63	2.005 ± 0.080	1.228 ± 0.001	1.324 ± 0.027
	7.81	1.917 ± 0.114	1.174 ± 0.002	1.350 ± 0.002
	3.91	1.997 ± 0.045	1.211 ± 0.001	1.451 ± 0.003
	1.95	1.982 ± 0.024	1.266 ± 0.068	1.456 ± 0.148
**ChO 2 (Supplier B)**	250	1.942 ± 0.009	1.114 ± 0.022	1.563 ± 0.001
	125	1.905 ± 0.056	1.173 ± 0.046	1.319 ± 0.026
	62.5	1.888 ± 0.084	1.173 ± 0.001	1.304 ± 0.082
	31.25	1.906 ± 0.103	1.224 ± 0.002	1.778 ± 0.002
	15.63	1.986 ± 0.033	1.336 ± 0.001	1.319 ± 0.006
	7.81	1.956 ± 0.005	1.232 ± 0.001	1.344 ± 0.002
	3.91	1.947 ± 0.023	1.261 ± 0.001	1.351 ± 0.066
	1.95	1.940 ± 0.038	1.362 ± 0.006	1.401 ± 0.046
**ChO 3 (Supplier C)**	250	2.023 ± 0.007	1.206 ± 0.002	1.429 ± 0.003
	125	2.026 ± 0.009	1.425 ± 0.401	1.381 ± 0.062
	62.5	2.016 ± 0.005	1.145 ± 0.008	1.508 ± 0.003
	31.25	2.066 ± 0.005	1.220 ± 0.002	1.405 ± 0.057
	15.63	2.006 ± 0.002	1.254 ± 0.004	1.317 ± 0.001
	7.81	2.031 ± 0.012	1.198 ± 0.001	1.289 ± 0.002
	3.91	2.057 ± 0.015	1.190 ± 0.002	1.337 ± 0.003
	1.95	2.176 ± 0.211	1.197 ± 0.001	1.305 ± 0.022
**ChO 1 MeOH extract**	250	2.055 ± 0.065	0.948 ± 0.002	1.430 ± 0.016
	125	2.037 ± 0.024	0.999 ± 0.002	1.699 ± 0.148
	62.5	2.030 ± 0.014	1.080 ± 0.002	1.472 ± 0.101
	31.25	2.026 ± 0.009	1.146 ± 0.006	1.306 ± 0.007
	15.63	2.057 ± 0.007	1.218 ± 0.006	1.332 ± 0.020
	7.81	2.010 ± 0.047	1.192 ± 0.006	1.289 ± 0.004
	3.91	2.004 ± 0.035	1.231 ± 0.146	1.347 ± 0.048
	1.95	2.239 ± 0.330	1.140 ± 0.004	1.348 ± 0.036
**ChO 2 MeOH extract**	250	1.981 ± 0.016	0.951 ± 0.015	1.334 ± 0.015
	125	1.970 ± 0.081	1.043 ± 0.002	1.453 ± 0.028
	62.5	1.916 ± 0.157	1.131 ± 0.001	1.361 ± 0.023
	31.25	2.022 ± 0.038	1.193 ± 0.001	1.340 ± 0.013
	15.63	2.010 ± 0.012	1.203 ± 0.001	1.375 ± 0.008
	7.81	2.007 ± 0.061	1.135 ± 0.001	1.377 ± 0.083
	3.91	1.944 ± 0.025	1.190 ± 0.001	1.303 ± 0.013
	1.95	1.970 ± 0.019	1.222 ± 0.033	1.308 ± 0.009
**ChO 3 MeOH extract**	250	2.047 ± 0.031	0.880 ± 0.006	1.482 ± 0.019
	125	2.056 ± 0.010	0.945 ± 0.002	1.486 ± 0.011
	62.5	2.035 ± 0.015	1.076 ± 0.007	1.414 ± 0.003
	31.25	2.050 ± 0.019	1.199 ± 0.105	1.330 ± 0.039

	15.63	2.022 ± 0.007	1.131 ± 0.003	1.349 ± 0.038
	7.81	2.065 ± 0.023	1.094 ± 0.002	1.349 ± 0.004
	3.91	2.069 ± 0.043	1.120 ± 0.001	1.356 ± 0.047
	1.95	2.062 ± 0.009	1.141 ± 0.001	1.332 ± 0.040
**Clotrimazole (positive control)**	62.5	0.496 ± 0.036	ND	ND
	31.25	0.524 ± 0.025	ND	ND
	15.63	0.621 ± 0.002	ND	ND
	7.81	0.857 ± 0.004	ND	ND
	3.91	1.663 ± 0.005	ND	ND
	1.95	1.688 ± 0.026	ND	ND
**Neomycin (positive control)**	62.5	ND	0.164 ± 0.002	0.055 ± 0.010
	31.25	ND	0.961 ± 0.073	0.841 ± 0.007
	15.63	ND	0.399 ± 0.003	0.930 ± 0.016
	7.81	ND	1.092 ± 0.001	0.946 ± 0.025
	3.91	ND	1.061 ± 0.001	0.993 ± 0.005
	1.95	ND	1.148 ± 0.002	1.077 ± 0.014
**Controls**	Solvent (DMSO + 0.5% Tween 80)	2.014 ± 0.025	1.169 ± 0.000	1.294 ± 0.007
	Culture medium	2.055 ± 0.020	1.049 ± 0.079	1.625 ± 0.038

Each value is the mean ± SD of three replicate determinations (n = 3) of a single culture per preparation and concentration. The experimental unit is the culture and not the individual determination. ND, not determined: clotrimazole was tested only against C. albicans and neomycin only against the bacterial strains. The dispersion between determinations is at or below 0.003 OD units in 51 of the 168 cells of this table and exceeds 5% of the mean in 19 of them, the largest value being 1.425 ± 0.401 for ChO 3 against E. coli at 125 µg/mL, which is noted in the legend of Table S7. The three vehicle determinations for E. coli each returned 1.169, so the dispersion of that set lies below the resolution at which absorbance was recorded rather than at zero.

Table S6. Effect of *Hydnocarpus wightianus* oils and their methanolic extracts on the optical density (OD_{600}) of *Candida albicans*, *Escherichia coli* and *Bacillus cereus* cultures, assessed by one-way ANOVA with Dunnett's two-sided multiple-comparison test against the vehicle control.

Sample	n	Mean OD_{600} ± SD	Mean difference	Dunnett *p*
Candida albicans One-way ANOVA: $F(7,157) = 72.3$, $p < 0.001$, $\eta^2 = 0.76$.				
Vehicle (DMSO + 0.5% Tween 80)	3	2.014 ± 0.025	ref.	ref.
ChO 1	24	1.991 ± 0.055	−0.023	1.000
ChO 2	24	1.934 ± 0.055	−0.080	0.977
ChO 3	24	2.050 ± 0.082	+0.036	1.000
ChO 1 MeOH extract	24	2.057 ± 0.124	+0.043	0.999
ChO 2 MeOH extract	24	1.978 ± 0.067	−0.036	1.000
ChO 3 MeOH extract	24	2.051 ± 0.024	+0.037	1.000
Clotrimazole (positive control)	18	0.975 ± 0.524	−1.039	< 0.001
Escherichia coli One-way ANOVA: $F(7,157) = 15.0$, $p < 0.001$, $\eta^2 = 0.40$.				
Vehicle (DMSO + 0.5% Tween 80)	3	1.169 ± 0.000	ref.	ref.
ChO 1	24	1.212 ± 0.043	+0.043	0.998
ChO 2	24	1.235 ± 0.082	+0.066	0.982
ChO 3	24	1.229 ± 0.143	+0.060	0.989
ChO 1 MeOH extract	24	1.119 ± 0.107	−0.050	0.996
ChO 2 MeOH extract	24	1.134 ± 0.090	−0.035	1.000
ChO 3 MeOH extract	24	1.073 ± 0.107	−0.096	0.885
Neomycin (positive control)	18	0.804 ± 0.392	−0.365	0.002
Bacillus cereus One-way ANOVA: $F(7,157) = 33.8$, $p < 0.001$, $\eta^2 = 0.60$.				
Vehicle (DMSO + 0.5% Tween 80)	3	1.294 ± 0.007	ref.	ref.
ChO 1	24	1.403 ± 0.086	+0.109	0.778
ChO 2	24	1.422 ± 0.163	+0.128	0.633
ChO 3	24	1.371 ± 0.075	+0.077	0.949
ChO 1 MeOH extract	24	1.403 ± 0.141	+0.109	0.781
ChO 2 MeOH extract	24	1.356 ± 0.054	+0.062	0.983
ChO 3 MeOH extract	24	1.387 ± 0.067	+0.093	0.880
Neomycin (positive control)	18	0.807 ± 0.354	−0.487	< 0.001

Data are mean ± standard deviation of three replicate readings ($n = 3$) of a single culture per preparation and concentration. Because the readings come from a single culture, the p-values describe the dispersion between readings and are not evidence of reproducible biological effects. For each strain the data were first analysed by two-way ANOVA with preparation and concentration as fixed factors. For Candida albicans neither the concentration term ($F(7,96) = 1.80$, $p = 0.095$) nor the preparation by concentration interaction ($F(35,96) = 1.05$, $p = 0.42$) reached significance, so the eight concentrations were pooled ($n = 24$) and the six antibiotic concentrations pooled ($n = 18$) for the comparison against the vehicle. For Escherichia coli and Bacillus cereus both terms were significant (concentration $F(7,96) = 14.55$ and 21.24, interaction $F(35,96) = 3.71$ and 11.73, respectively, all $p < 0.001$), and the concentration-resolved analysis is reported in Table S7. The pooled analysis presented here is a summary of the overall exposure and is not used to exclude effects at individual concentrations. For each strain, OD600 was analysed by one-way ANOVA followed by Dunnett's two-sided multiple-comparison test against the DMSO with 0.5% Tween 80 vehicle control. The three vehicle replicates for E. coli were identical (1.169), hence SD = 0.000.

Residuals departed from normality (Shapiro-Wilk $W = 0.64$, 0.85 and 0.79 for *C. albicans*, *E. coli* and *B. cereus*, respectively, all $p < 0.001$) and variances were heterogeneous owing to the antibiotic controls (Levene $p < 0.001$ for *C. albicans* and *E. coli*, $p = 0.003$ for *B. cereus*). The Kruskal-Wallis test ($H = 113.4$, 83.3 and 55.5, respectively, all $p < 0.001$) with Dunn's post-hoc test (Holm-adjusted) gave the same pattern of results. A two-sided $p < 0.05$ was considered significant. For the reference antibiotics, the reduction of OD600 relative to the vehicle, with its 95% confidence interval and Hedges' g, was 1.04 units (95% CI 0.78 to 1.30, $g = 2.01$) for clotrimazole against *C. albicans*, 0.37 units (95% CI 0.17 to 0.56, $g = 0.95$) for neomycin against *E. coli* and 0.49 units (95% CI 0.31 to 0.66, $g = 1.40$) for neomycin against *B. cereus*.

Abbreviations: CI, confidence interval. OD_{600}, optical density at 600 nm. ref., reference (vehicle) group.

Table S7. **Concentration-resolved effect of Hydnocarpus wightianus oils and their methanolic extracts on the optical density (OD600) of Escherichia coli and Bacillus cereus cultures.** Values are the percentage change of OD600 relative to the DMSO with 0.5% Tween 80 vehicle control. Each preparation and concentration combination (n = 3) was compared against the vehicle control (n = 3) by Dunnett's two-sided test, and comparisons significant at $p < 0.05$ are marked with a dagger and shown in bold. Because the replicate readings come from a single culture, the marked comparisons describe the dispersion between readings and are not evidence of reproducible biological effects. Concentrations are final concentrations in the culture, expressed in µg/mL. Spearman rho is the rank correlation between OD600 and concentration over all twenty-four determinations per preparation, and it is therefore not reproducible from the means listed in Table S5. Candida albicans is not included, because for that strain neither the concentration term nor the preparation by concentration interaction reached significance, and its results are given in the pooled analysis of Table S6. Dunnett's test uses the error variance pooled across all preparation and concentration combinations, so a group whose own replicates are widely scattered can still be flagged. This applies to ChO 3 at 125 µg/mL against E. coli, where the three replicates gave 1.425 ± 0.401. Percentage deviations were computed from the unrounded means and may differ by up to 0.1 percentage point from values recalculated using the rounded means listed in Table S5.

Preparation	250	125	62.5	31.25	15.63	7.81	3.91	1.95	Spearman rho
E. coli **vehicle control OD600 = 1.169**									
ChO 1	+7.5	+0.3	-0.5	+5.1	+5.0	+0.4	+3.6	+8.3	-0.064
ChO 2	-4.7	+0.3	+0.4	+4.7	+14.3	+5.4	+7.9	**+16.5 †**	-0.904
ChO 3	+3.1	**+21.9 †**	-2.1	+4.4	+7.3	+2.5	+1.8	+2.4	+0.171
ChO 1 MeOH	**-18.9 †**	**-14.5 †**	-7.6	-1.9	+4.2	+2.0	+5.3	-2.5	-0.679
ChO 2 MeOH	**-18.6 †**	-10.7	-3.3	+2.1	+2.9	-2.9	+1.8	+4.6	-0.781
ChO 3 MeOH	**-24.8 †**	**-19.1 †**	-8.0	+2.5	-3.3	-6.4	-4.2	-2.4	-0.749
B. cereus **vehicle control OD600 = 1.294**									
ChO 1	**+13.3 †**	**+12.9 †**	+0.7	+9.2	+2.3	+4.3	**+12.1 †**	**+12.5 †**	+0.095
ChO 2	**+20.8 †**	+1.9	+0.7	**+37.4 †**	+1.9	+3.8	+4.4	+8.3	+0.076
ChO 3	**+10.4 †**	+6.7	**+16.5 †**	+8.6	+1.8	-0.4	+3.3	+0.8	+0.728
ChO 1 MeOH	**+10.5 †**	**+31.3 †**	**+13.8 †**	+0.9	+2.9	-0.4	+4.0	+4.1	+0.633

ChO 2 MeOH	+3.1	**+12.2** †	+5.2	+3.5	+6.2	+6.4	+0.6	+1.1	+0.491
ChO 3 MeOH	**+14.5** †	**+14.8** †	+9.2	+2.8	+4.2	+4.2	+4.7	+2.9	+0.754

For E. coli the three methanolic extracts had their lowest OD600 at the two highest concentrations, whereas the three crude oils lowered the OD600 by at most 4.7% (ChO 2 at 250 µg/mL). For B. cereus every significant comparison was positive, that is the optical density exceeded that of the vehicle control. The vehicle control itself gave an OD600 of 1.294 against 1.625 for the inoculated medium without vehicle. In the absence of an abiotic blank containing the test article in medium without inoculum, these values are not interpreted as evidence of growth promotion. For Candida albicans neither the concentration term nor the preparation by concentration interaction reached significance, so the pooled analysis in Table S6 is the appropriate summary for that organism.

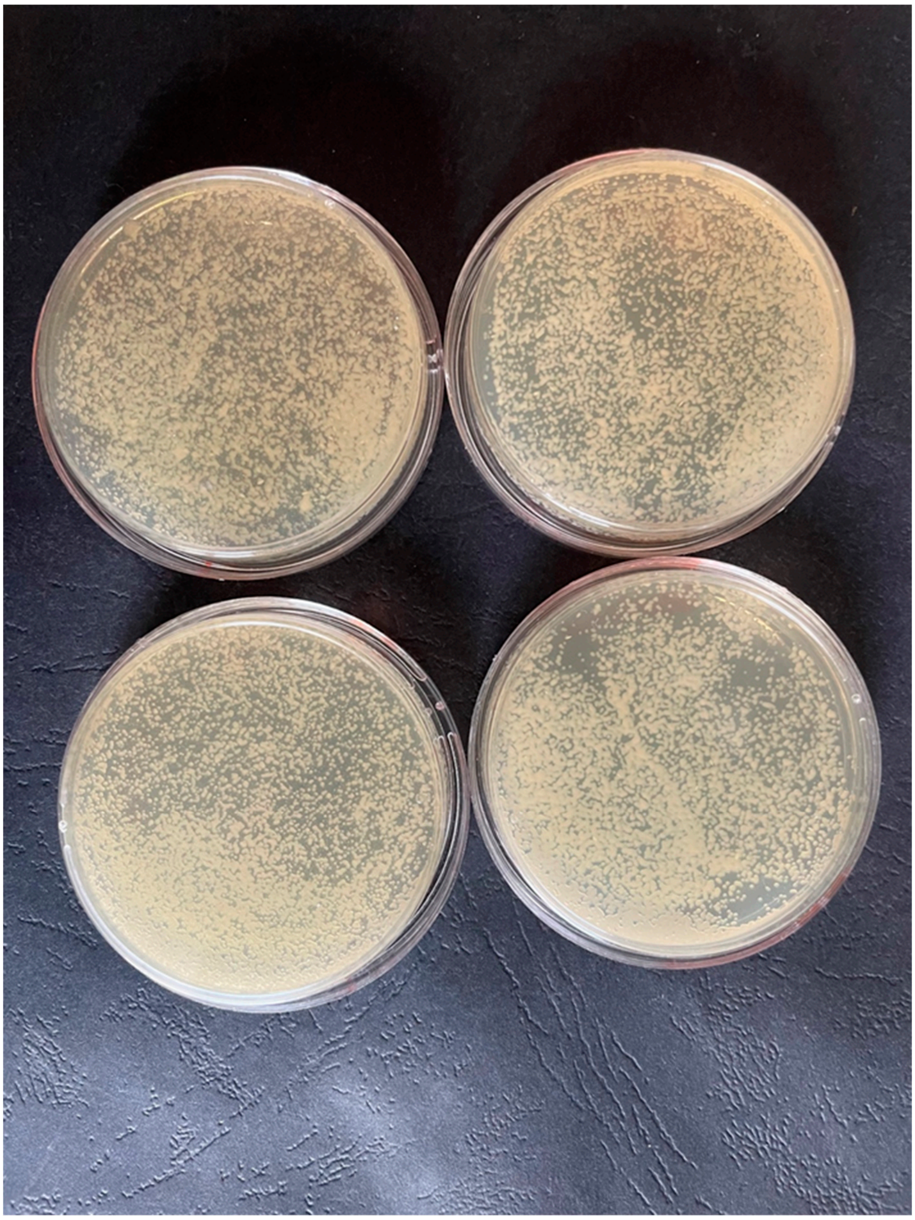

Figure S15. Agar incorporation assay against Candida albicans. Top left, vehicle control plate carrying the emulsifier without the lipid phase. Top right, ChO 1. Bottom left, ChO 2. Bottom right, ChO 3. All plates were prepared, inoculated and incubated in the same run and carry a single oil loading, the exact value of which was not recorded. The plates bear no visible identification, and the assignment above follows the position recorded at the time of photography. Colony density on the oil-containing plates is visually indistinguishable from the control. The endpoint is a visual comparison, no counts were made, and no positive control was incorporated into this format, so the comparison establishes only that none of the three oils produced near-complete inhibition at this loading.
